# EFHD2 drives lactate-mediated DNA damage repair and immunosuppression via HMGB1 and HIF-1α to confer radioresistance in colorectal cancer

**DOI:** 10.1038/s41419-026-09031-2

**Published:** 2026-06-23

**Authors:** Yilin Yu, Haixia Wu, Yu Xiao, Jianjian Qiu, Liang Hong, Baihua Yang, Jianmin Wang, Zhiping Wang, Lingdong Shao, Benhua Xu, Junxin Wu

**Affiliations:** 1 Department of Radiation Oncology, Clinical Oncology School of Fujian Medical University, Fujian Cancer Hospital (Fujian Branch of Fudan University Shanghai Cancer Center), Fuzhou, China; 2 NHC Key Laboratory of Cancer and Metabolism (Fujian Cancer Hospital), Fuzhou, China; 3 Clinical Oncology School of Fujian Medical University, Fujian Cancer Hospital (Fujian Branch of Fudan University Shanghai Cancer Center), Fuzhou, China; 4 Department of Radiation Oncology, The Second Affiliated Hospital of Chengdu Medical College Nuclear Industry 416 Hospital, Chengdu, China; 5 Innovation Center for Cancer Research, Clinical Oncology School of Fujian Medical University, Fujian Cancer Hospital (Fujian Branch of Fudan University Shanghai Cancer Center), Fuzhou, China; 6 Department of Radiotherapy, Cancer Center, The First Affiliated Hospital of Fujian Medical University, Fuzhou, China; 7 Department of Radiotherapy, National Regional Medical Center, Binhai Campus of the First Affiliated Hospital of Fujian Medical University, Fuzhou, China; 8 Department of Radiation Oncology, Fujian Medical University Union Hospital, Fuzhou, China

**Keywords:** Prognostic markers, Colorectal cancer

## Abstract

Radioresistance in colorectal cancer (CRC) remains a critical clinical challenge, with underlying mechanisms involving deoxyribonucleic acid (DNA) damage repair, metabolic reprogramming, and immune evasion. Here, we employed multi-omics approaches, including ribonucleic acid (RNA) sequencing, proteomic profiling, and lactyl-proteomics of radioresistant CRC cell models, combined with co-immunoprecipitation, molecular docking, spatial transcriptomics, and single-cell RNA sequencing. Functional validation included production of an EF-hand domain family member D2 (EFHD2) lysine 9 (K9) lactylation-specific antibody, T cell and macrophage co-culture assays, and multiplex immunofluorescence analyses, performed in vitro and in vivo using immunocompetent and immunodeficient mice, alongside patient-derived rectal cancer samples collected from our institution. We found that hsa-let-7b-5p was significantly downregulated in radioresistant CRC cells and tissues, correlating with poor prognosis. Restoration of hsa-let-7b-5p inhibited EFHD2, impaired homologous recombination through reduced activation of RAD51 recombinase (RAD51) and replication protein A2 (RPA2), prolonged phosphorylated histone H2AX at serine 139 (γH2AX) foci persistence, and increased oxidative stress and apoptosis after radiation. Mechanistically, EFHD2 interacted with high mobility group box 1 (HMGB1), enhancing lactate metabolism via hypoxia-inducible factor-1 alpha (HIF-1α) stabilization, with K9 lactylation of EFHD2 being essential for its DNA repair function. Elevated EFHD2-driven lactate reshaped the tumor immune microenvironment by promoting M2 macrophage polarization, suppressing antigen presentation, activating nuclear factor kappa-B (NF-κB) signaling, and upregulating programmed death-ligand 1 (PD-L1). Flow cytometry and immunohistochemistry revealed reduced CD4⁺/CD8⁺ T cell infiltration and increased forkhead box P3 positive (Foxp3⁺) regulatory T cells (Tregs) in EFHD2-high tumors, confirmed by spatial and single-cell transcriptomics showing immunosuppressive signatures. Importantly, combining EFHD2 knockdown with immune checkpoint blockade synergistically enhanced radiosensitivity and restored antitumor T cell responses. Collectively, these findings demonstrate that EFHD2 drives lactate-mediated immunosuppression and DNA repair to promote radioresistance in CRC, suggesting that targeting the EFHD2 axis may restore antitumor immunity and improve therapeutic outcomes.

**Figure:**
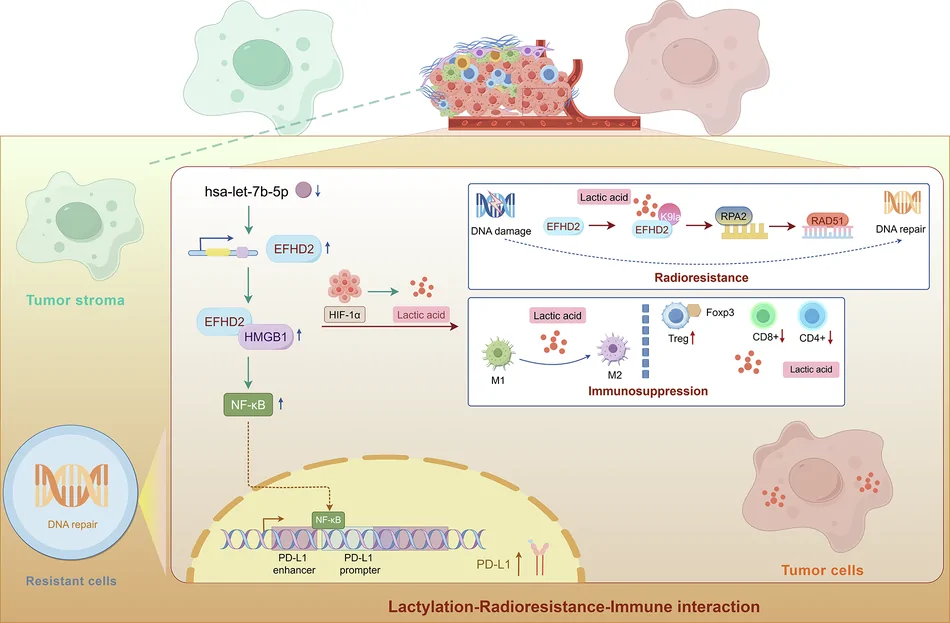

## Introduction

Globally, colorectal cancer (CRC) ranks as the third most commonly diagnosed cancer and is among the leading causes of cancer-related deaths, typically ranking second or third in mortality [1, 2]. Due to population aging and lifestyle-associated risk factors, CRC will continue to represent a significant public health challenge. Currently, the anticipated rise in the number of cases and deaths caused by rectal cancer (RC) is a cause for concern. By 2035, it is projected that the number of deaths from rectal cancer will experience a significant increase of 71.5% [3]. Although significant progress has been made in treatment, the overall survival rates for patients with RC remain unsatisfactory [4]. In recent years, radiotherapy has become widely recognized as a standard preoperative treatment method to reduce the risk of local recurrence [5]. Although there have been advancements in delivering radiation with high accuracy and tailoring radiotherapy regimens to individual patients, the emergence of radioresistance in clinical settings remains a major concern. This resistance ultimately results in disease recurrence and distant metastasis [6]. Therefore, it is imperative to investigate the fundamental mechanisms of RC and discover innovative approaches to enhance its radiosensitivity.

Tumor radioresistance is a multifaceted phenomenon influenced by both intrinsic cellular mechanisms and the tumor microenvironment (TME). One key intrinsic factor is the efficiency of DNA damage repair pathways, particularly homologous recombination (HR), which plays a crucial role in repairing radiation-induced double-strand breaks and maintaining genomic stability [7]. Beyond DNA repair, metabolic reprogramming in cancer cells has emerged as an key modulator of radiosensitivity by altering cellular redox status, energy supply, and signaling pathways [8, 9]. Furthermore, the interplay between tumor metabolism and immune regulation within the TME significantly affects therapeutic outcomes. Metabolites such as lactate can act as signaling mediators, promoting immunosuppressive phenotypes and upregulating immune checkpoint molecules, thereby fostering an environment conducive to tumor survival and therapeutic resistance [10, 11]. Elucidating the integration of metabolic and immunological mechanisms in modulating radioresistance is essential for the advancement of effective radiosensitization strategies.

To explore the mechanisms underlying radioresistance in RC, we established radioresistant cell lines and conducted integrated transcriptomic and proteomic analyses. The microRNA hsa-let-7b-5p enhances radiosensitivity by suppressing EFHD2 expression, thereby reducing HR repair efficiency and disrupting metabolic reprogramming processes that sustain an immunosuppressive tumor microenvironment. Mechanistically, EFHD2 facilitates the recruitment of RPA2 and RAD51 to DNA damage sites, and its function is modulated by lactylation at lysine 9 (K9), a modification that may affect its structural conformation or protein interactions. Knockdown of EFHD2 impaired HR activity, reduced RAD51 and RPA2 foci formation, and increased γH2AX accumulation after irradiation, collectively leading to enhanced radiosensitivity. These findings suggest that the hsa-let-7b-5p–EFHD2 axis plays a critical role in maintaining DNA repair capacity and mediating resistance to radiotherapy. In addition, EFHD2-driven lactate accumulation fosters an immunosuppressive tumor microenvironment by promoting M2 macrophage polarization, enhancing regulatory T cell infiltration, and upregulating immune checkpoint molecules such as PD-L1, thereby linking EFHD2 activity to both radioresistance and immune evasion.

## Materials and methods

### Study participants

A total of 70 biopsy-confirmed rectal cancer tissue samples were collected at Fujian Cancer Hospital between 2021 and 2025. Among these, 18 radioresistant and 37 radiosensitive cases were selected for RNA sequencing. Additionally, 9 samples underwent spatial transcriptomic analysis, and 6 samples were analyzed by immunohistochemistry. None of the patients had received any antitumor therapy prior to biopsy. No predefined exclusion criteria were applied, and no samples were excluded from the analysis during the study. The study protocol was approved by the Ethics Committee of Fujian Cancer Hospital (Fuzhou, China), and written informed consent was obtained from all participants.

### Establishment of radioresistant cell lines

The parental SW480 and HCT116 cells were subjected to fractionated radiation with a cumulative dose of 60 Gray (Gy), resulting in the generation of radioresistant cells. The resulting radioresistant SW480 and HCT116 cell lines were designated as SW480R and HCT116R for subsequent investigations. To preserve their radioresistant characteristics, the established SW480R and HCT116R cells were subsequently maintained under periodic irradiation (6 Gy, twice per week) during routine culture. For subsequent functional assays, cells were either cultured without recent irradiation to assess intrinsic properties, or subjected to additional irradiation immediately prior to the experiment when evaluating radiation response. All functional experiments were performed across multiple independent replicates, ensuring that the observed effects reflect the overall characteristics of the radioresistant cell lines rather than those of a specific subclone.

### Isolation of exosomes

Exosomes were isolated from cell culture supernatants using differential centrifugation based on a previously described protocol [12]. Cells were routinely cultured in medium supplemented with fetal bovine serum until 70–80% confluence, then washed thoroughly three to four times with 1× phosphate-buffered saline (PBS; ABK0001C, ABKBio, China) to remove residual serum. The washed cells were subsequently cultured in serum-free medium for 30 h. The cell supernatant was collected and then centrifuged at 500 × *g* for 5 min to remove intact cells, followed by further centrifugation at 2000 × *g* for 10 min to remove the cellular debris. The supernatant was then transferred to a new centrifuge tube and centrifuged at 10,000 × *g* for 30 min to remove shed microvesicles. The supernatant was then filtered through a 0.22 μm filter membrane and centrifuged at 100,000 × *g* for 2 h. For RNA isolation, the exosome pellet was washed once with 1× PBS and centrifuged again at 100,000 × *g* for 2 h again. Finally, exosomes were resuspended with 50–100 μl of 1× PBS.

### Dual-luciferase reporter assay

Wild-type (WT) and mutant (MUT) EFHD2 3′UTR reporter plasmids were constructed using the pmirGLO vector. HEK293T cells (2× 10⁴ cells/well) were seeded in 96-well plates and co-transfected with the indicated plasmids and hsa-let-7b-5p mimics or a negative control using Lipofectamine 2000 (11668-019, Thermo Fisher Scientific, USA) in triplicate. After 48 h, luciferase activity was measured using the Dual-Luciferase® Reporter Assay System (E1910, Promega, USA) according to the manufacturer’s instructions. Firefly luciferase activity was normalized to Renilla luciferase activity.

### Shotgun proteomics analysis

Excised Coomassie-stained gel bands were destained, reduced with 10 mM DTT, alkylated with 60 mM iodoacetamide, and digested overnight at 37 °C with trypsin. Peptides were extracted, lyophilized, desalted using a C18 cartridge, and resuspended in 0.1% formic acid. Samples were analyzed by LC-MS/MS using a Q Exactive HF-X mass spectrometer (Thermo Fisher Scientific, MA, USA) coupled to an Easy-nLC system. Peptides were separated on a C18 column with a 60-min linear gradient. MS data were acquired in positive ion mode using a Top10 data-dependent acquisition method. Raw data were searched using Proteome Discoverer 2.2 with Mascot against the UniProtKB/Swiss-Prot human database. Trypsin was specified with up to two missed cleavages; FDR was set to 1%. Fixed and variable modifications included carbamidomethyl (C), oxidation (M), and N-terminal acetylation.

### Co-immunoprecipitation (Co-IP) assay

Cells were washed once with PBS, and the remaining liquid was aspirated completely. Lysis buffer supplemented with protease and phosphatase inhibitors (both at 1:100 dilution) was added at 100–200 μL per 0.5–1× 10⁶ cells. Cells were lysed by gentle pipetting, and the lysate was centrifuged at 10,000–14,000 × *g* for 3–5 min at 4 °C. The supernatant was collected for further use. Magnetic beads (P2181M, Beyotime, China) were resuspended and washed twice with 1× tris-buffered saline (TBS). Beads were separated using a magnetic rack for 10 s and the supernatant was discarded each time. Beads were then resuspended in TBS to the initial volume. Protein lysate was incubated with magnetic beads (20 μL beads per 500 μL lysate) at room temperature for 2 h or overnight at 4 °C with gentle rotation. After incubation, the beads were separated magnetically and washed three times with TBS. To elute the bound proteins, SDS-PAGE loading buffer (1×) was added (100 μL per 20 μL beads), and the mixture was heated at 95 °C for 5 min. The supernatant was collected after magnetic separation and used for subsequent Western blot analysis.

### Establishment of lung metastasis model

Female BALB/c mice (3–5 weeks old, *n* = 3 per group) were purchased from Wu’s Experimental Animal Center. To establish the experimental lung metastasis model, mice were restrained gently and pre-warmed using a heating pad for 5 min to dilate the tail vein. A single-cell suspension of CT26 cells (2 × 10⁶ cells in 100 µL sterile PBS) was injected slowly into the lateral tail vein, following previously described protocols [13–17]. Successful injection was confirmed by smooth delivery without local tissue swelling or injection resistance. Mice were monitored daily for clinical signs of distress or respiratory difficulty. At the indicated time points or upon signs of morbidity, mice were humanely euthanized, and lungs were harvested for hematoxylin and eosin (H&E) staining analysis. Humane endpoints, including significant weight loss, labored breathing, or impaired mobility, were strictly observed.

### Multiplex immunohistochemistry (mIHC) staining and analysis

Multiplex immunohistochemistry was performed on human rectal cancer tissues using the AlphaXTSA® 7-color fluorescent staining kit (AXT37100041, Alpha X Bio, China) and the AlphaXPainter® automated staining system (LSI-BJ-008, Alpha X Bio, China). The staining procedure included dewaxing, antigen retrieval, and six iterative cycles of primary antibody incubation (EFHD2, HMGB1, CD68, CD163, HLA-DR, and PANCK), secondary antibody binding, and fluorophore labeling, followed by DAPI nuclear counterstaining. All incubation steps were precisely controlled by the instrument in terms of time and temperature. Antigen retrieval ensured epitope exposure, while specific primary antibodies targeted their corresponding proteins. Secondary antibodies were conjugated with fluorophores through cyclic labeling. Following six rounds of iterative labeling, the stained slides were mounted using an anti-fade medium. Fluorescent images were acquired using a whole-slide scanning system (ZEISS AxioScan 7, Carl Zeiss, Germany) equipped with ZEN 3.3 software. Quantitative analysis of EFHD2, HMGB1, CD68, CD163, HLA-DR, and PANCK expression was performed using HALO® image analysis software (version 3.5, Indica Labs, USA). Detailed staining parameters are listed in Supplementary Table 1.

### Preparation of conditioned medium and indirect co-culture system

Conditioned medium (CM) was prepared by culturing colorectal cancer cell lines in serum-free medium for 48 h after reaching ~50% confluence. The collected medium was then filtered through a 0.22 μm sterile filter (SLGPR33RB, Millipore, USA). For subsequent experiments, the CM was mixed with RPMI-1640 medium at a 1:1 ratio. To induce macrophage differentiation, THP-1 cells in the logarithmic growth phase were treated with 100 ng/mL phorbol 12-myristate 13-acetate (PMA; HY-18739, MedChemExpress, USA). After 24 h of stimulation, adherent macrophage-like cells were obtained. An indirect co-culture system was then established using a non-contact Transwell chamber (0.4 μm pore size, CLS3401, Corning, USA), in which CRC cells and macrophages were cultured in separate compartments. Specifically, macrophages were seeded in the lower chamber (bottom well), while CRC cells were cultured in the upper Transwell insert to allow paracrine signaling without direct cell-cell contact. Macrophages were harvested after co-culture for downstream analyses.

### Site-directed mutagenesis of lactylation sites in EFHD2

To investigate the functional role of EFHD2 lactylation, site-directed mutagenesis was performed to construct plasmids harboring either the wild-type (WT) EFHD2 sequence or mutations at key lysine lactylation sites of EFHD2. Specifically, lysine residues at positions 9 (K9), 188 (K188), and 233 (K233) were individually mutated to glutamic acid (E) to mimic the effect of lysine lactylation on charge and structure. These plasmids were subsequently used for functional assays to assess the impact of these specific lactylation site mutations. Detailed information on the site-specific mutations is provided in Supplementary Table 2.

### Production and purification of EFHD2 K9-lactylation-specific antibody

The EFHD2 K9-lactylation-specific antibody was custom-produced by PTM Bio (Hangzhou, China) using KLH-conjugated K9-lactylated peptides as immunogens for immunization. Two K9-lactylated peptides and one unmodified control peptide were synthesized and verified by mass spectrometry. The modified peptides were conjugated to KLH and emulsified with adjuvant for sequential immunization. Serum samples collected at predefined time points were screened for specificity and titer using ELISA and dot blot assays. Antibody-containing samples were purified by Protein A affinity chromatography, followed by positive selection with a K9-lactylated peptide affinity column and negative selection with an unmodified peptide column to eliminate non-specific binding. The final purified antibodies were quantified, supplemented with 10% glycerol, and stored at −20 °C for long-term storage.

### Assessment of intestinal villus regeneration following total body irradiation

Female BALB/c mice (9–11 weeks old, *n* = 3 per group) were housed under specific pathogen-free (SPF) conditions. Mice were pretreated via intraperitoneal injection with either sodium lactate (120 mg/kg/day, for 5 consecutive days) or LDHi (Sodium oxamate) (1,000 mg/kg, administered twice within 5 days) prior to total body irradiation (TBI, 10 Gy). After irradiation, mice received the same treatment regimen for an additional 5 days. Mice were then humanely euthanized, and small intestines were collected for histological analysis of villus length.

### Supplementary methods

Further details on the study design, experimental procedures, and statistical analyses are provided in the Supplementary Information.

### Statistical analysis

All statistical analyses and data visualization were conducted using R software (version 3.6.3). Data preprocessing and quantification were carried out using GraphPad Prism (version 10.1.2) and ImageJ (version 1.53t). Data are presented as mean ± standard error of the mean (SEM). All experiments were independently repeated at least three times unless otherwise specified. Comparisons between two groups were performed using a two-tailed Student’s *t* test or Mann–Whitney *U* test, depending on data distribution. For comparisons among more than two groups, one-way analysis of variance (ANOVA) was performed, followed by appropriate post hoc tests. The chi-square test was used to analyze differences in categorical variables. A *p*-value less than 0.05 was considered indicative of statistical significance (*p < 0.05, **p < 0.01, ***p < 0.001).

## Results

### hsa-let-7b-5p enhances radiosensitivity by promoting DNA damage and inhibiting repair

**Fig. 1:**
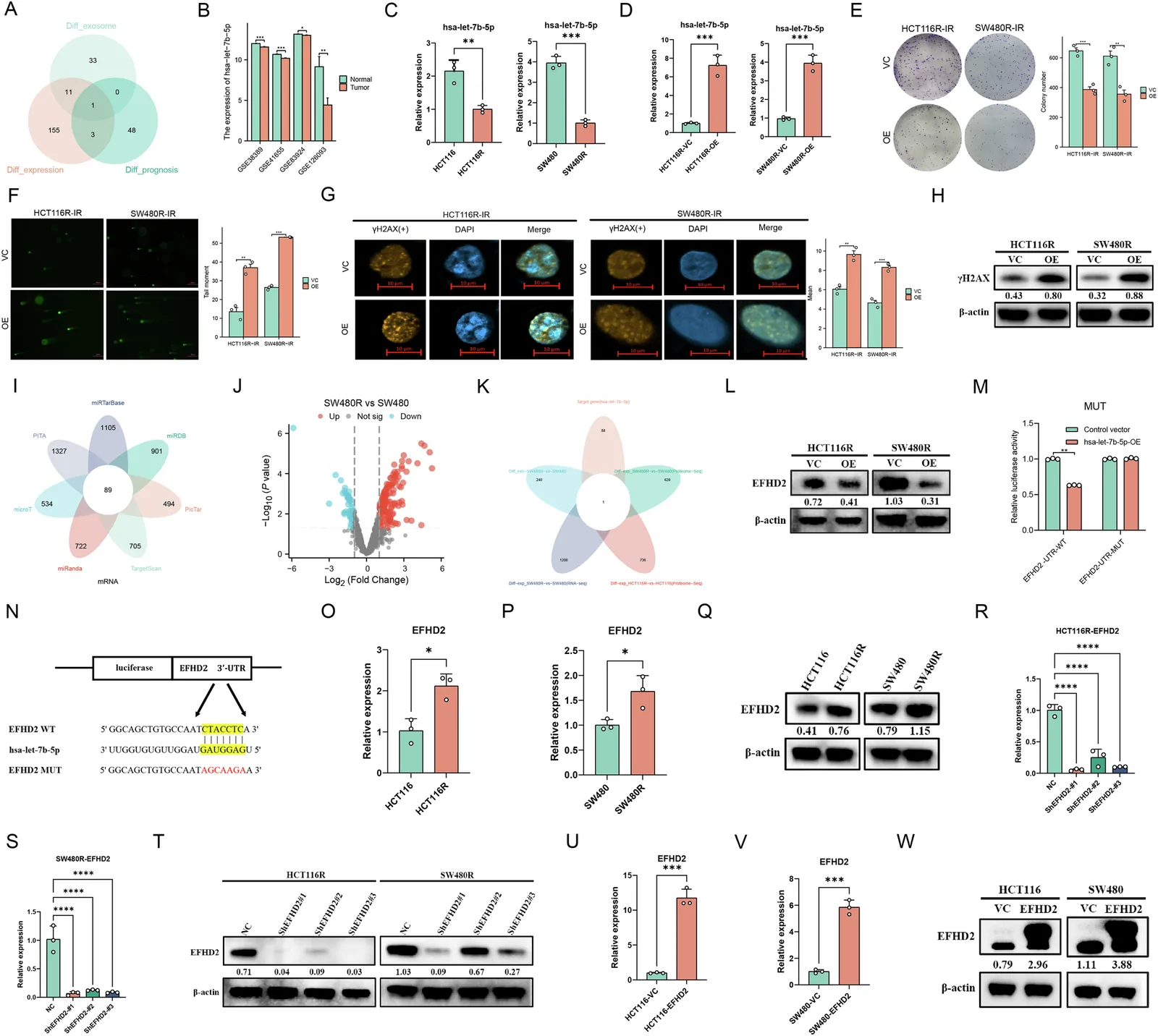
Identification of hsa-let-7b-5p as a regulator of radiosensitivity through targeting EFHD2 in colorectal cancer cells. **A** Venn diagram intersecting differentially expressed, prognostic, and exosome-enriched miRNAs, identifying hsa-let-7b-5p as a candidate. **B** Validation of hsa-let-7b-5p downregulation in colorectal cancer tissues using four GEO datasets. **C** qPCR analysis showing reduced hsa-let-7b-5p levels in radioresistant HCT116R and SW480R cells compared to parental lines. **D** Confirmation of hsa-let-7b-5p overexpression in HCT116R and SW480R cells. **E** Colony formation assays showing decreased proliferation upon hsa-let-7b-5p overexpression following ionizing radiation. **F** The comet assay demonstrating increased DNA damage in hsa-let-7b-5p-overexpressing cells post-irradiation. **G**, **H** Immunofluorescence and Western blot analyses showing elevated γH2AX levels in hsa-let-7b-5p-overexpressing cells. **I** Schematic of predicted hsa-let-7b-5p target genes using multiple online databases. **J** Proteomic analysis identifying 240 differentially expressed proteins in SW480R versus SW480 cells. **K** Intersection of predicted targets, proteomic, and transcriptomic data identifies EFHD2. **L** Western blot analysis showing downregulation of EFHD2 upon hsa-let-7b-5p overexpression. **M**, **N** Dual-luciferase reporter assays confirming direct binding of hsa-let-7b-5p to the EFHD2 3′UTR. **O**–**Q** qPCR and Western blot analyses showing higher EFHD2 expression in resistant cells. **R**–**T** Confirmation of EFHD2 knockdown efficiency by qPCR and Western blot analyses. **U**–**W** Confirmation of EFHD2 overexpression efficiency by qPCR and Western blot analyses.

We first confirmed the isolation of exosomes from SW480 and SW480R by transmission electron microscopy (Supplementary Fig. 1A) and nanoparticle tracking analysis (Supplementary Fig. 1B, C). Small RNA sequencing of exosomal miRNAs revealed 74 differentially expressed miRNAs between the two cell lines, including 45 upregulated and 29 downregulated miRNAs (Supplementary Fig. 1D, E). Functional enrichment analysis suggested their involvement in metabolic processes and DNA-related regulatory processes (Supplementary Fig. 1F–H). By intersecting differentially expressed, prognostically relevant, and exosome-enriched miRNAs, hsa-let-7b-5p was identified as a candidate of interest (Fig. 1A). Its expression was significantly reduced in colorectal cancer tissues compared to normal tissues (Supplementary Fig. 1I, J) and was validated using four GEO datasets (Fig. 1B). Pan-cancer analysis also demonstrated broadly decreased expression in tumors (Supplementary Fig. 1K). Moreover, low hsa-let-7b-5p expression was associated with poorer prognosis (Supplementary Fig. 1L), and its expression was markedly lower in HCT116R and SW480R cells compared with their respective parental HCT116 and SW480 cells (Fig. 1C). To explore its functional role, we overexpressed hsa-let-7b-5p in HCT116R and SW480R cells (Fig. 1D). Under non-irradiated conditions, overexpression had no significant effect on proliferation (Supplementary Fig. 1M). However, upon ionizing radiation, overexpression significantly reduced cell proliferation, as shown by colony formation and CCK-8 assays (Fig. 1E; Supplementary Fig. 1N, O). The overexpression group exhibited a reduced cell proportion in S phase after irradiation (Supplementary Fig. 2A), followed by increased apoptosis (Supplementary Fig. 2B). The comet assay showed increased DNA damage following radiation in hsa-let-7b-5p-overexpressing cells (Fig. 1F). Immunofluorescence staining and Western blot analysis confirmed increased γH2AX levels compared to controls (Fig. 1G, H).

### EFHD2 is identified as a downstream target of hsa-let-7b-5p and mediates radiosensitivity

We used multiple online databases to predict putative downstream targets of hsa-let-7b-5p (Fig. 1I). Proteomic analysis of exosomes derived from SW480R cells and parental SW480 cells identified 240 differentially expressed proteins (Fig. 1J). Intersection of predicted targets, differentially expressed proteins, and transcriptomic/proteomic data from SW480R and SW480 cells identified EFHD2 as a potential downstream effector (Fig. 1K). External GEO datasets further validated its elevated expression in radioresistant colorectal cancer tissues and in tumor tissues compared with normal controls (Supplementary Fig. 2C–G). Overexpression of hsa-let-7b-5p significantly reduced EFHD2 expression (Fig. 1L), and dual-luciferase reporter assays confirmed direct binding of hsa-let-7b-5p to three predicted binding sites within the EFHD2 3′UTR (Fig. 1M, N; Supplementary Fig. 2H–I). qPCR and Western blotting further showed that EFHD2 expression was significantly higher in resistant cells (Fig. 1O–Q). We next knocked down EFHD2 in resistant cells (Fig. 1R–T) and overexpressed EFHD2 in parental cells (Fig. 1U–W). Among the shRNA sequences tested, shEFHD2#1 showed the highest silencing efficiency and was selected for further experiments. Under non-irradiated conditions, EFHD2 knockdown or overexpression had no significant effect on basal cell proliferation (Supplementary Fig. 3A, B). However, upon ionizing radiation, EFHD2 knockdown significantly inhibited cell proliferation (Supplementary Fig. 3C), while EFHD2 overexpression promoted proliferation (Supplementary Fig. 3D).

### EFHD2 modulates radiation-induced cell cycle, apoptosis, and DNA damage response

**Fig. 2:**
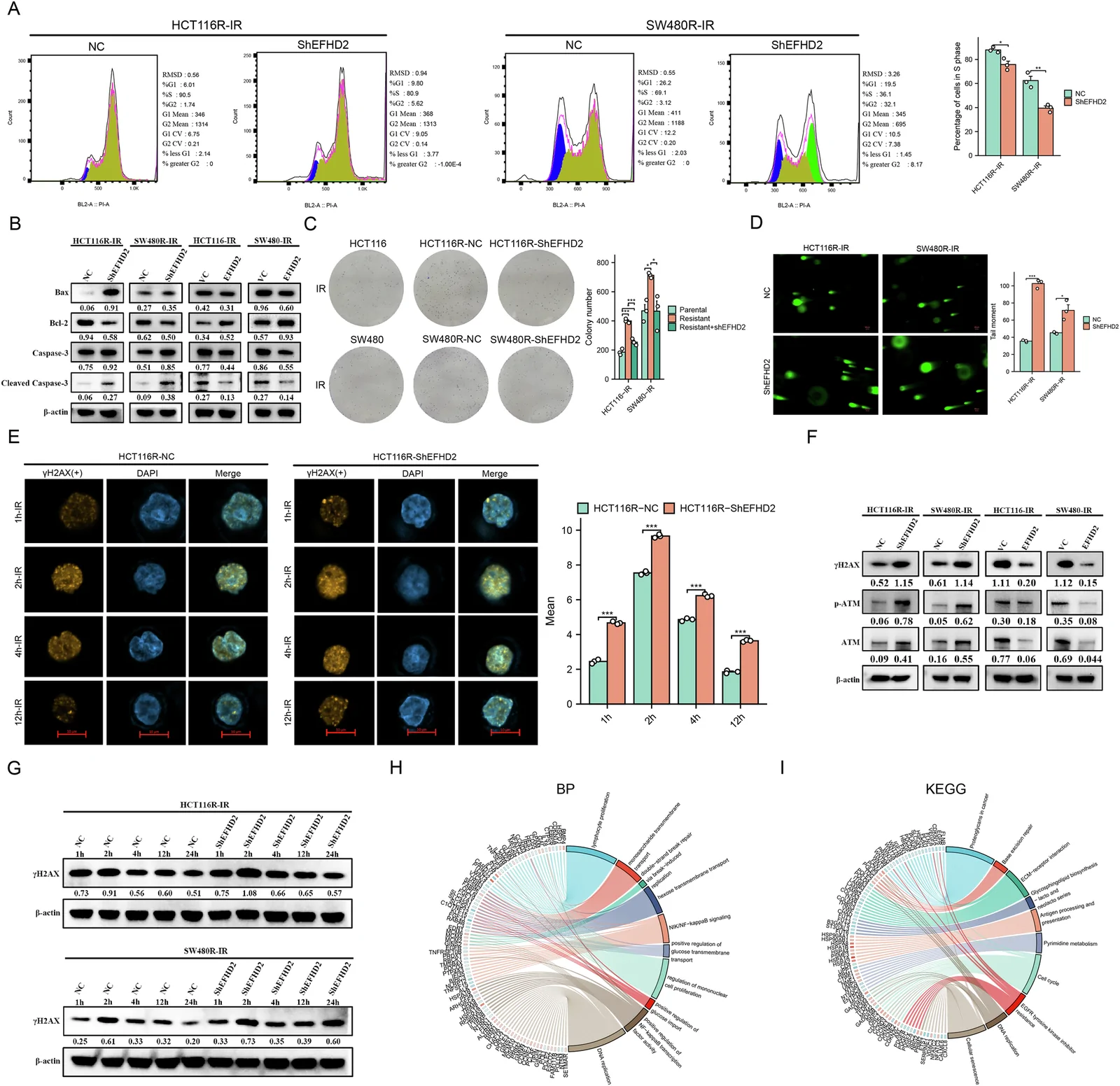
EFHD2 modulates radiation-induced cell cycle, apoptosis, proliferation, and DNA damage response. **A** Flow cytometry analysis showing that EFHD2 knockdown reduces S-phase arrest in resistant cells. **B** Western blot analysis of apoptosis-related proteins in EFHD2-manipulated cells. Knockdown of EFHD2 increased Bax and cleaved Caspase-3 levels and decreased Bcl-2, whereas EFHD2 overexpression had the opposite effect. **C** Colony formation assays confirming modulation of radiation-induced proliferation following irradiation by EFHD2. **D** The comet assay showing increased DNA damage upon EFHD2 knockdown. **E** Immunofluorescence analysis of γH2AX foci in EFHD2-knockdown cells post-irradiation. **F**, **G** Western blot and time-course analyses of γH2AX, phospho-ATM, and ATM confirming enhanced DNA damage response after EFHD2 knockdown. **H**, **I** Transcriptomic analysis of EFHD2-knockdown versus control cells.

EFHD2 knockdown reduced radiation-induced S-phase accumulation in resistant cells (Fig. 2A), while its overexpression increased S-phase accumulation in parental cells (Supplementary Fig. 3E). EFHD2 knockdown significantly increased radiation-induced apoptosis in resistant cells (Supplementary Fig. 3F), while EFHD2 overexpression reduced apoptosis in parental cells (Supplementary Fig. 3G). Western blot analysis showed that EFHD2 knockdown in resistant cells enhanced the expression of pro-apoptotic proteins Bax and cleaved Caspase-3, and suppressed the anti-apoptotic protein Bcl-2. Conversely, EFHD2 overexpression in parental cells had the opposite effect (Fig. 2B). Colony formation assays showed that proliferation following irradiation was suppressed by EFHD2 knockdown in resistant cells (Fig. 2C) and enhanced by EFHD2 overexpression in parental cells (Supplementary Fig. 3H). Additionally, we assessed DNA integrity using a comet assay. The comet assay revealed that EFHD2 knockdown significantly increased DNA damage following irradiation in resistant cells (Fig. 2D), while overexpression reduced DNA breaks in parental cells (Supplementary Fig. 4A). Immunofluorescence staining revealed more γH2AX foci in EFHD2-knockdown resistant cells, peaking at 2 h post-irradiation and gradually declining over time (Fig. 2E; Supplementary Fig. 4B). Western blotting confirmed increased levels of γH2AX, phospho-ATM, and ATM after EFHD2 knockdown in resistant cells, while these markers were decreased upon EFHD2 overexpression in parental cells (Fig. 2F). Consistently, time-course analysis showed that γH2AX levels were significantly higher in EFHD2-knockdown cells compared to control cells, peaking at 2 h after irradiation and gradually decreasing over time (Fig. 2G).

### EFHD2 interacts with HMGB1 to activate HIF-1α and regulate lactate metabolism-related genes

**Fig. 3:**
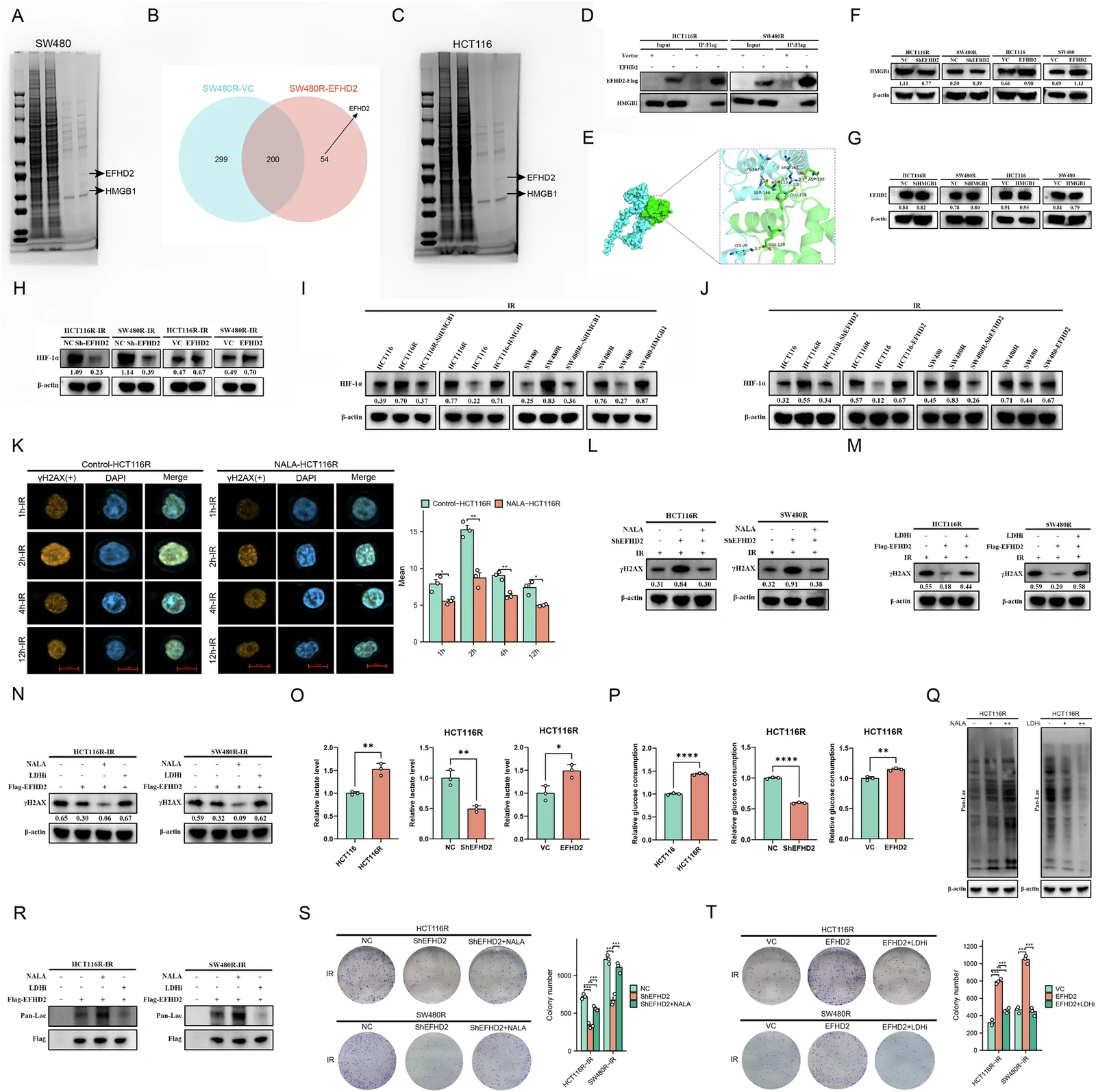
EFHD2 interacts with HMGB1 and regulates lactate metabolism and DNA damage response. **A**–**C** Coomassie staining and mass spectrometry revealed HMGB1 as a potential EFHD2-binding protein. **D** Co-immunoprecipitation confirmed the EFHD2-HMGB1 interaction. **E** Molecular docking showed multiple hydrogen bonds between EFHD2 and HMGB1 with a binding energy of −177.6 kcal/mol. **F**, **G** Western blot analyses showing that EFHD2 positively regulates HMGB1 expression, whereas HMGB1 has no effect on EFHD2. **H**–**J** Western blot analyses demonstrating that EFHD2 knockdown reduces HIF-1α expression, while overexpression increases it. **K** Immunofluorescence showing the effects of NALA treatment on γH2AX levels. **L**–**N** Western blot analysis confirming γH2AX changes upon NALA or LDHi treatment. **O**, **P** Measurement of lactate production and glucose consumption showing modulation by EFHD2 knockdown or overexpression. **Q**, **R** Effects of NALA and LDHi treatment on protein pan-lactylation and EFHD2 lactylation levels. **S**, **T** Colony formation assays indicating that LDHi partially reverses the EFHD2 overexpression-induced increase in cell proliferation following irradiation, whereas NALA partially counteracts the proliferation inhibition caused by EFHD2 knockdown.

To investigate the molecular mechanisms underlying EFHD2-mediated radioresistance, transcriptomic profiling was performed in EFHD2 knockdown and NC cells (Supplementary Fig. 4C–E). The results revealed significant changes in DNA replication, lactate metabolism, monocyte proliferation, immune response, and NF-κB pathway activity (Fig. 2H, I; Supplementary Fig. 4F, G). Proteomic analysis further supported EFHD2’s involvement in radiotherapy response, lactate metabolism, and the tumor microenvironment (Supplementary Fig. 4H–M). Coomassie staining and mass spectrometry identified HMGB1 as a potential EFHD2-interacting protein (Fig. 3A–C). Co-immunoprecipitation confirmed an interaction between EFHD2 and HMGB1 (Fig. 3D). Molecular docking indicated multiple hydrogen bonds between EFHD2 (Glu-139, Ser-146, Asp-113, Glu-116, and Asp-109) and HMGB1 (Lys-76, Lys-167, and Arg-163), with a binding energy of -177.6 kcal/mol (Fig. 3E). Western blotting revealed that EFHD2 positively regulated HMGB1 expression, whereas HMGB1 had no effect on EFHD2 (Fig. 3F, G). Since HMGB1 has been reported to activate HIF-1α and enhance lactate production, we speculated that EFHD2 regulates HMGB1, thereby activating HIF-1α and promoting lactate production. Western blot analyses showed that EFHD2 knockdown reduced HIF-1α expression, while overexpression increased it (Fig. 3H–J). We next examined the correlation between HMGB1 and lactate metabolism-related genes, including HIF-1α, LDHA, LDHB, LDHC, SLC16A1, SLC16A2, SLC16A4, and SLC16A7. HMGB1 expression showed a predominantly positive correlation with these genes (Supplementary Fig. 5A–H). Similarly, HIF-1α expression was also positively correlated with most of these genes (Supplementary Fig. 5I–O).

### EFHD2 regulates lactate metabolism and DNA damage response

**Fig. 4:**
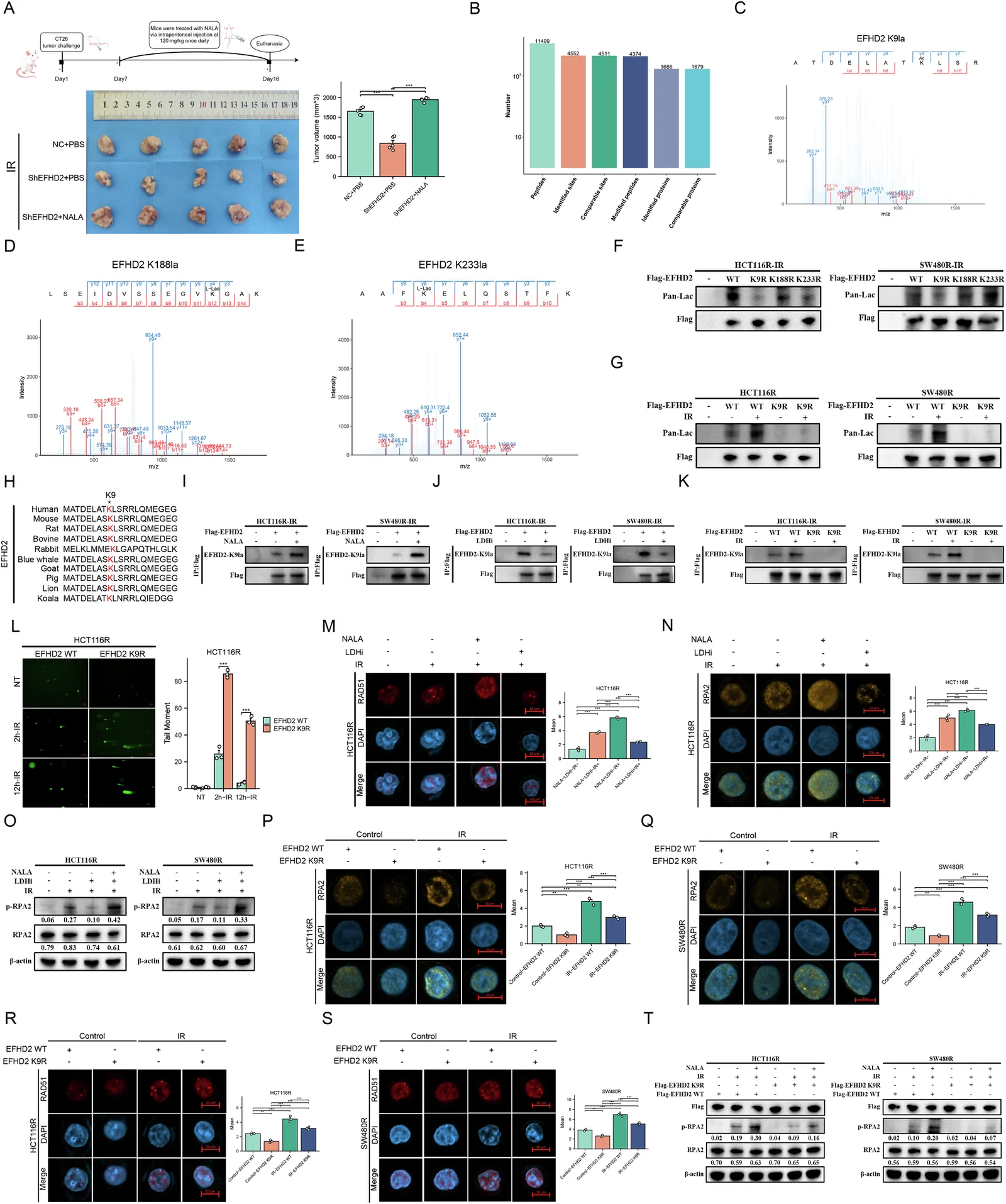
EFHD2 modulates lactate metabolism and DNA damage response via HIF-1α signaling and lysine lactylation. **A** In vivo validation showing that NALA reverses tumor growth suppression induced by EFHD2 knockdown. **B**–**E** Lactyl-proteomic analysis identifying differentially lactylated proteins and the EFHD2 K9, K188, and K233 sites, with MS/MS spectra shown. **F** Western blot analysis showing that K9 mutation most prominently reduces pan-lactylation levels. **G** The K9 mutation decreases pan-lactylation under basal and irradiated conditions. **H** Sequence alignment indicating high conservation of the K9 site across species. **I**, **J** NALA treatment enhances, whereas LDHi treatment reduces, EFHD2 K9 lactylation. **K** The K9 mutation decreases EFHD2 K9 lactylation under basal and irradiated conditions. **L** The comet assay demonstrating increased DNA damage in K9-mutant cells. **M** NALA upregulates, whereas LDHi downregulates, RAD51 expression. **N**, **O** Immunofluorescence and Western blot analyses showing modulation of RPA2 expression and phosphorylation by NALA and LDHi. **P**–**T** The K9 mutation suppresses RAD51 and RPA2 levels and reduces RPA2 phosphorylation; NALA rescues phosphorylation.

To further investigate whether modulation of lactate metabolism affects DNA damage responses, we performed immunofluorescence analysis to assess the effects of NALA and LDHi on γH2AX levels. NALA treatment reduced γH2AX levels (Fig. 3K), while LDHi increased them (Supplementary Fig. 6A), which was further confirmed by Western blot analysis (Fig. 3L–N). We then evaluated the role of EFHD2 in lactate production and glucose consumption. Lactate levels and glucose consumption rates were higher in resistant cells compared to parental cells, and both were reduced by EFHD2 knockdown and increased by EFHD2 overexpression (Fig. 3O, P; Supplementary Fig. 6B, C). We further observed that NALA treatment led to an increase in pan-lactylation levels, whereas LDHi treatment resulted in their reduction (Fig. 3Q; Supplementary Fig. 6D). Furthermore, NALA also enhanced EFHD2 lactylation, which was reduced by LDHi (Fig. 3R). Colony formation assays revealed that LDHi partially reversed the EFHD2 overexpression-induced increase in cell proliferation under irradiation, while NALA partially counteracted the proliferation inhibition caused by EFHD2 knockdown (Fig. 3S, T). These findings were further validated in vivo, where NALA reversed the tumor growth suppression induced by EFHD2 knockdown (Fig. 4A).

### The K9 lactylation of EFHD2 is critical for regulating DNA damage response

To explore the potential molecular mechanisms underlying EFHD2 function, we conducted lactyl-proteomic profiling in control and EFHD2-knockdown cells. A total of 99 upregulated and 228 downregulated lactylation sites were identified (Supplementary Fig. 6E). Figure 4B summarizes the identified peptides, modification sites, and proteins. Enrichment analysis revealed that the differentially lactylated proteins were mainly associated with metabolic and DNA-related pathways (Supplementary Fig. 6F–I). The top 30 differentially modified sites are shown in Supplementary Fig. 6J. Among them, three lactylation sites were detected on EFHD2: K9, K188, and K233, with their MS/MS spectra shown in Fig. 4C–E. Western blotting indicated that mutation at the K9 site showed the most prominent effect on pan-lactylation levels (Fig. 4F). Under both basal and irradiated conditions, mutation of the K9 site led to a marked reduction in pan-lactylation levels (Fig. 4G).

### EFHD2 regulates RPA2 phosphorylation and homologous recombination-mediated radioresistance

**Fig. 5:**
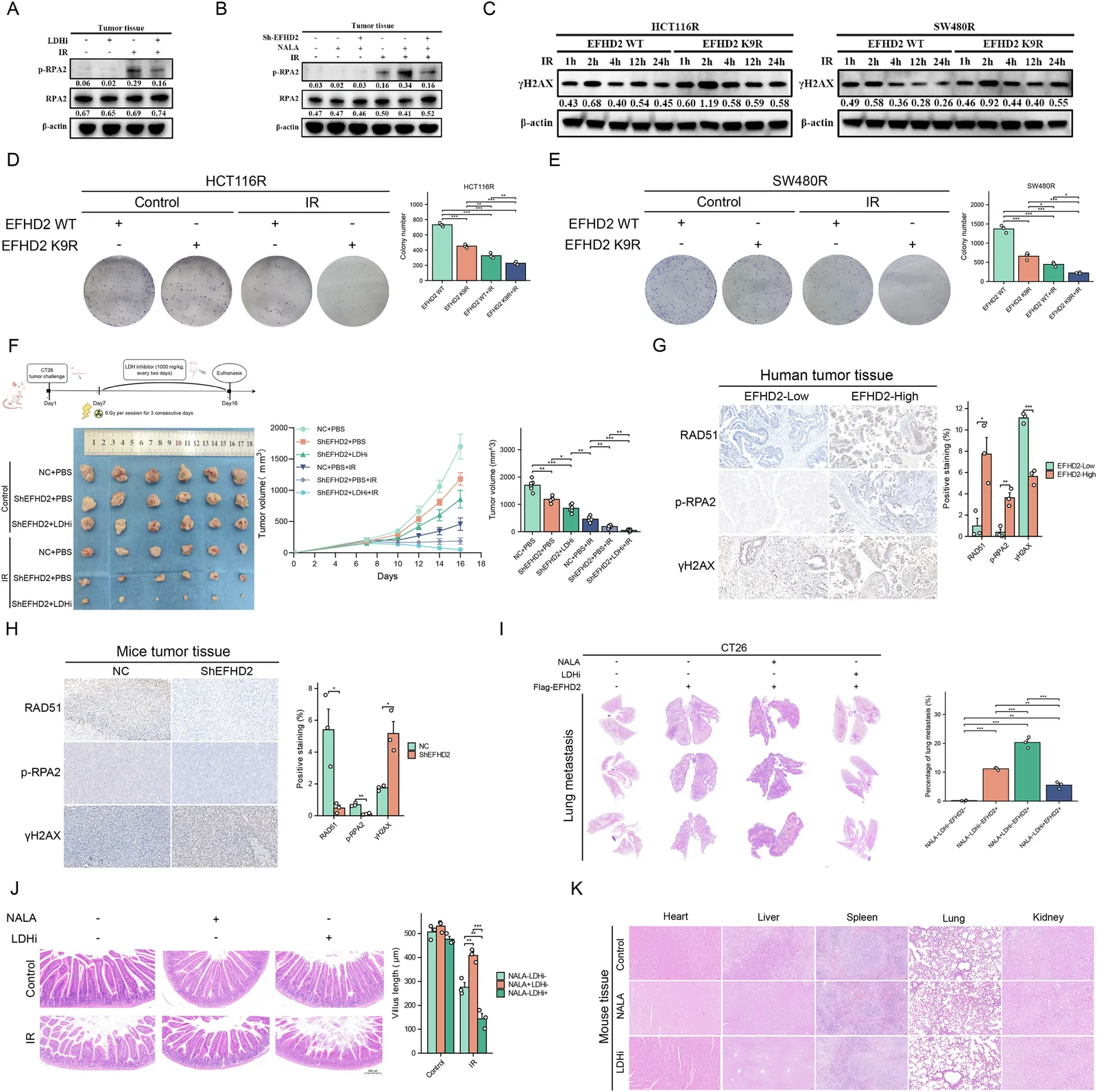
The K9 lactylation of EFHD2 is critical for DNA damage repair and homologous recombination-mediated radioresistance. **A**, **B** In tumor tissues, EFHD2 knockdown or LDHi reduces, while NALA increases, RPA2 phosphorylation post-irradiation. **C**–**E** The K9 mutation increases γH2AX levels and inhibits cell proliferation. **F** In vivo, EFHD2 knockdown or LDHi combined with irradiation significantly suppresses tumor growth. **G**, **H** IHC analysis of human rectal cancer and mouse tumor tissues showing that high EFHD2 expression is associated with increased RAD51 and p-RPA2 levels and decreased γH2AX levels. **I** Lung metastasis induced by EFHD2 overexpression or NALA treatment is reversed by LDHi treatment. **J** Intestinal regeneration assays showing that NALA enhances, whereas LDHi suppresses, villus recovery post-irradiation. **K** H&E staining of major organs showing no significant toxicity from NALA or LDHi combined with irradiation.

Further analysis revealed that the K9 site is highly conserved across species (Fig. 4H; Supplementary Table 3). To investigate the functional role of K9 lactylation, we generated a custom EFHD2 K9-lactylation–specific antibody. We observed that, under ionizing radiation, NALA enhanced the lactylation level at EFHD2 K9, whereas LDHi attenuated it (Fig. 4I, J). Moreover, mutation of the K9 site significantly reduced EFHD2 K9 lactylation under both basal culture conditions and following radiation treatment (Fig. 4K). Comet assay results demonstrated that K9 mutation significantly increased DNA damage compared to wild-type EFHD2 (Fig. 4L; Supplementary Fig. 6K). Given that EFHD2 is a component of the human Shu complex involved in RAD51 recruitment and homologous recombination, we examined the impact of NALA and LDHi on RAD51 expression. NALA upregulated RAD51, whereas LDHi suppressed it (Fig. 4M; Supplementary Fig. 6L). RAD51 is a key mediator of homologous recombination repair in response to DNA double-strand breaks induced by ionizing radiation. RAD51-mediated homologous recombination repair requires displacement of the RPA complex from ssDNA, with RPA2 serving as the key regulatory subunit. Since phosphorylation of RPA2 is essential for its replacement by RAD51, we investigated whether EFHD2 modulates RPA2 and thereby affects RAD51 activity and radioresistance. Immunofluorescence assay showed that NALA upregulated RPA2 expression, while LDHi suppressed it (Fig. 4N; Supplementary Fig. 6M), consistent with their effects on RAD51. At the protein level, NALA enhanced RPA2 phosphorylation, whereas LDHi reduced it (Fig. 4O). To further assess the role of EFHD2 K9 lactylation, we performed immunofluorescence analysis. K9 mutation significantly suppressed RAD51 and RPA2 expression compared to wild-type EFHD2 (Fig. 4P–S). Western blotting confirmed that the K9 mutation reduced RPA2 phosphorylation, while NALA increased it (Fig. 4T). In tumor tissues, LDHi reduced and NALA increased RPA2 phosphorylation under irradiation. EFHD2 knockdown also decreased RPA2 phosphorylation (Fig. 5A, B). Moreover, the EFHD2 K9 mutant led to higher and sustained γH2AX levels post-irradiation (Fig. 5C), and significantly suppressed cell proliferation (Fig. 5D, E). In vivo, EFHD2 knockdown or LDHi combined with irradiation markedly inhibited tumor growth (Fig. 5F). Immunohistochemical analysis of human rectal cancer tissues and mouse tumor specimens similarly showed that tissues with high EFHD2 expression exhibited elevated levels of RAD51 and p-RPA2, whereas γH2AX levels were reduced (Fig. 5G, H; Supplementary Fig. 6N–O). Lung metastasis induced by EFHD2 overexpression was reversed by LDHi treatment (Fig. 5I). Intestinal regeneration assays showed that NALA promoted, while LDHi suppressed, villus recovery post-irradiation (Fig. 5J). H&E staining of major organs revealed no obvious histopathological toxicity from NALA or LDHi combined with irradiation (Fig. 5K).

### Single-cell RNA-seq reveals an association between EFHD2 expression and M2 macrophage polarization

Based on transcriptome enrichment analysis indicating a potential association of EFHD2 with monocytes and the tumor immune microenvironment, we performed single-cell RNA-seq analysis. We integrated 108 single-cell RNA-seq samples using Harmony and performed UMAP clustering, identifying nine cell clusters (Supplementary Fig. 7A). Marker gene expression and cell annotation categorized these clusters into eight major cell types: T cells, myeloid cells, endothelial cells, fibroblasts, B cells, plasma cells, epithelial cells, and proliferative cells (Supplementary Fig. 7B). The distribution of these clusters across all samples was shown in Supplementary Fig. 7C. EFHD2 expression was mainly enriched in myeloid, T, and epithelial cells, with higher expression observed in colorectal cancer tissues compared to normal tissues (Supplementary Fig. 7D, E). Cells were further classified into EFHD2-positive and EFHD2-negative groups (Supplementary Fig. 7F). Myeloid, proliferative, and epithelial cells were enriched in the EFHD2-positive group, whereas B, plasma, and T cells were reduced (Supplementary Fig. 7G), suggesting a link between high EFHD2 expression, tumor proliferation, and immune suppression. We further found that the proportion of EFHD2-positive cells was higher in tumor tissues compared to normal tissues (Supplementary Fig. 7H). Given the known association between EFHD2 and macrophage polarization, we further subclustered myeloid cells into 12 subsets based on marker expression (Supplementary Fig. 7I, J), and visualized their distribution by EFHD2 expression (Supplementary Fig. 7K). The EFHD2-positive group exhibited higher proportions of M2, Macro-NUPR1, and Mono-S100A8 cells, and lower levels of Activated-DC, Macro_C1QC, Macro-SELENOP, MAST-TPSAB1, and neutrophils (Supplementary Fig. 7L). These findings support the association of EFHD2 with M2 polarization, an immunosuppressive microenvironment, and reduced antigen presentation. Single-cell level analyses further confirmed that M2 polarization scores were positively correlated with EFHD2 expression, with the highest scores observed in the M2 macrophage subset (Supplementary Fig. 7M–Q). Pseudotime trajectory analysis revealed a transitional trajectory from immune-activating to immunosuppressive phenotypes, with EFHD2-positive cells enriched at the terminal, immunosuppressive states (Supplementary Fig. 8A–C). Additionally, cell-cell communication analysis revealed enhanced interactions in the EFHD2-positive group, particularly among M2 macrophages and other cell types. EFHD2-positive cells displayed increased signaling via immunosuppressive and ECM-remodeling pathways such as TGF-β, IL-10, IL-6, Tenascin, and SPP1 (Supplementary Fig. 8D–P), highlighting EFHD2’s role in promoting M2 polarization and tumor-associated immune suppression.

### EFHD2 regulates macrophage polarization through lactate metabolism

**Fig. 6:**
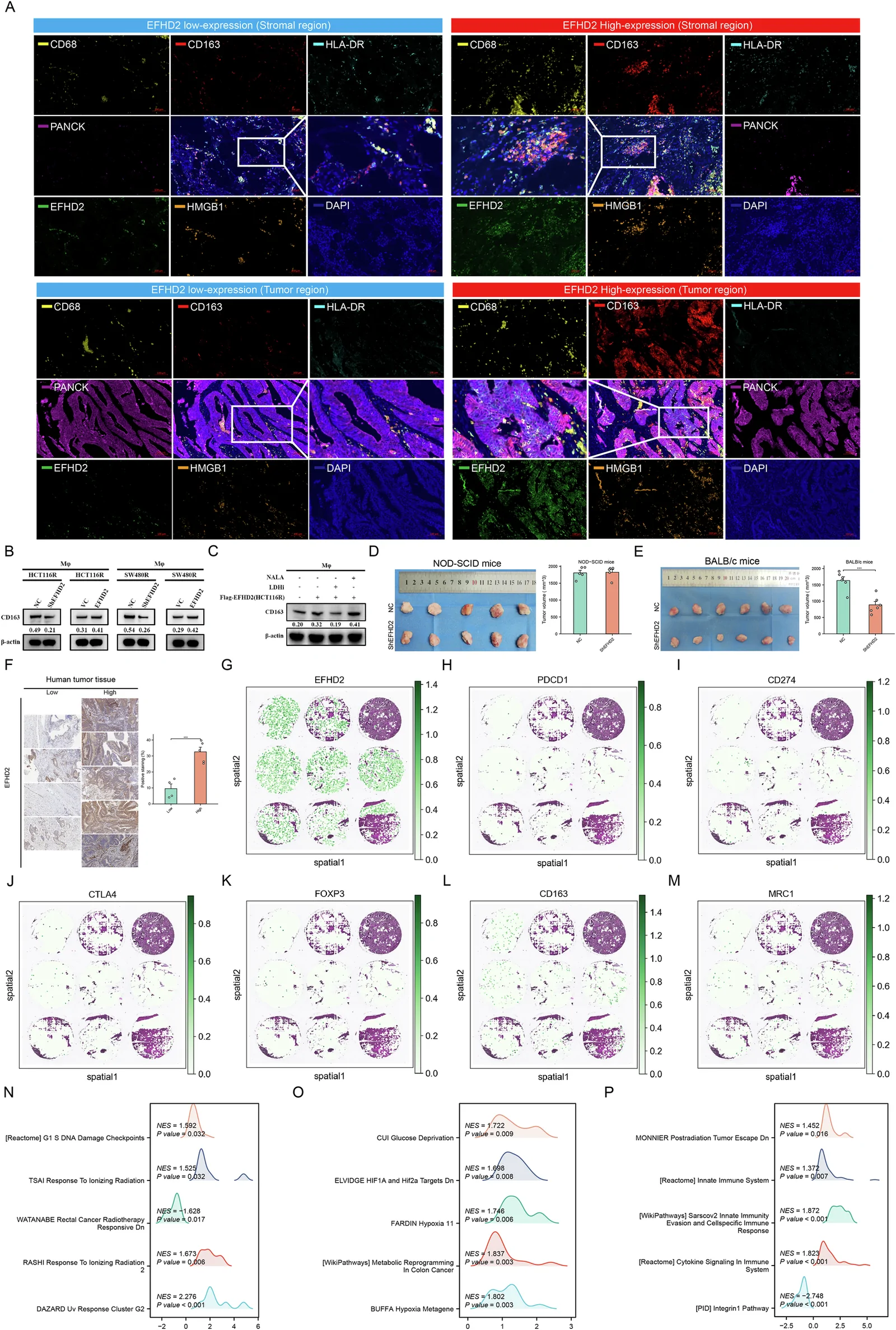
EFHD2 modulates macrophage polarization and shapes the spatial immune landscape. **A** Multiplex immunohistochemistry analysis showing that EFHD2 expression in the tumor parenchyma and stromal regions positively correlates with HMGB1. EFHD2 expression is associated with both M1 (CD68⁺HLA-DR⁺) and M2 (CD68⁺CD163⁺) macrophage markers in the stroma, but only with M2 markers in the tumor parenchyma. **B** Conditioned medium derived from EFHD2-modulated cells influenced CD163 expression in co-cultured macrophages; NALA promoted M2 polarization, while LDHi suppressed it. **C** Lactate metabolism modulation affects M2 polarization, with NALA promoting and the LDH inhibitor (LDHi) suppressing CD163 expression. **D**, **E** In vivo tumor growth assays showing that EFHD2 knockdown fails to suppress tumor growth in immunodeficient mice but significantly inhibits tumor growth in immunocompetent mice. **F** Immunohistochemistry analysis of nine human rectal cancer samples classified into EFHD2-high (*n* = 5) and EFHD2-low (*n* = 4) groups. **G**–**K** EFHD2 expression positively correlates with immune checkpoint markers, including PDCD1, CD274, CTLA4, and FOXP3. **L**, **M** EFHD2 expression positively correlates with M2 macrophage-related markers (CD163 and MRC1). **N**–**P** Gene set enrichment analysis of differentially expressed genes between EFHD2-high and EFHD2-low samples shows enrichment in pathways related to the ionizing radiation response, HIF-1α and lactate metabolism, and immune suppression.

Based on single-cell analysis showing that EFHD2 was associated with macrophage polarization, we performed multiplex immunohistochemistry experiments to further investigate this relationship. EFHD2 expression was positively correlated with HMGB1 in both the tumor parenchyma and stroma. Interestingly, EFHD2 was positively associated with both M1 (CD68⁺HLA-DR⁺) and M2 (CD68⁺CD163⁺) macrophage markers in the stroma, but only with M2 markers in the tumor parenchyma (Fig. 6A; Supplementary Fig. 9A–R). This discrepancy may be due to metabolic differences between compartments, as the tumor parenchyma generates more lactate than the stroma, which preferentially promotes M2 polarization. Public GEO datasets also confirmed a positive correlation between EFHD2 and M2 polarization (Supplementary Fig. 9S, T). These findings led us to hypothesize that EFHD2 may influence macrophage polarization through lactate metabolism. Conditioned medium derived from EFHD2-knockdown or EFHD2-overexpression cells modulated CD163 levels in co-cultured macrophages, with reduced CD163 expression in the knockdown group and increased levels in the overexpression group (Fig. 6B). Finally, we found that NALA promoted M2 macrophage polarization, whereas LDHi suppressed it (Fig. 6C; Supplementary Fig. 10A), further supporting a role of lactate metabolism in EFHD2-mediated macrophage polarization.

### EFHD2 shapes spatial immune landscapes and promotes immunosuppression and therapy resistance

Based on single-cell and mIHC analyses indicating a potential role of EFHD2 in modulating the tumor immune microenvironment, we conducted in vivo experiments using both immunodeficient and immunocompetent mice to further investigate the role of EFHD2 in immune regulation. EFHD2 knockdown failed to suppress tumor growth in immunodeficient mice but significantly inhibited tumor growth in immunocompetent mice (Fig. 6D, E), indicating that the antitumor effect of EFHD2 knockdown may be immune-dependent. Further immune infiltration analysis revealed that EFHD2-high tumors exhibited increased Treg infiltration but reduced B cell, dendritic cell, CD4⁺, and CD8⁺ T cell levels, along with lower immune scores compared to EFHD2-low tumors (Supplementary Fig. 10B–I). To comprehensively understand how EFHD2 expression spatially correlates with immune landscapes within tumors, we performed spatial transcriptomic analysis on nine human rectal cancer tissue samples (Supplementary Fig. 11A), categorized into EFHD2-high (*n* = 5) and EFHD2-low (*n* = 4) groups based on immunohistochemistry (Fig. 6F). Quality control and filtering confirmed robust data quality (Supplementary Fig. 11B). Spatial analysis revealed that EFHD2 expression was positively correlated with immune checkpoint markers (PDCD1, CD274, CTLA4, FOXP3) (Fig. 6G–K), and with M2 macrophage-related markers (CD163, MRC1, MSR1, PPARG, STAT6, TGFB1, CCL18, CCL22, CCL24) (Fig. 6L, M; Supplementary Fig. 11C–I). GSEA of differentially expressed genes between EFHD2-high and EFHD2-low samples showed enrichment of pathways related to ionizing radiation response (Fig. 6N), HIF-1α and lactate metabolism (Fig. 6O), immune suppression (Fig. 6P), DNA replication (chemoresistance, Supplementary Fig. 11J), and NF-κB signaling (Supplementary Fig. 11K). These findings further support the functional role of EFHD2 in promoting an immunosuppressive and therapy-resistant tumor phenotype.

### EFHD2 shapes the tumor immune microenvironment

**Fig. 7:**
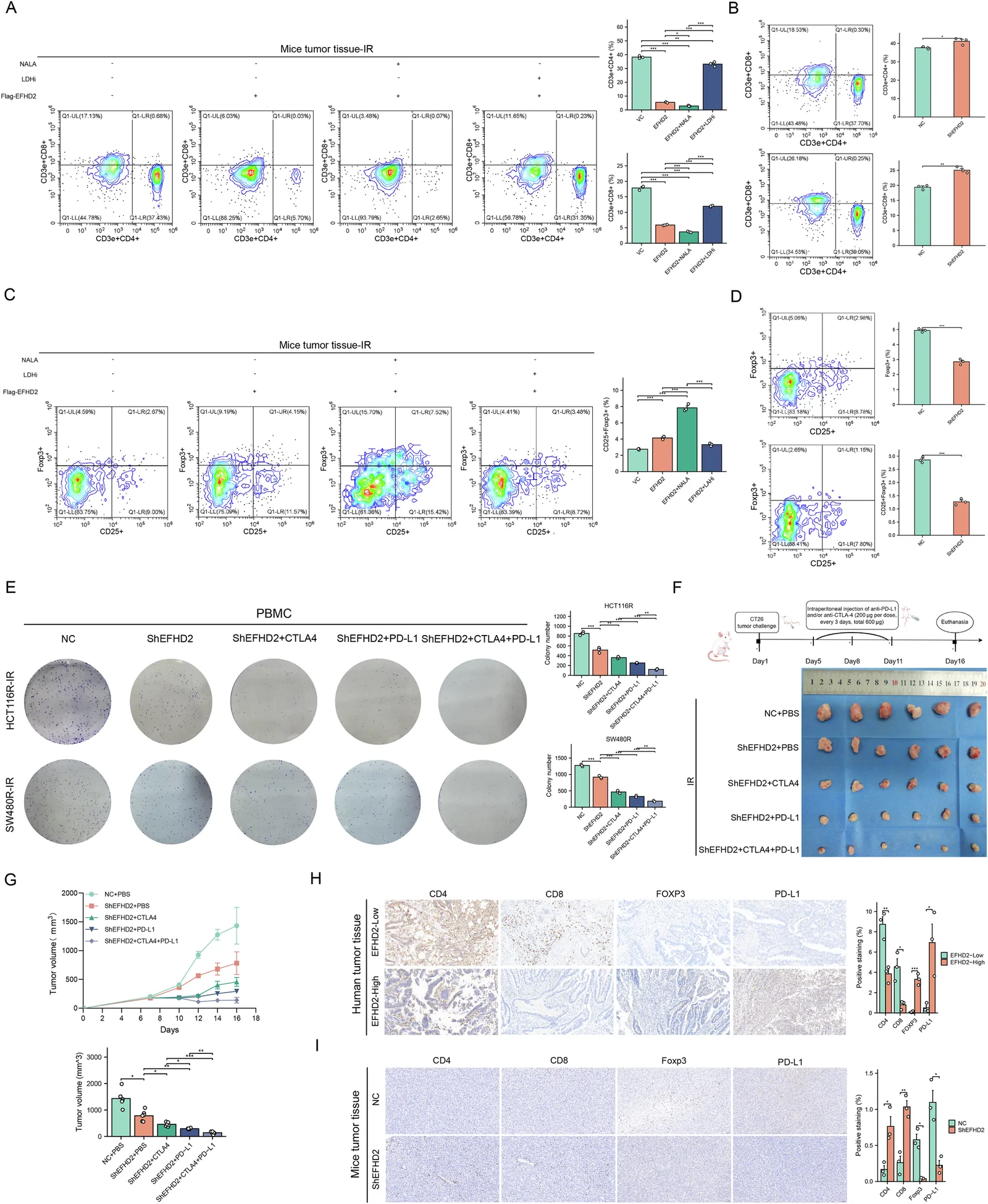
EFHD2 shapes the tumor immune microenvironment and modulates the immunotherapy response. **A**, **B** Flow cytometry analysis showing that EFHD2 overexpression decreases the proportions of CD4⁺ and CD8⁺ T cells while increasing the proportion of Foxp3⁺ Tregs. NALA treatment reduces the proportions of CD4⁺ and CD8⁺ T cells and increases the proportion of Foxp3⁺ Tregs, whereas LDHi treatment has the opposite effect. **C**, **D** EFHD2 knockdown increases the proportion of CD4⁺ and CD8⁺ T cells and decreases the proportion of Foxp3⁺ Tregs relative to NC cells. **E** Colony formation assays under PBMC co-culture conditions demonstrate that EFHD2 knockdown combined with anti-PD-L1 or anti-CTLA-4 therapy following irradiation significantly inhibits tumor cell proliferation. **F**, **G** In vivo tumor growth assays confirm that EFHD2 knockdown enhances the antitumor effects of immune checkpoint blockade therapy. **H**, **I** Immunohistochemistry analysis of human rectal cancer tissues and murine tumors shows that high EFHD2 expression is associated with reduced CD4⁺ and CD8⁺ T cell infiltration and increased FOXP3⁺ and PD-L1 expression.

To investigate the impact of EFHD2 on the immune microenvironment, we performed flow cytometry analysis on tumor tissues. Compared to the control group, EFHD2 overexpression decreased the proportions of CD4⁺ and CD8⁺ T cells while increasing the proportion of Foxp3⁺ Tregs. NALA treatment reduced the proportions of CD4⁺ and CD8⁺ T cells and increased the proportion of Foxp3⁺ Tregs, whereas LDHi had the opposite effect (Fig. 7A, B). In addition, EFHD2 knockdown resulted in a higher proportion of CD4⁺ and CD8⁺ T cells and a lower proportion of Foxp3⁺ Tregs relative to the NC group (Fig. 7C, D). Colony formation assays in a PBMC co-culture system revealed that EFHD2 knockdown combined with anti-PD-L1 or anti-CTLA-4 therapy following irradiation significantly inhibited tumor cell proliferation (Fig. 7E), which was confirmed in vivo (Fig. 7F, G). Immunohistochemistry of both human rectal cancer tissues and murine tumors showed that high EFHD2 expression was associated with lower CD4⁺ and CD8⁺ T cell infiltration and higher FOXP3⁺ and PD-L1 expression (Fig. 7H, I; Supplementary Fig. 11L, M).

### Potential role of EFHD2 in modulating radiotherapy resistance via NF-κB activation and PD-L1 upregulation

**Fig. 8:**
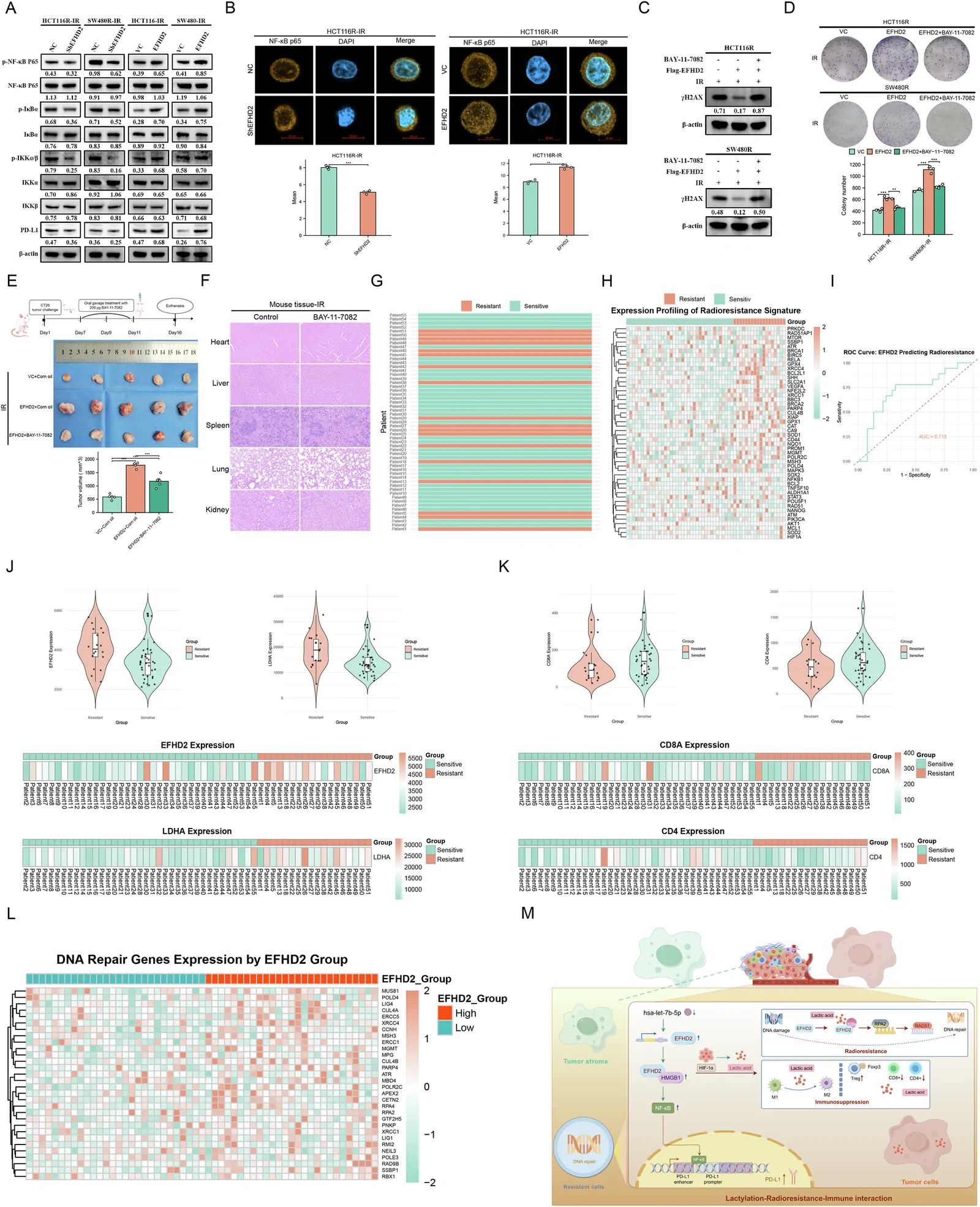
EFHD2 modulates the tumor immune microenvironment and NF-κB/PD-L1 signaling to enhance radioresistance. **A** Western blot analysis showing that EFHD2 knockdown suppresses phosphorylation of IKKα/β, IκBα, and NF-κB p65, as well as PD-L1 expression in radioresistant cells following irradiation, whereas EFHD2 overexpression enhances their phosphorylation and PD-L1 expression. **B** Immunofluorescence analysis confirming that EFHD2 promotes NF-κB p65 nuclear translocation under radiotherapy conditions. **C**–**E** Treatment with the NF-κB inhibitor BAY-11-7082 reverses the EFHD2-induced reduction of γH2AX levels and radioresistance in vitro and in vivo. **F** BAY-11-7082 does not induce detectable organ toxicity in treated mice. **G** Transcriptomic analysis of 18 radioresistant and 37 radiosensitive rectal cancer samples collected from our center. **H** Heatmap showing that genes related to DNA damage repair, apoptosis, stemness, oxidative stress, hypoxia, the tumor microenvironment, and signaling pathways are generally upregulated in the radioresistant group. **I** ROC curve analysis demonstrating that EFHD2 predicts radioresistance with an AUC of 0.713. **J**, **K** EFHD2 and LDHA are elevated in radioresistant tumors, whereas CD8A and CD4 expression is lower. **L** High EFHD2 expression correlates with increased expression of DNA damage repair-related genes. **M** Schematic model summarizing EFHD2-mediated regulation of macrophage polarization, lactate metabolism, NF-κB signaling, DNA damage repair, and immunosuppression in radioresistance.

Given the enriched NF-κB signaling, we aimed to explore its role in regulating radiosensitivity. EFHD2 knockdown suppressed phosphorylation of IKKα/β, IκBα, and NF-κB-p65, as well as PD-L1 expression, in resistant cells following irradiation, while EFHD2 overexpression increased their phosphorylation and PD-L1 expression (Fig. 8A). Immunofluorescence confirmed that EFHD2 promoted NF-κB p65 nuclear translocation (Fig. 8B; Supplementary Fig. 11N, O), demonstrating that EFHD2-mediated activation of NF-κB is enhanced under radiotherapy conditions, thereby contributing to radiotherapy resistance. BAY-11-7082, an NF-κB inhibitor, reversed the EFHD2-induced reduction of γH2AX levels and radioresistance both in vitro (Fig. 8C, D) and in vivo (Fig. 8E), without inducing organ toxicity (Fig. 8F).

### EFHD2 expression correlates with radioresistance, lactate metabolism, and immune modulation in clinical rectal cancer samples

To further validate the impact of EFHD2 on radioresistance, lactate metabolism, and the tumor immune microenvironment, we analyzed transcriptomic data from 18 radioresistant and 37 radiosensitive clinical rectal cancer samples collected from our center (Fig. 8G). A heatmap depicting the expression profiles of genes related to DNA damage repair, apoptosis, stemness, oxidative stress, hypoxia, the tumor microenvironment, and signaling pathways revealed that these genes were generally upregulated in the radioresistant group compared to the radiosensitive group (Fig. 8H). ROC curve analysis demonstrated that EFHD2 predicted radioresistance with an AUC of 0.713, indicating a significant predictive value (Fig. 8I). Analysis of EFHD2, LDHA, CD8A, and CD4 expression between the radioresistant and radiosensitive groups showed that EFHD2 and LDHA were significantly elevated in radioresistant tumors, whereas CD8A and CD4 expression was lower in these samples (Fig. 8J, K). Similarly, expression levels of PKM and HK1 were higher in the radioresistant group (Supplementary Fig. 12A–D). Correlation analysis indicated that EFHD2 expression was positively associated with LDHA, PKM, and HK1, and negatively associated with CD8A and CD4 (Supplementary Fig. 12E). Further analysis of EFHD2 expression in relation to DNA damage repair and immune-related genes revealed that high EFHD2 expression was associated with elevated expression of DNA repair-related genes (Fig. 8L) but decreased expression of immune-related genes (Supplementary Fig. 12F). Gene enrichment analysis of differentially expressed genes between EFHD2 high and EFHD2-low expression groups demonstrated that EFHD2 was closely associated with functions related to double-strand break repair, DNA recombination, homologous recombination repair, and DNA replication (Supplementary Fig. 12G). Finally, the proposed mechanism underlying our findings was illustrated in Fig. 8M.

## Discussion

This study uncovers a novel regulatory axis involving hsa-let-7b-5p, EFHD2, and lactate metabolism that governs both intrinsic and extrinsic mechanisms of radioresistance in CRC. We demonstrate that hsa-let-7b-5p enhances radiosensitivity by suppressing EFHD2 expression, thereby impairing HR repair efficiency and disrupting the metabolic reprogramming that sustains an immunosuppressive tumor microenvironment. This dual regulatory function places hsa-let-7b-5p at the intersection of genome stability and immune modulation, a finding that adds to the growing recognition of microRNAs as key integrators of the DNA damage response and tumor immunity [18].

At the mechanistic level, we provide evidence that EFHD2 promotes HR repair by facilitating the recruitment of critical DNA repair factors, including RPA2 and RAD51. EFHD2 knockdown resulted in decreased formation of RPA2 and RAD51 nuclear foci following irradiation, suggesting its essential role in maintaining HR fidelity. Sustained γH2AX levels in EFHD2-deficient cells further support the presence of unrepaired double-strand breaks, corroborating the loss of efficient HR-mediated repair. These findings are consistent with prior reports that link HR factor availability and chromatin dynamics to the radiation response [19], and they extend current understanding by positioning EFHD2 as a scaffold-like regulator of DNA repair assembly.

A central insight from our study is the identification of EFHD2 lactylation at lysine 9 (K9) as a post-translational modification that governs HR activity. Given that EFHD2 contains calcium-binding EF-hand motifs, we speculate that lactylation at K9—a site proximal to the EF-hand domain—may influence its structural conformation, calcium-binding capacity, or protein-protein interactions essential for DNA repair complex assembly. Supporting this, inhibition of lactate production via LDH blockade mimicked EFHD2 loss-of-function by reducing RAD51 foci and sensitizing cells to radiation. Our findings highlight a mechanistic link between cellular metabolism and DNA repair capacity, where lactate serves not merely as a metabolic byproduct but as a signaling molecule that enhances homologous recombination fidelity. This concept builds on previous observations connecting tumor glycolysis to genome stability and expands them by identifying EFHD2 as a downstream effector of this metabolic control [20, 21], with our data further establishing EFHD2 as a functional effector of lactate-enhanced DNA repair fidelity.

In parallel with its DNA repair role, EFHD2 modulates the immune microenvironment via interaction with HMGB1, which activates the NF-κB signaling cascade. This axis promotes transcriptional programs associated with immune evasion, including PD-L1 upregulation and immunosuppressive cytokine release, as demonstrated in previous studies highlighting EFHD2’s role in tumor immune escape [22, 23]. Spatial transcriptomic analysis further revealed that EFHD2-expressing tumor regions were enriched for immune checkpoint markers such as PD-1, CTLA-4, and signatures indicative of M2 macrophage polarization, which are known to contribute to tumor progression and resistance to therapy [24, 25]. These findings are consistent with an immunoregulatory phenotype that facilitates immune evasion and supports tumor survival. Importantly, these spatially distinct effects suggest that EFHD2 orchestrates a multicellular network favoring radiotherapy resistance through both paracrine and autocrine mechanisms, potentially by modulating crosstalk between tumor cells and the immune stroma. Such complex interactions underscore the therapeutic potential of targeting EFHD2-related pathways to enhance radiotherapy efficacy and overcome immune suppression.

The impact of EFHD2 on macrophage polarization appears to be metabolically driven. EFHD2 upregulates HIF-1α in an HMGB1-dependent manner, leading to increased lactate production and secretion. Lactate, in turn, reinforces the M2-like polarization of tumor-associated macrophages (TAMs), as evidenced by increased CD163 expression in co-culture and multiplex immunohistochemistry. These findings are consistent with emerging literature demonstrating that tumor-derived lactate shapes immune cell function and contributes to therapeutic resistance [26, 27]. Our data provide the first evidence that EFHD2 serves as a metabolic and immunologic hub, promoting a feed-forward loop that stabilizes a radiation-resistant niche.

The observation that EFHD2 knockdown enhances radiosensitivity only in immunocompetent models underscores the dominant contribution of immune-mediated mechanisms in EFHD2-driven resistance. This distinction between intrinsic and immune-dependent radioresistance has practical significance, as it implies that EFHD2-targeted strategies may be optimized when combined with immune checkpoint blockade or myeloid cell modulators. The observed synergy between EFHD2 inhibition and anti-PD-L1 therapy in preclinical models highlights this translational potential and supports further development of rational combination therapies, especially in microsatellite-stable CRC, which typically shows limited response to immunotherapy alone.

Despite providing comprehensive mechanistic insights, our study has several limitations. First, the use of established CRC cell lines and xenograft models may not fully replicate the heterogeneity of primary human tumors, particularly regarding stromal architecture and immune cell composition. Second, while our spatial and single-cell transcriptomic analyses revealed key EFHD2-associated immunoregulatory features, they provide only correlative observations; functional validation of these findings in patient-derived organoids or ex vivo tissue systems remains necessary. Third, the lactyl-proteomic profiling utilized cannot distinguish between enzymatic and non-enzymatic lactylation, which may differ in their functional relevance. Fourth, although our study focused on homologous recombination repair, other DNA damage response pathways, such as non-homologous end joining and mismatch repair, may also contribute to EFHD2-mediated radioresistance and warrant further investigation. Finally, although our findings suggest that EFHD2 and protein lactylation may serve as potential biomarkers or therapeutic targets, their clinical applicability remains uncertain. Key factors such as assay standardization, inter-patient variability, and predictive value in the context of standard-of-care treatment have yet to be systematically evaluated.

## Conclusion

This study identifies a novel regulatory axis involving hsa-let-7b-5p, EFHD2, and lactate metabolism that underlies radioresistance in colorectal cancer. Mechanistically, EFHD2 interacts with HMGB1, enhancing lactate metabolism via HIF-1α stabilization. This lactate accumulation promotes homologous recombination repair by facilitating RAD51 and RPA2 recruitment, a process dependent on EFHD2 lysine 9 lactylation, and simultaneously fosters an immunosuppressive tumor microenvironment characterized by M2 macrophage polarization, Treg expansion, NF-κB activation, PD-L1 upregulation, and suppression of CD4⁺ and CD8⁺ T cell infiltration. Single-cell and spatial transcriptomic analyses, along with validation in clinical tumor samples, further reveal that EFHD2-positive immune cells adopt immunosuppressive and exhausted phenotypes. Collectively, these findings establish EFHD2 as a central orchestrator linking DNA repair, metabolism, and immune evasion, and suggest that targeting EFHD2 may overcome radioresistance and enhance the efficacy of immune checkpoint blockade in colorectal cancer.

## Supplementary information

Supplementary material

Original Data

Dataset 1

Dataset 2

Dataset 3

Dataset 4

Dataset 5.1

Dataset 5.2

Dataset 5.3

Dataset 5.4

Dataset 6

## Data Availability

All data associated with this study are presented in the article or the Supplementary Material and Data. Full and uncropped western blot images are provided in the Supplementary Data.
