# Supplementary material for: EFHD2 drives lactate-mediated DNA damage repair and immunosuppression via HMGB1 and HIF-1α to confer radioresistance in colorectal cancer
Source: Cell Death Dis. 2026 Jun 23;17(1):777. doi: 10.1038/s41419-026-09031-2 (PMC13542245; doi:10.1038/s41419-026-09031-2)
Supplement: Supplementary file 1 [file 41419_2026_9031_MOESM2_ESM.pdf]

Figure 1H

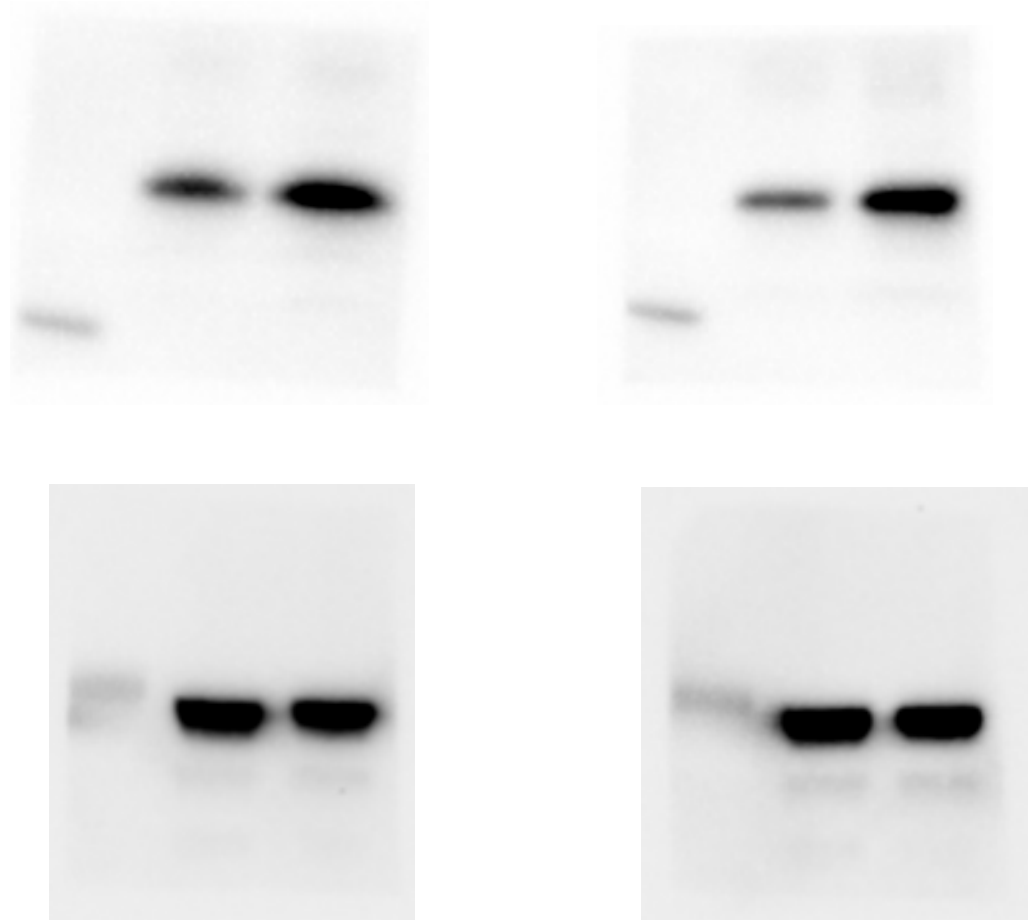

γH2AX

β-actin

Figure 1L

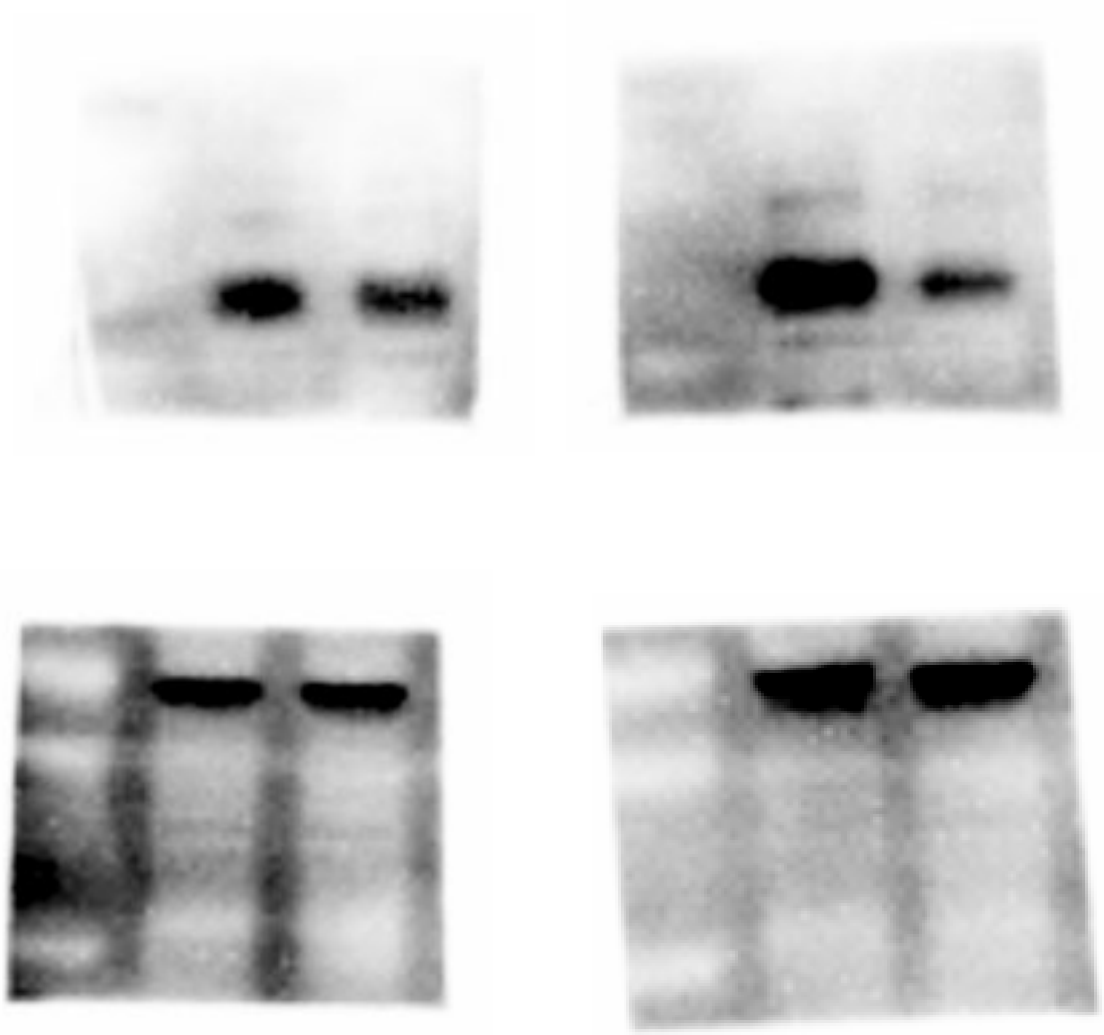

EFHD2

β-actin

Figure 1Q

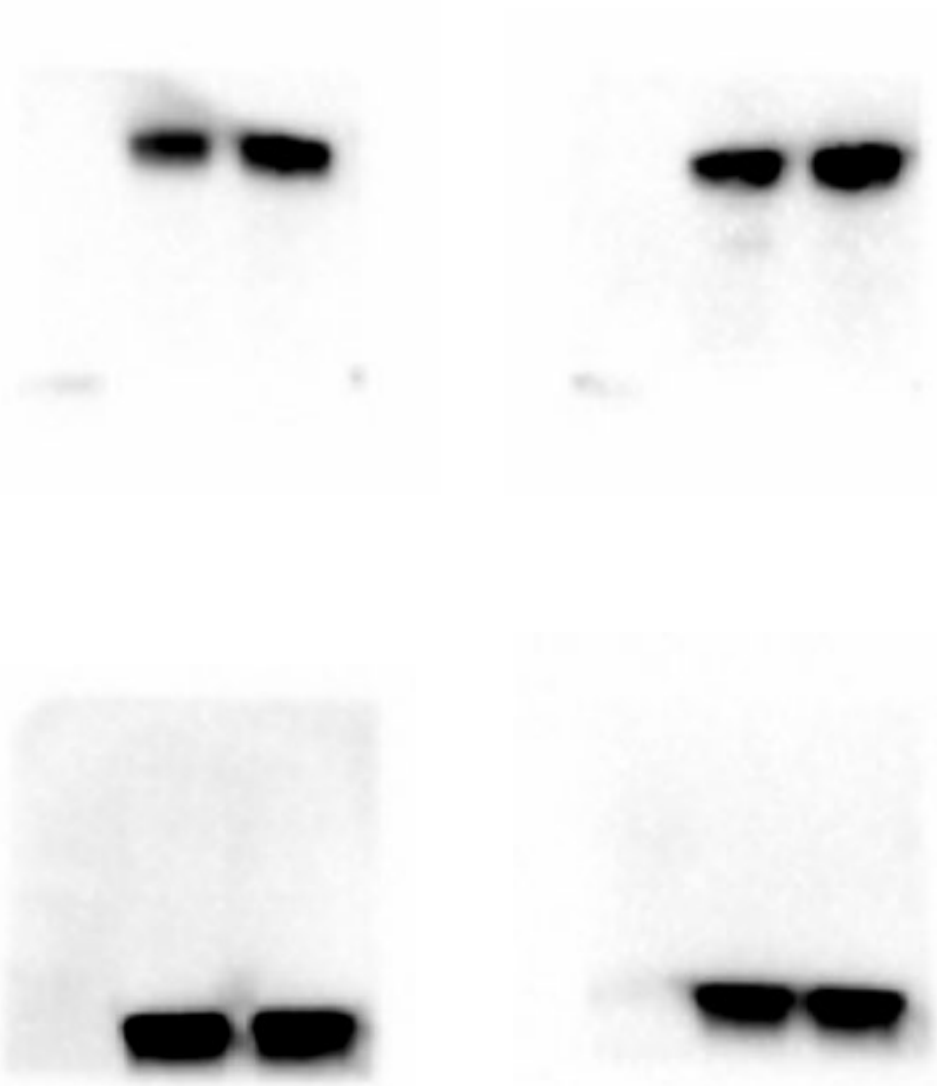

EFHD2

β-actin

Figure 1T

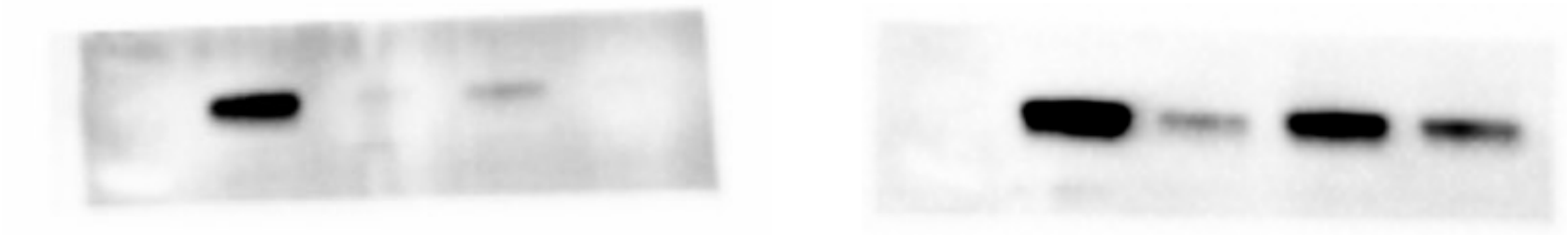

EFHD2

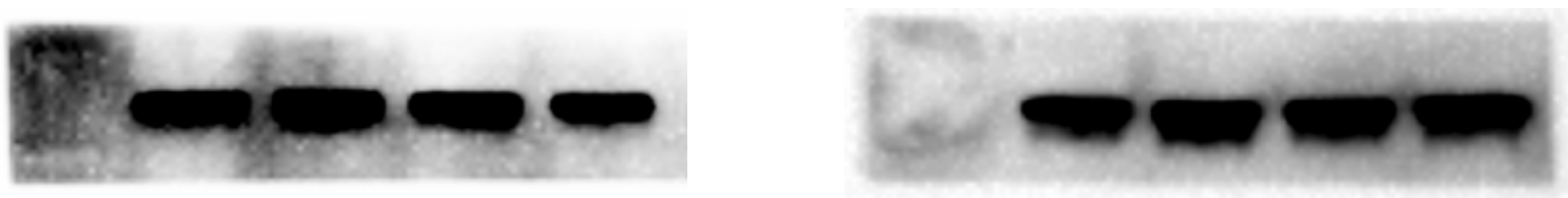

β-actin

Figure 1W

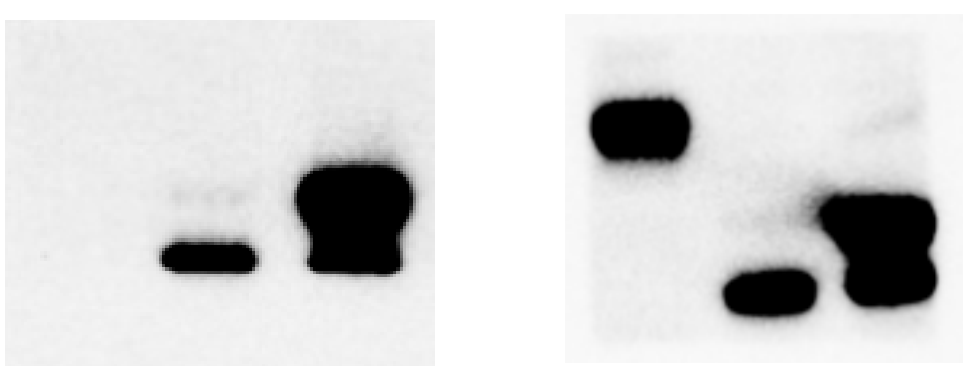

EFHD2

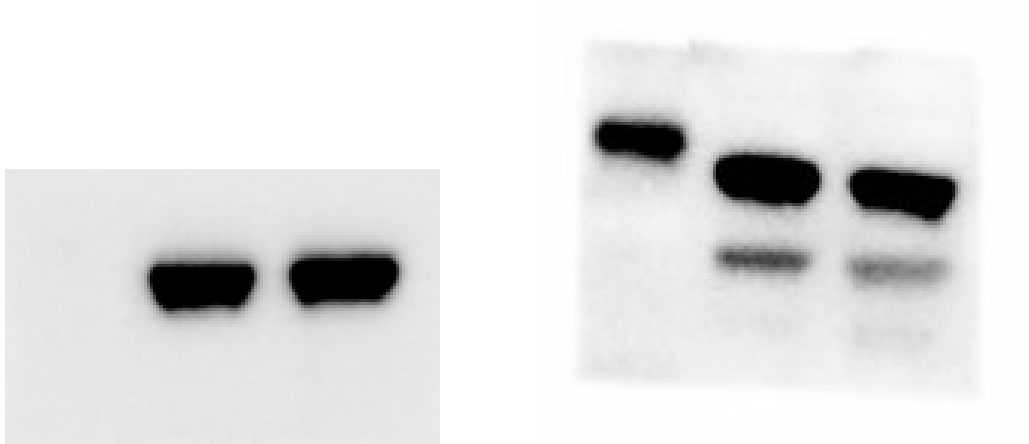

β-actin

Figure 2B

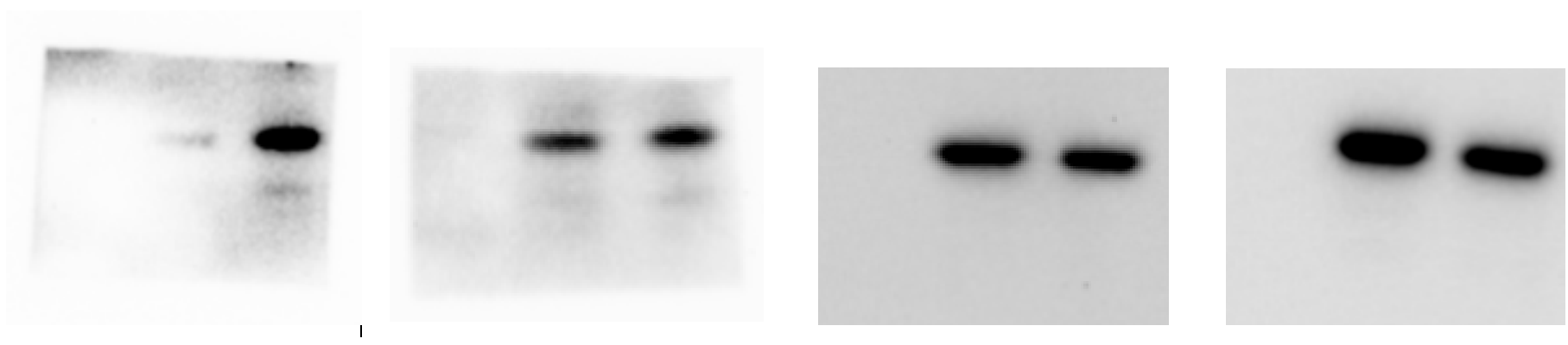

Bax

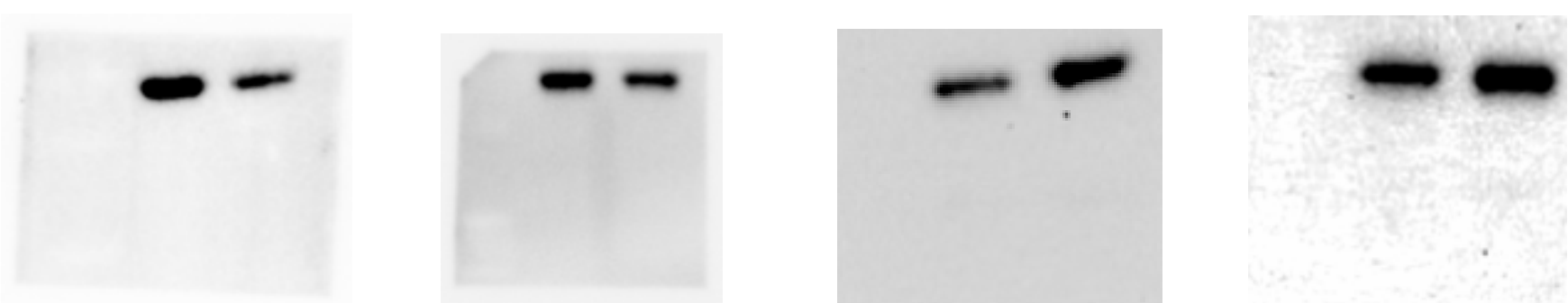

Bcl-2

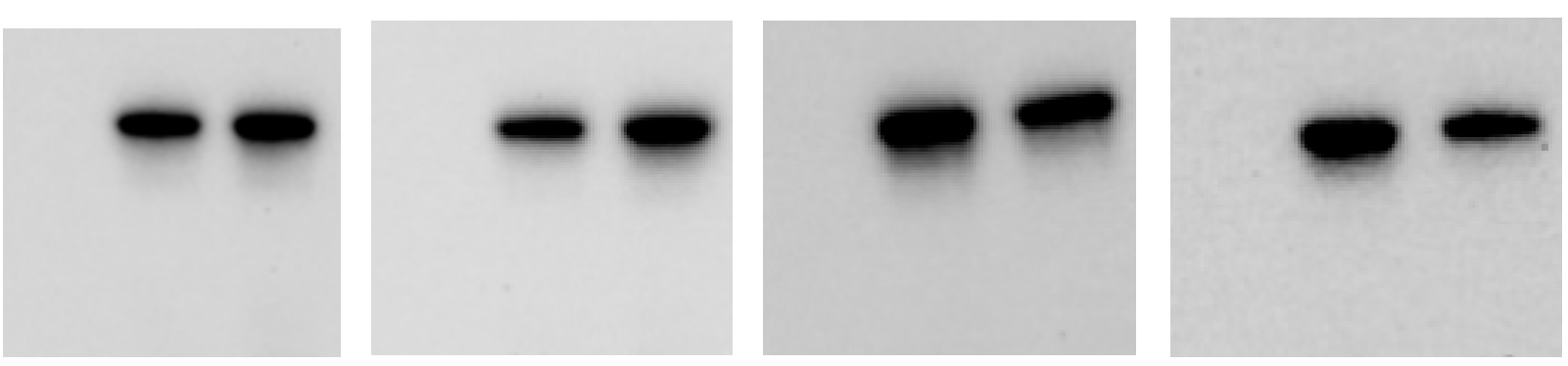

Caspase-3

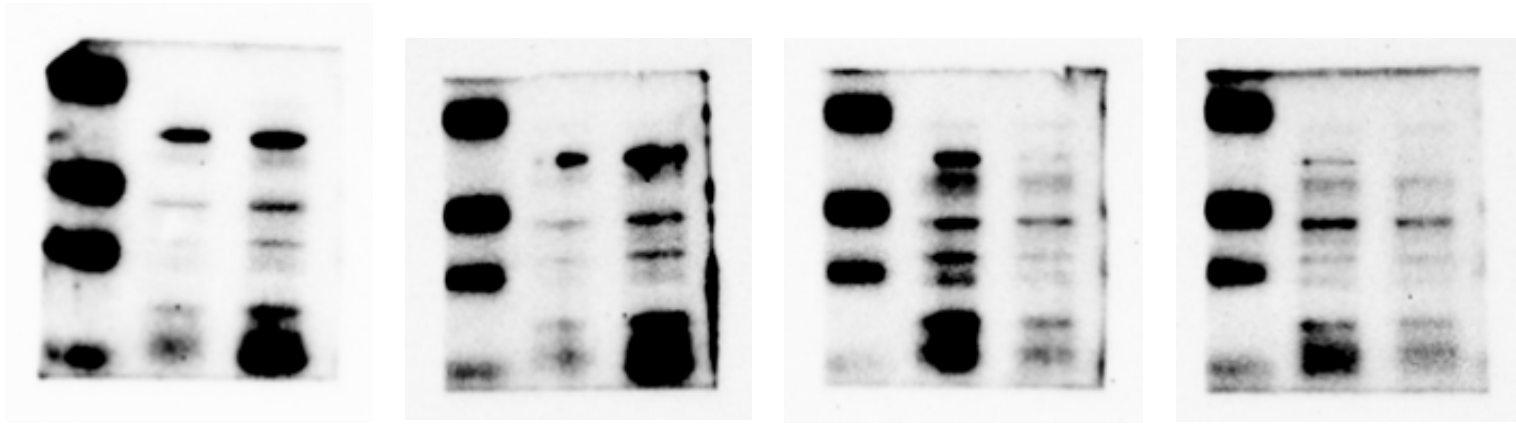

cleaved Caspase-3

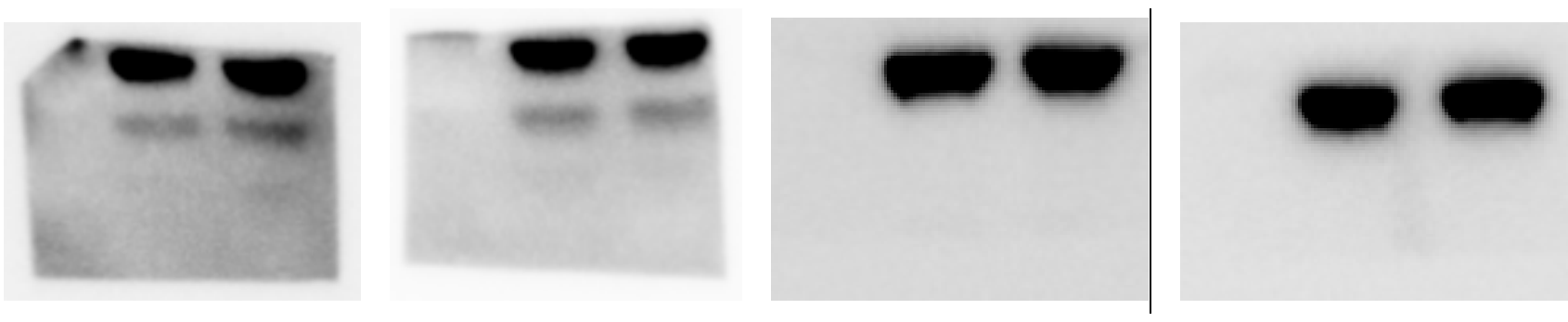

β-actin

Figure 2F

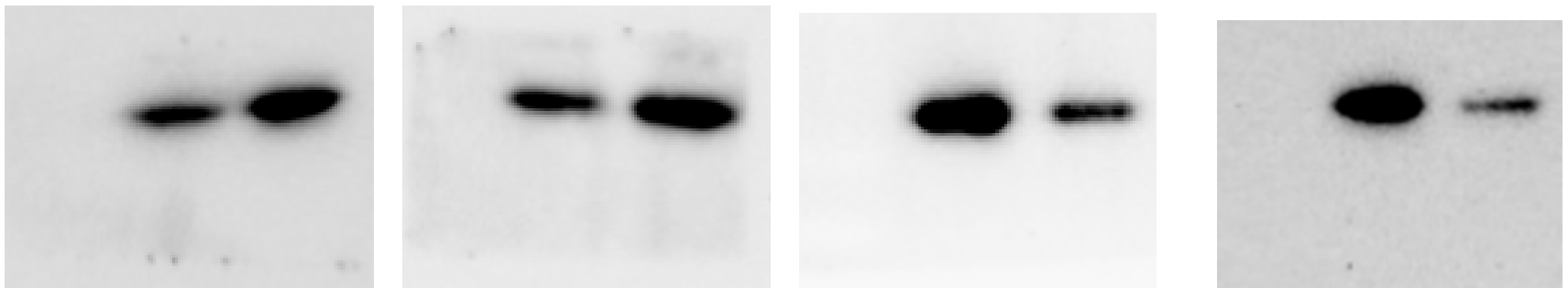

γH2AX

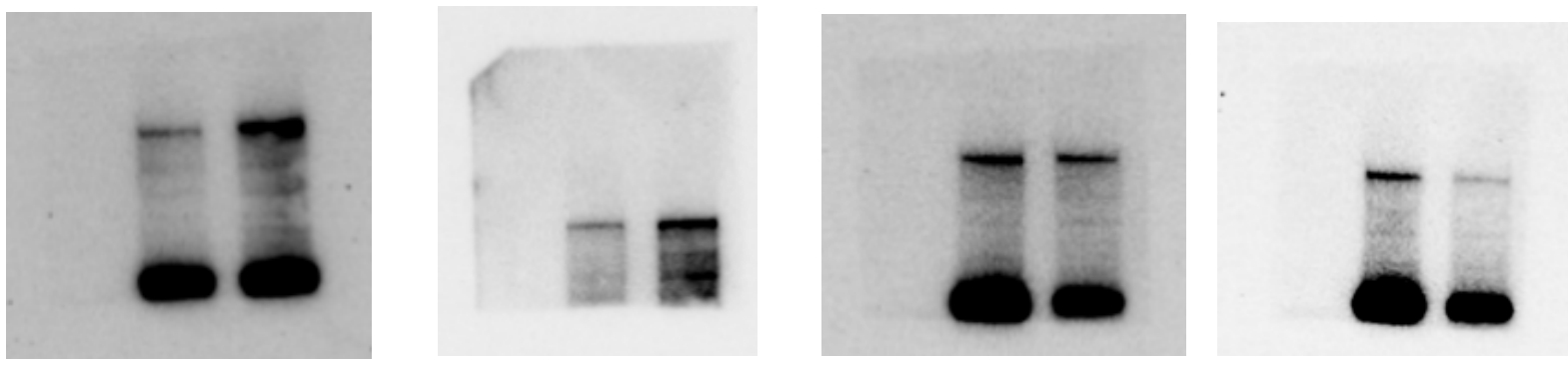

p-ATM

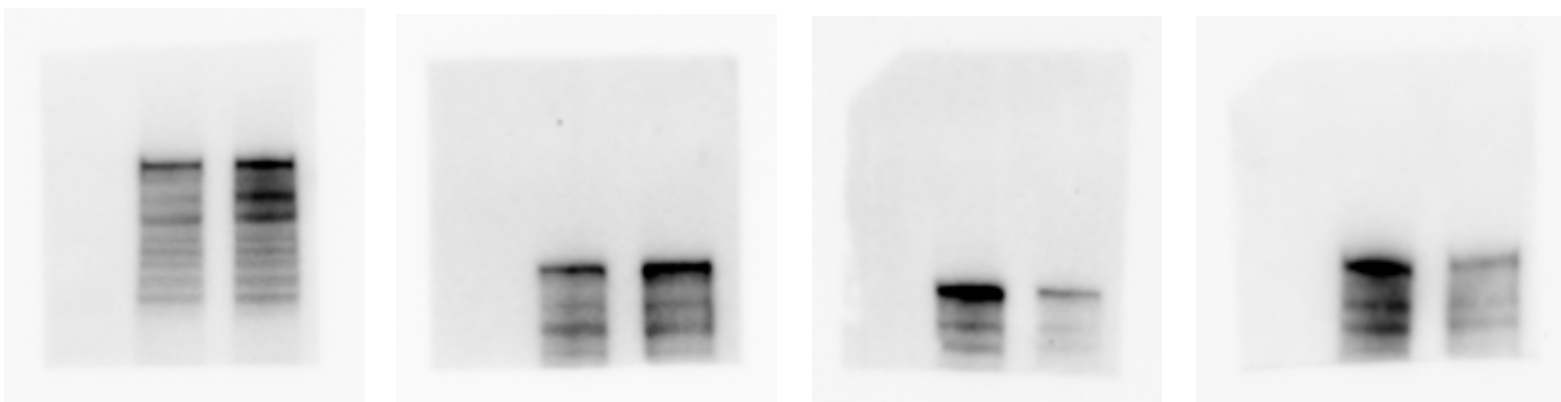

ATM

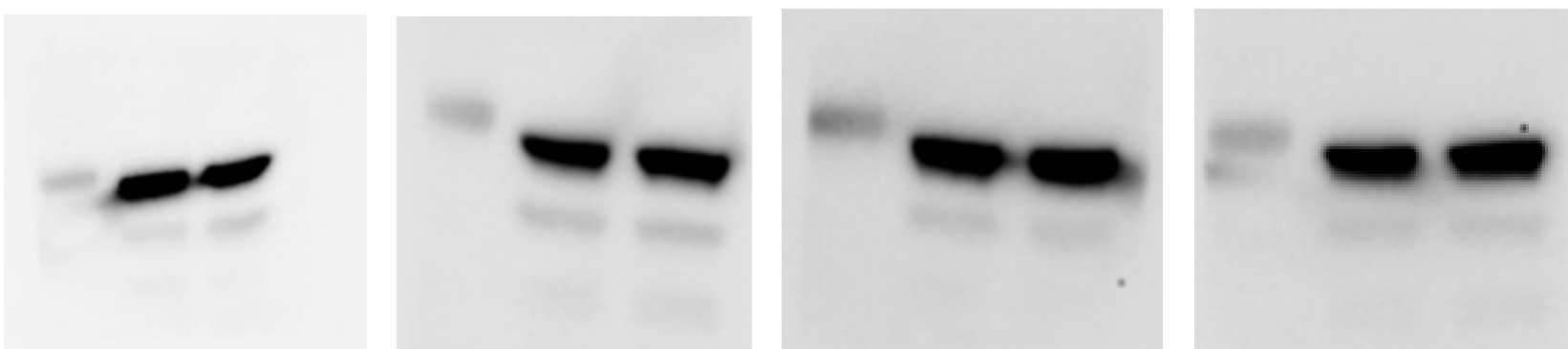

β-actin

Figure 2G

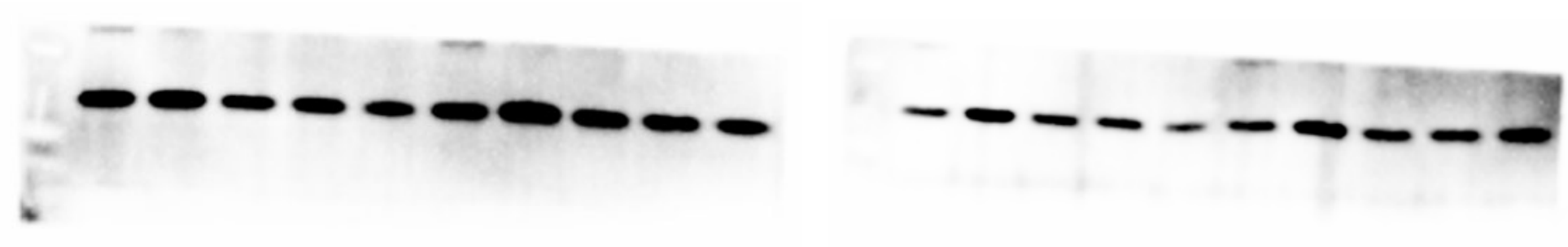

γH2AX

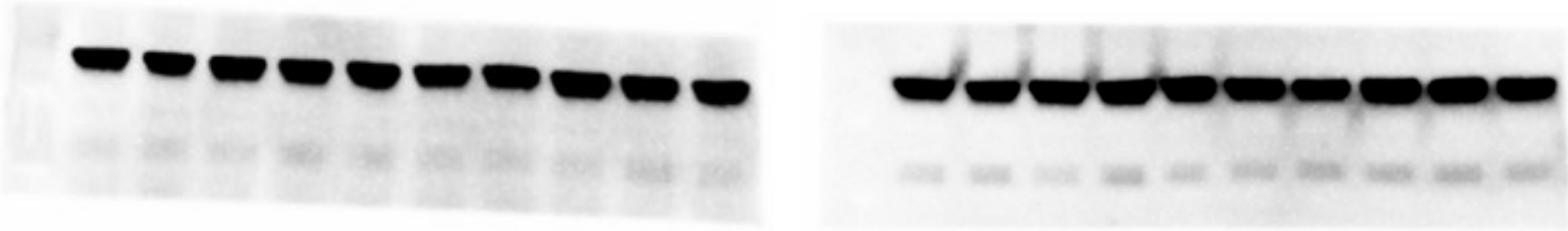

β-actin

Figure 3A

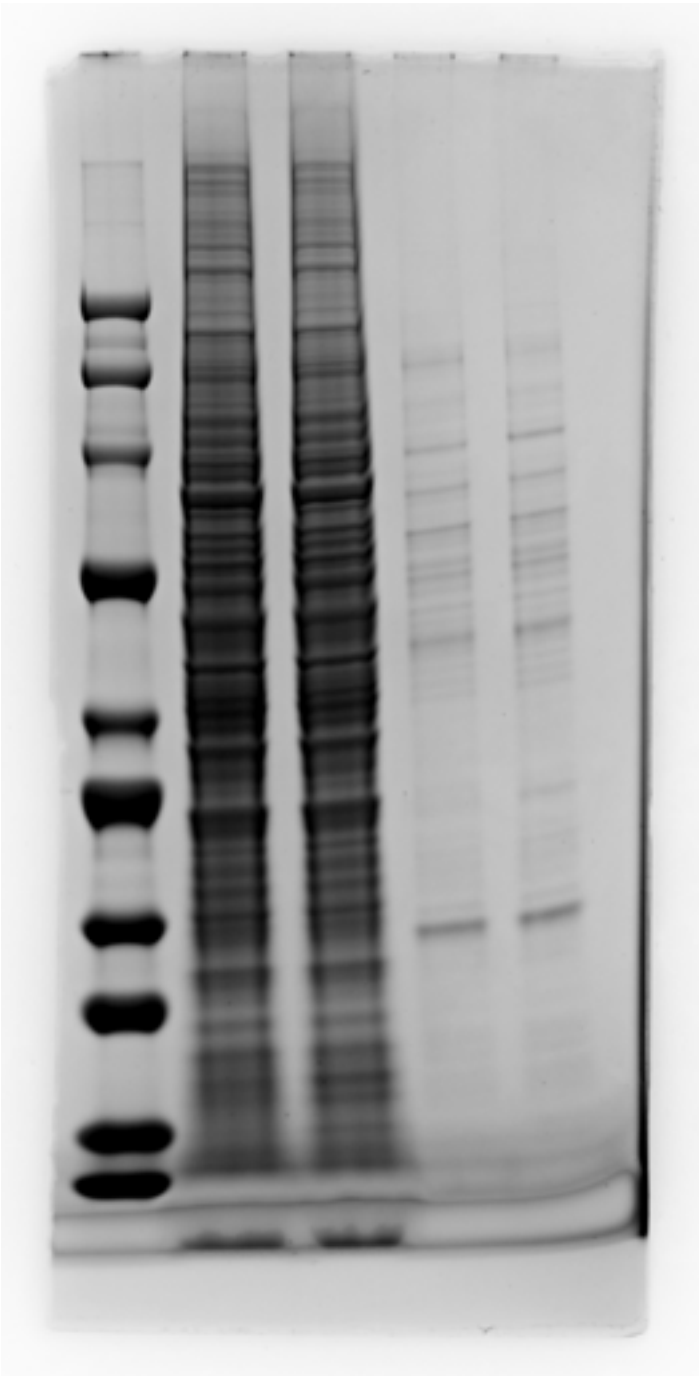

Figure 3C

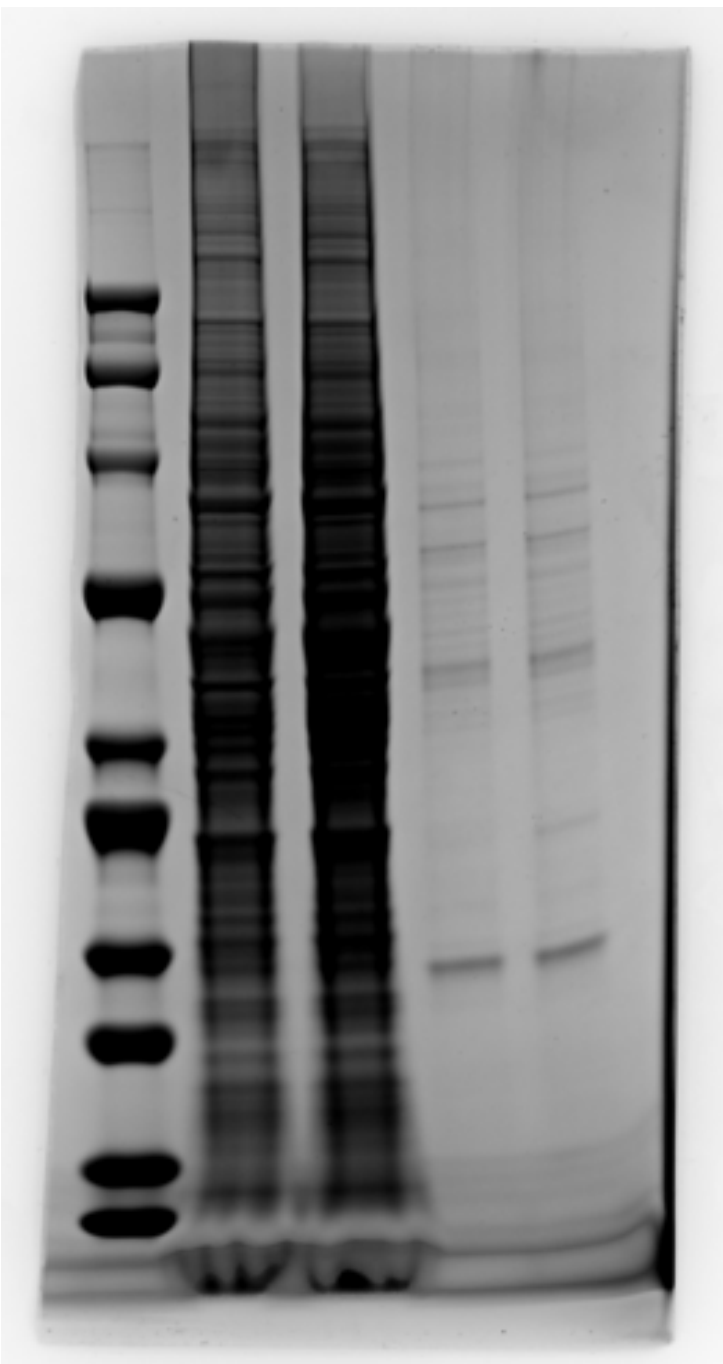

Figure 3D

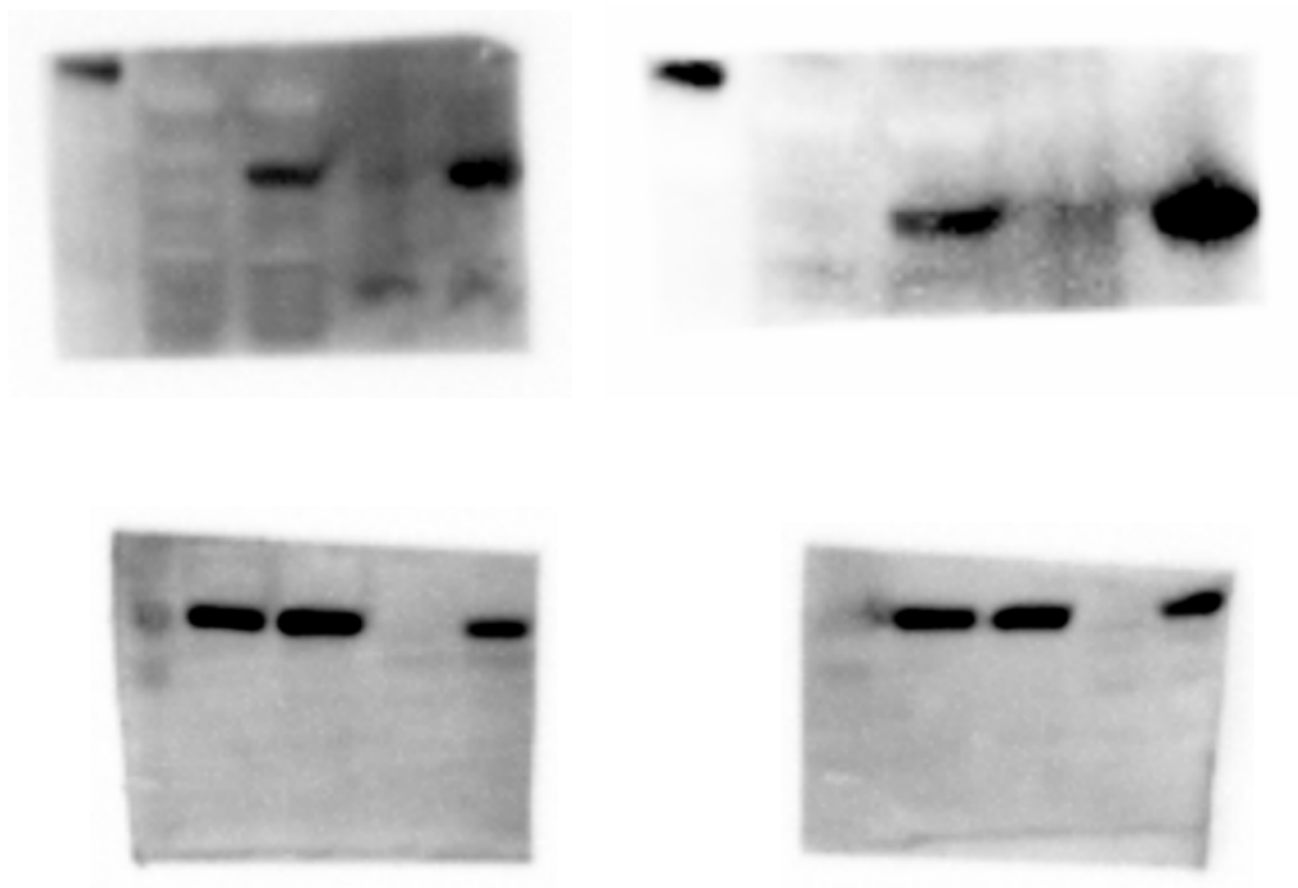

EFHD2-Flag

HMGB1

Figure 3F

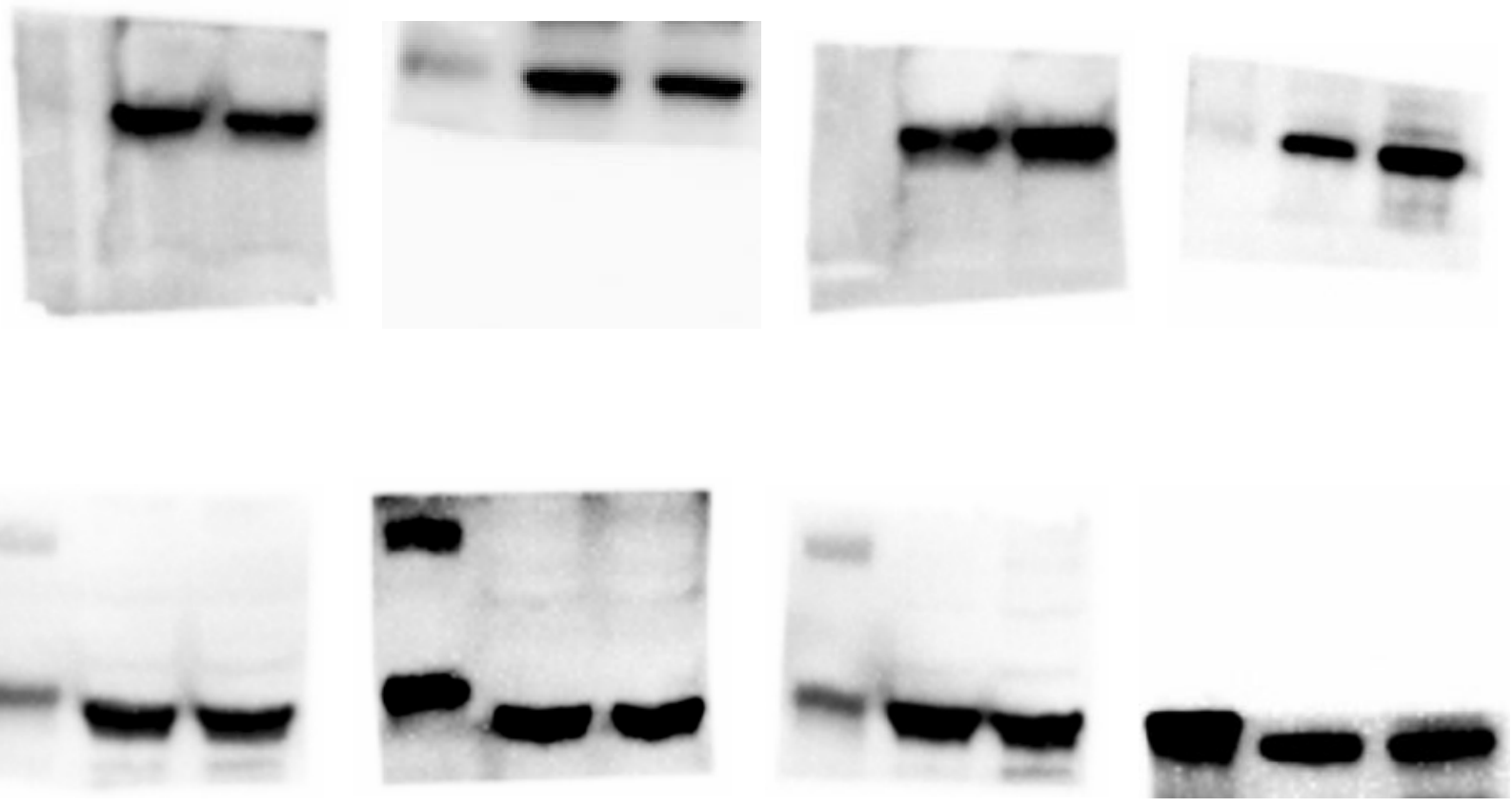

HMGB1

β-actin

Figure 3G

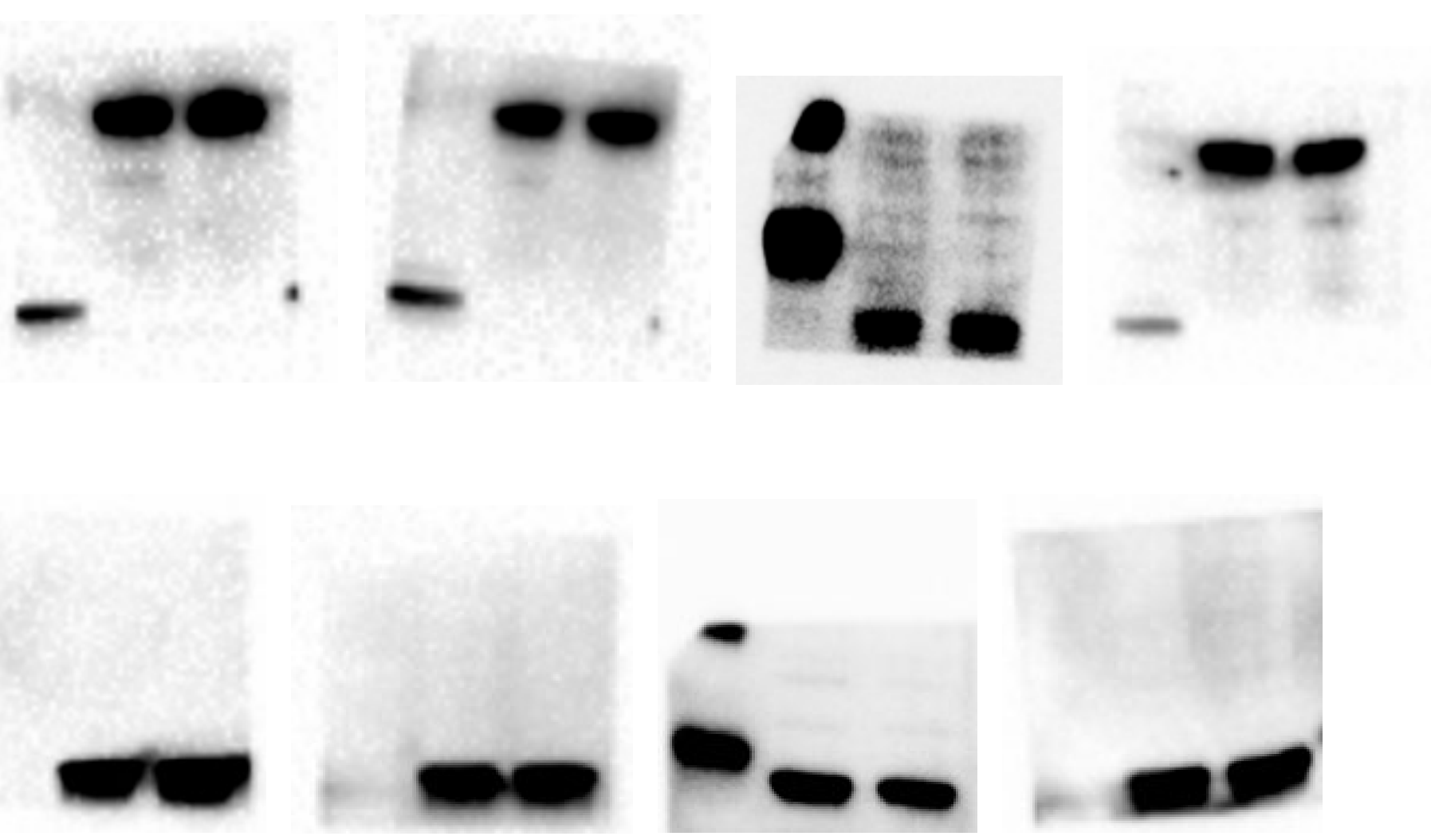

EFHD2

β-actin

Figure 3H

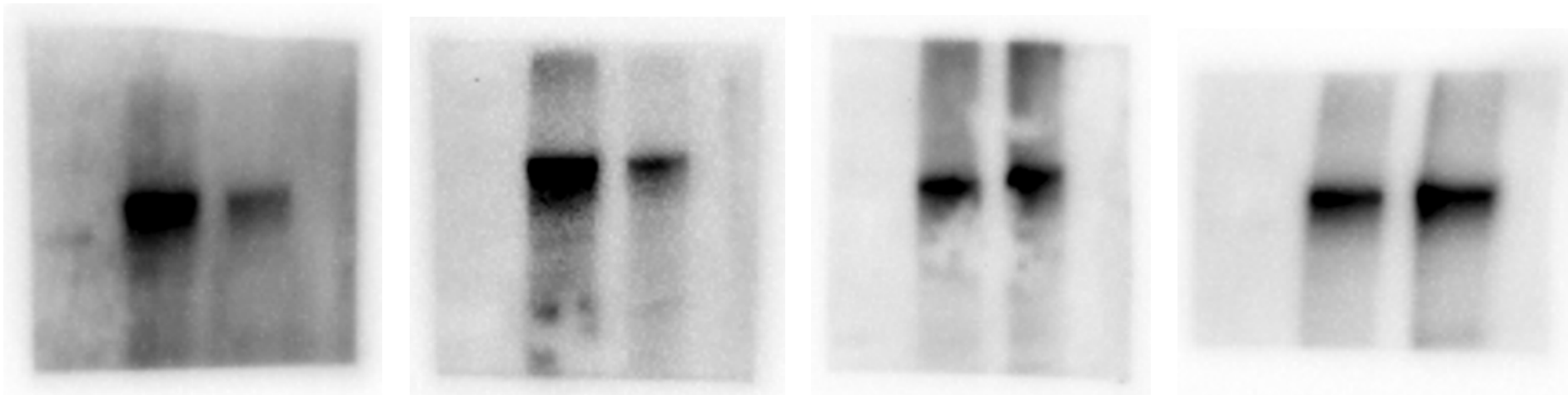

HIF-1α

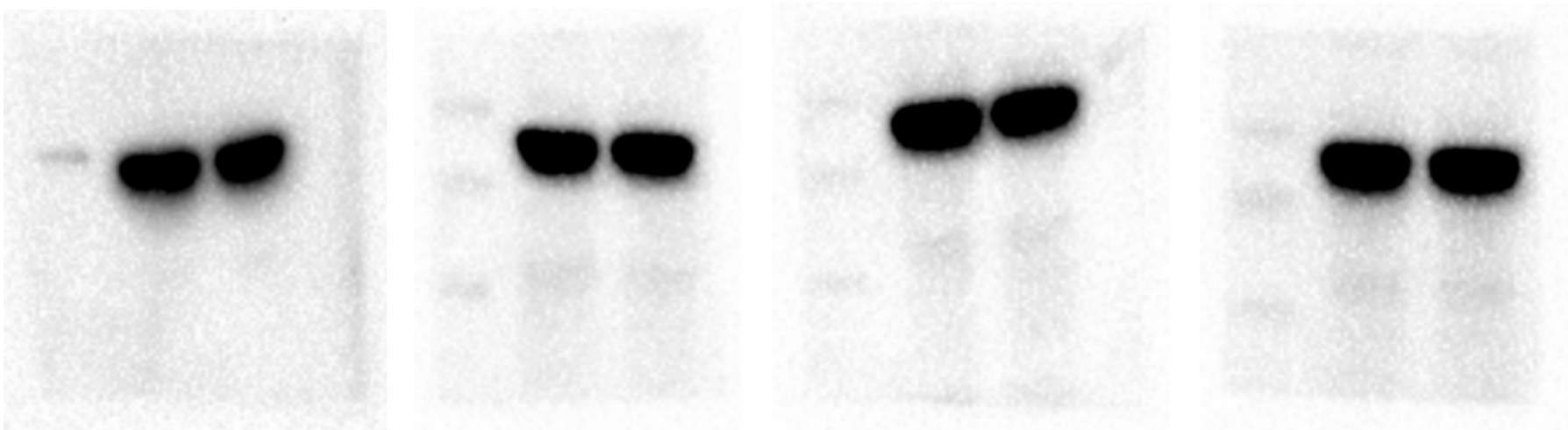

β-actin

Figure 3I

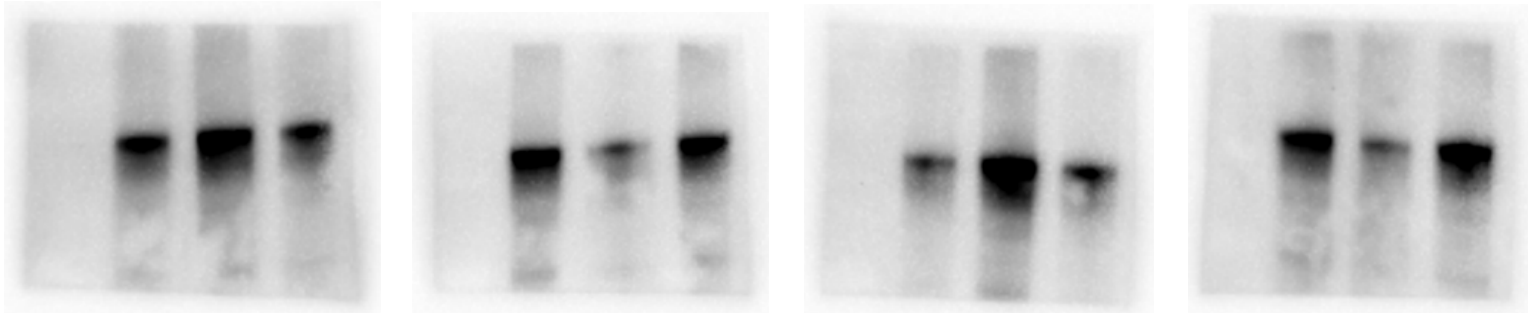

HIF-1α

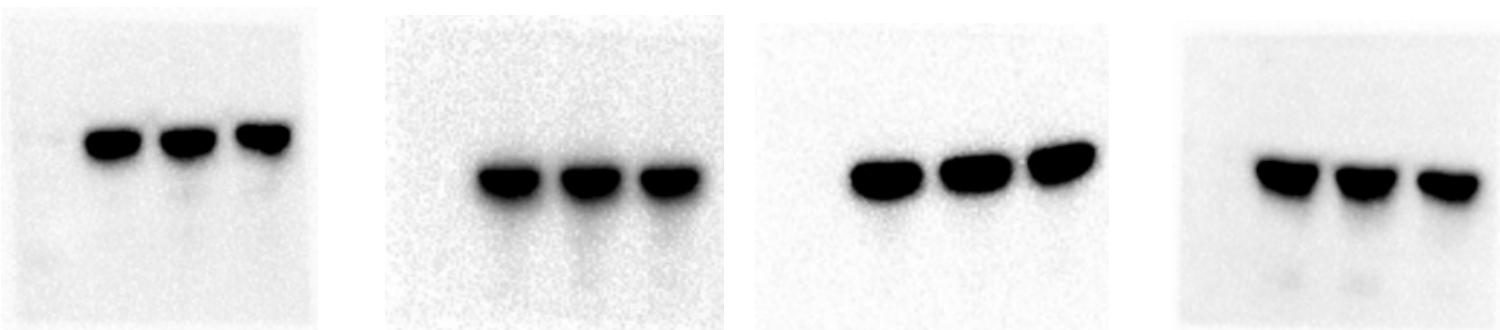

β-actin

Figure 3J

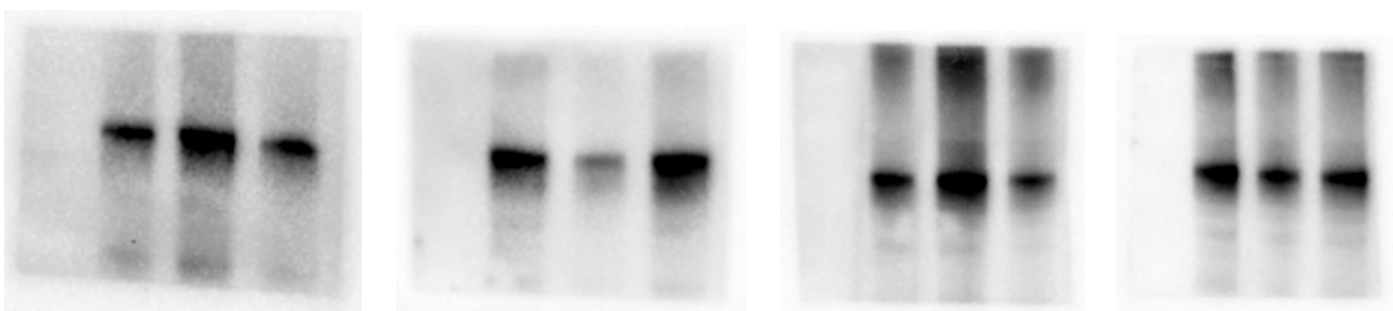

HIF-1α

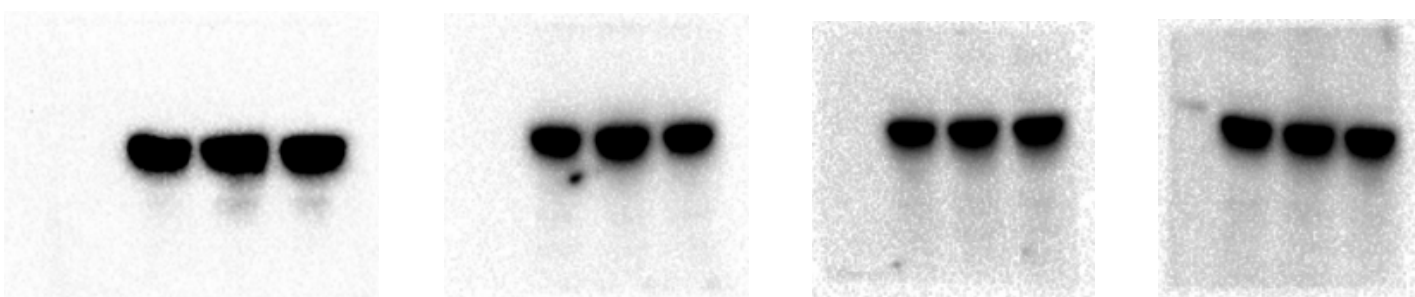

β-actin

Figure 3L

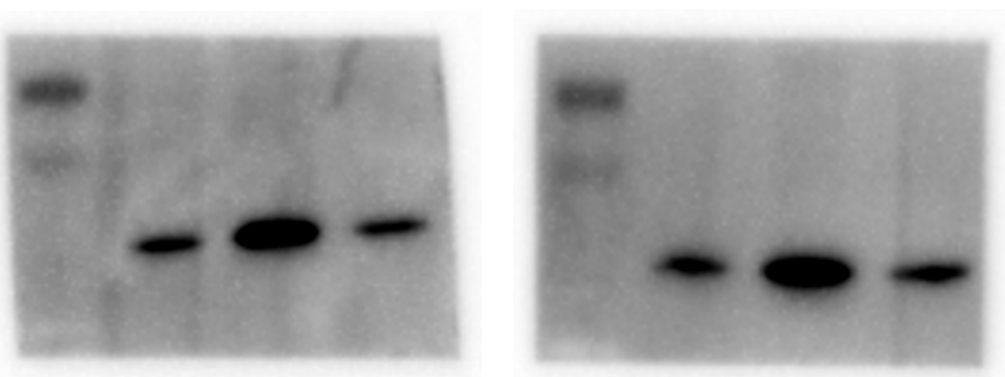

γH2AX

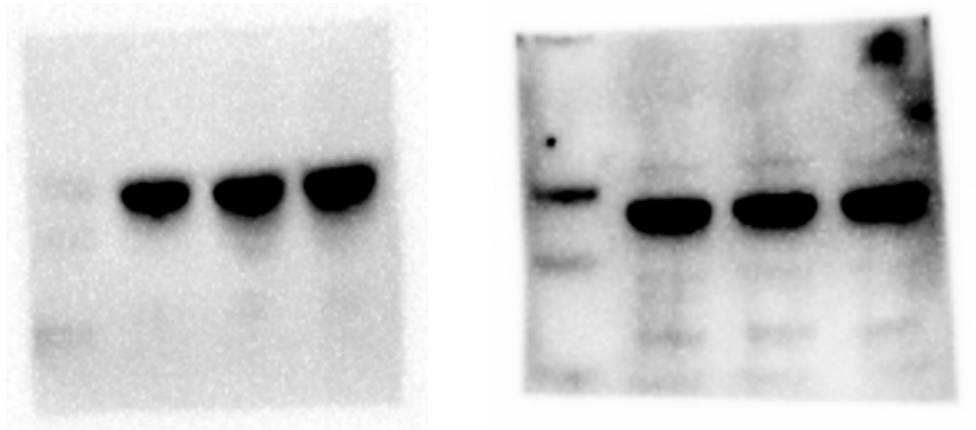

β-actin

Figure 3M

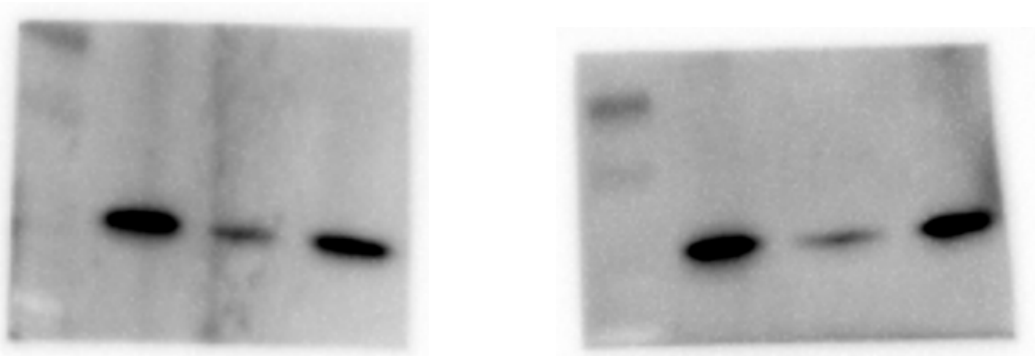

γH2AX

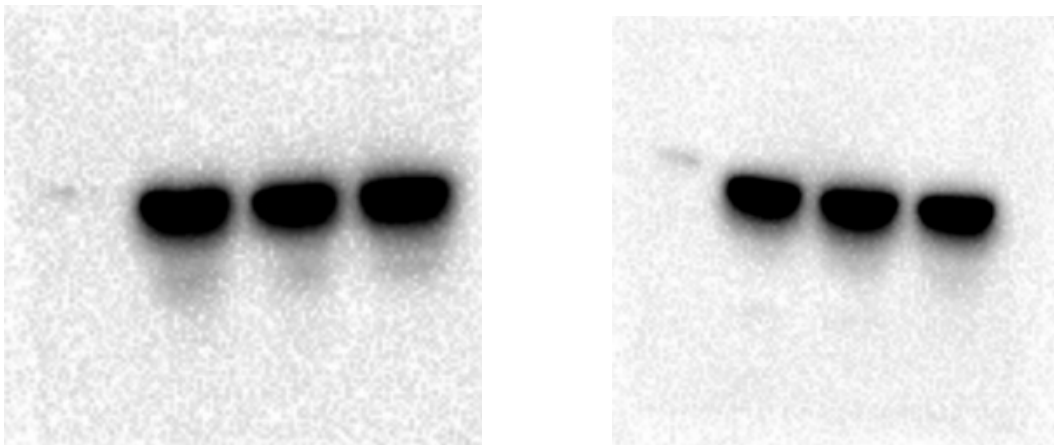

β-actin

Figure 3N

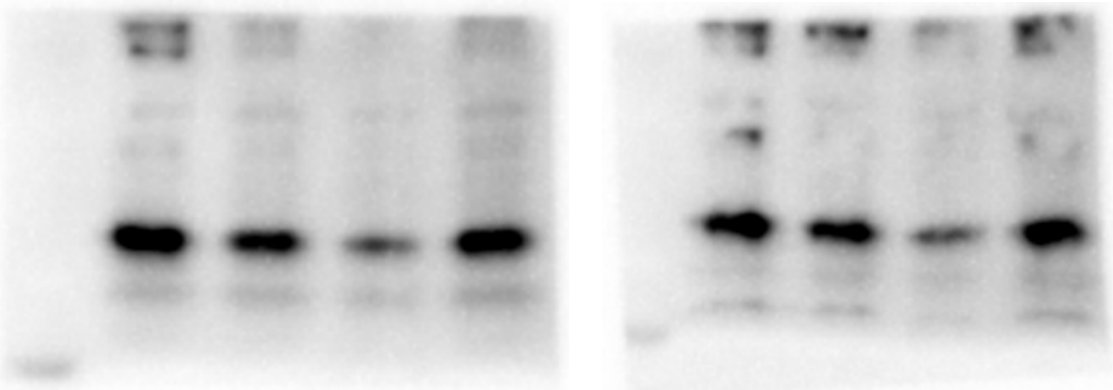

γH2AX

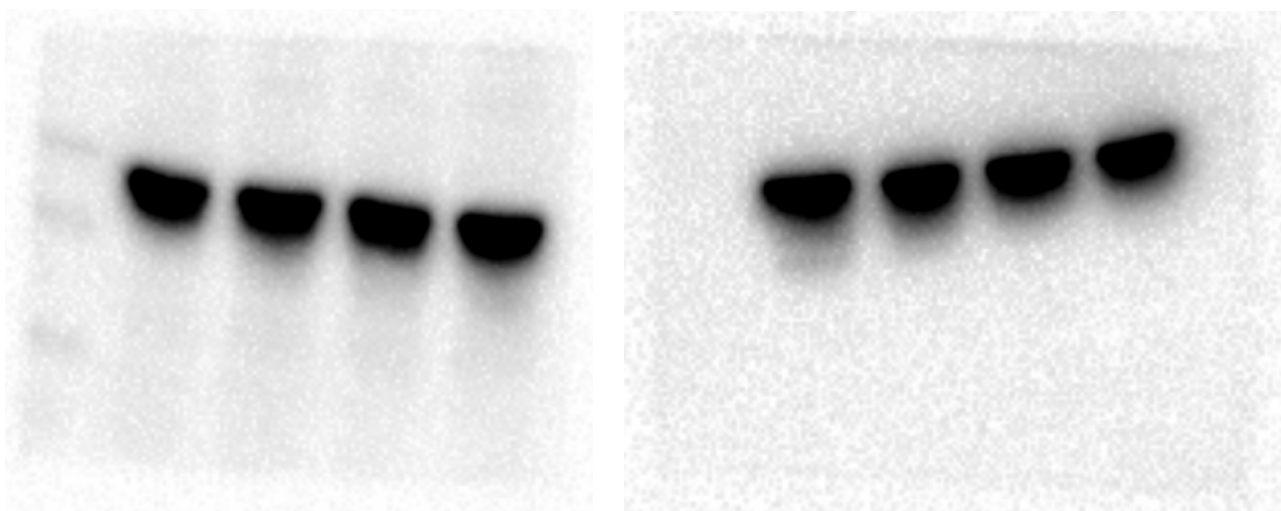

β-actin

Figure 3Q

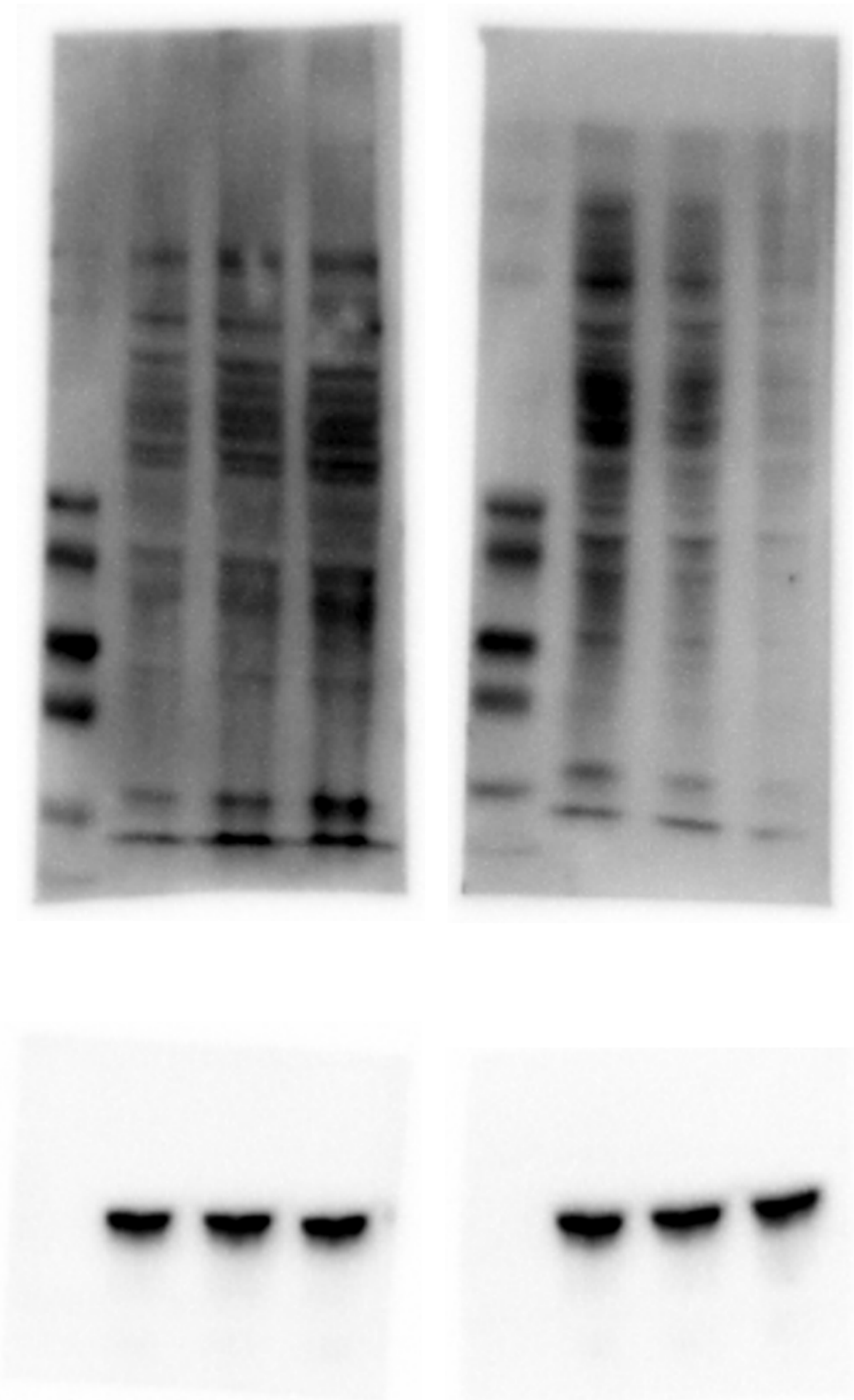

Pan-Lac

β-actin

Figure 3R

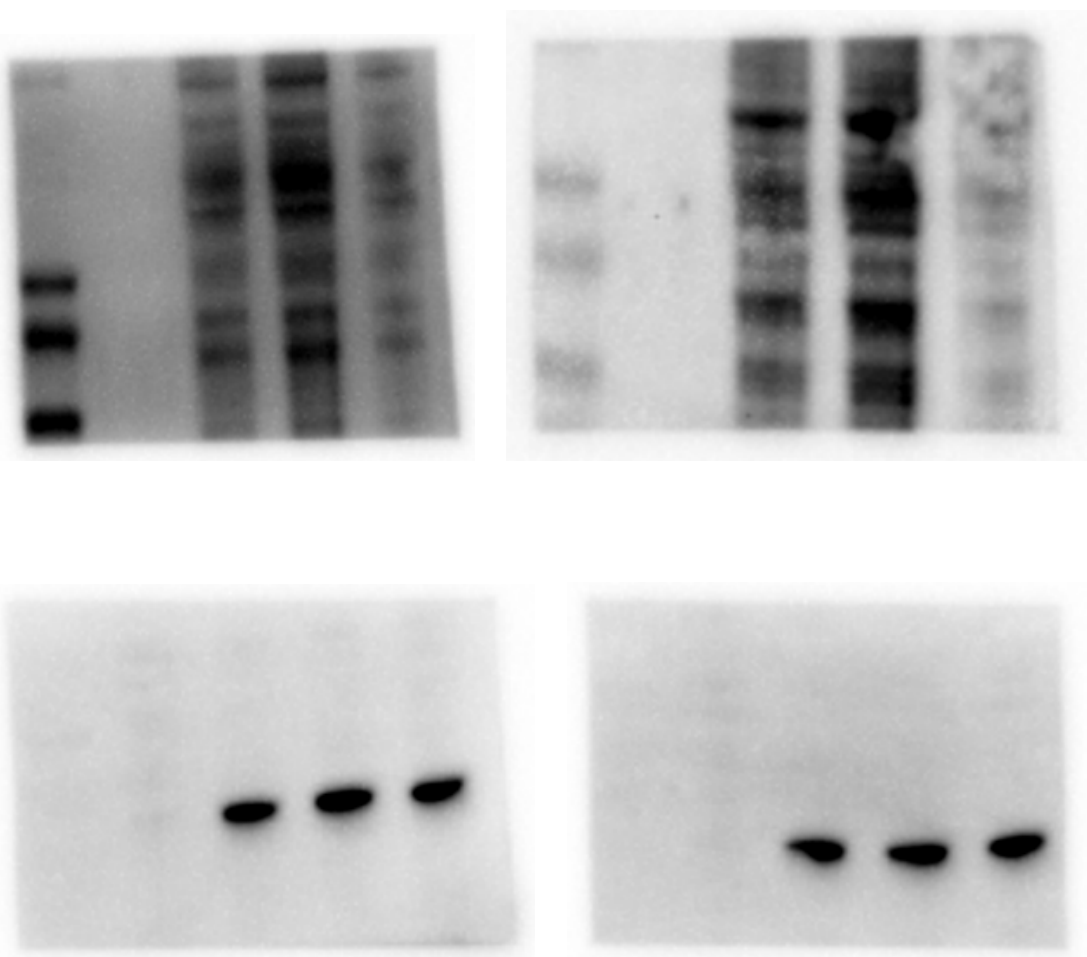

Pan-Lac

Flag

Figure 4F

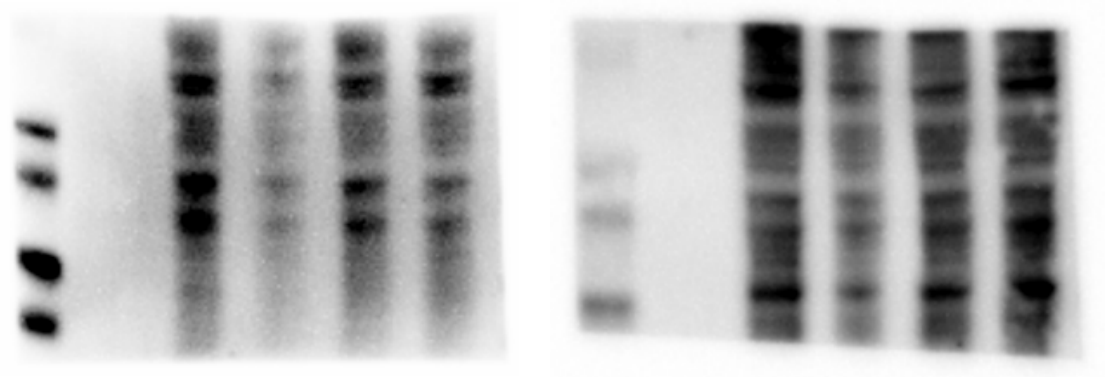

Pan-Lac

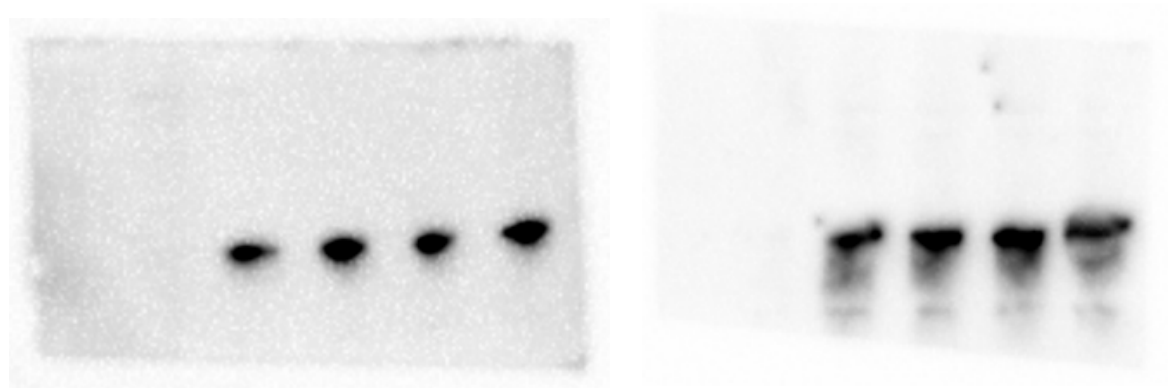

Flag

Figure 4G

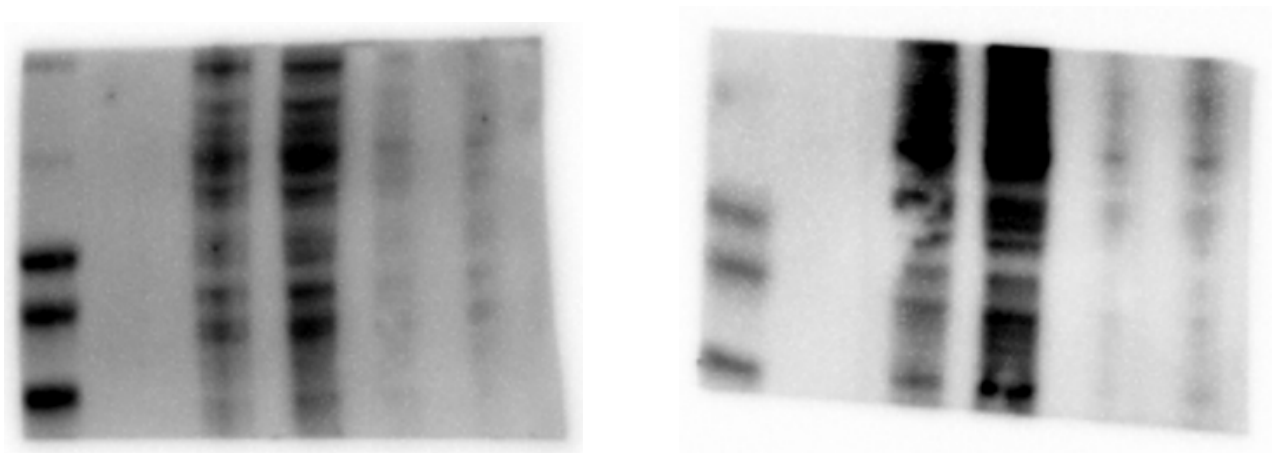

Pan-Lac

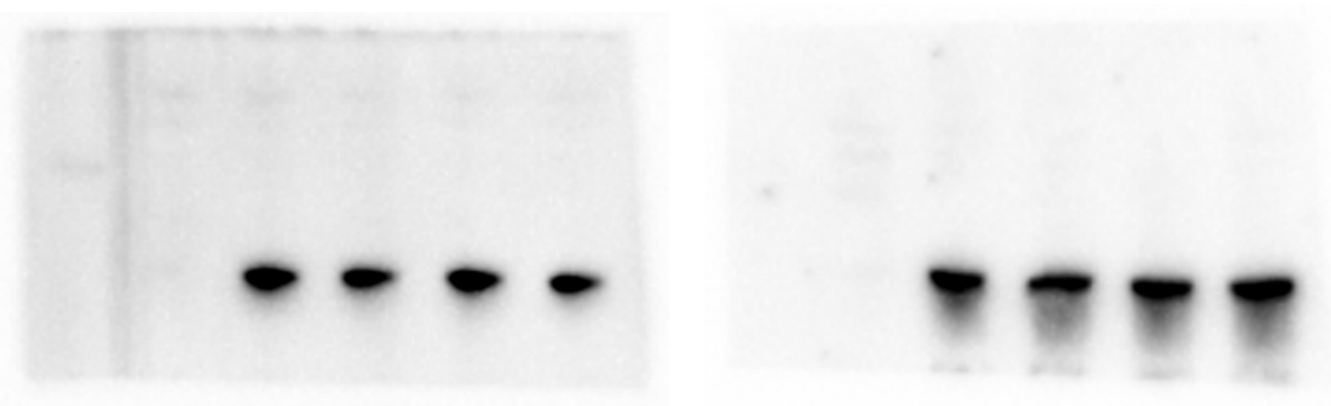

Flag

Figure 4I

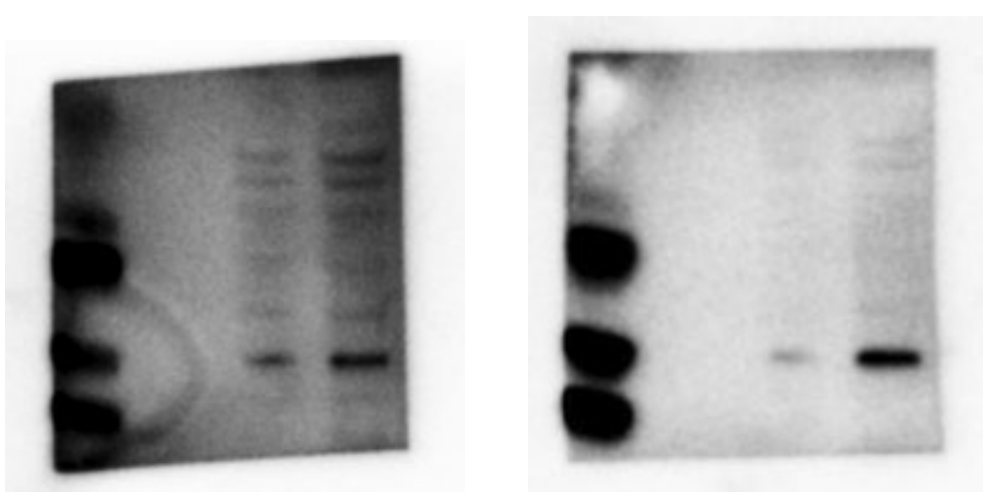

EFHD2-K9la

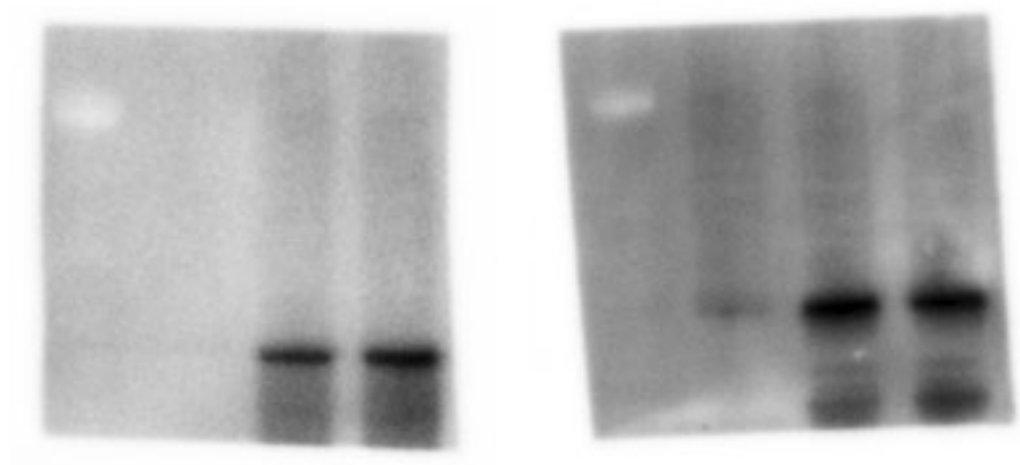

Flag

Figure 4J

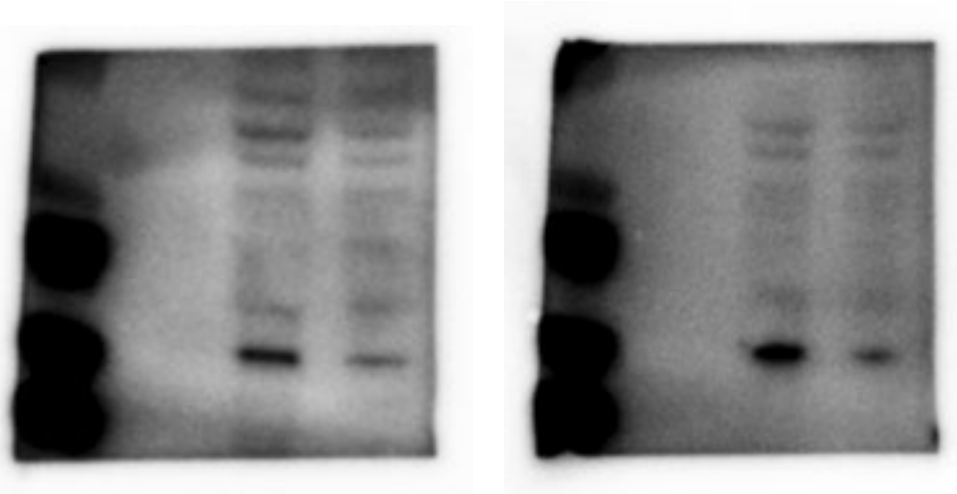

EFHD2-K9la

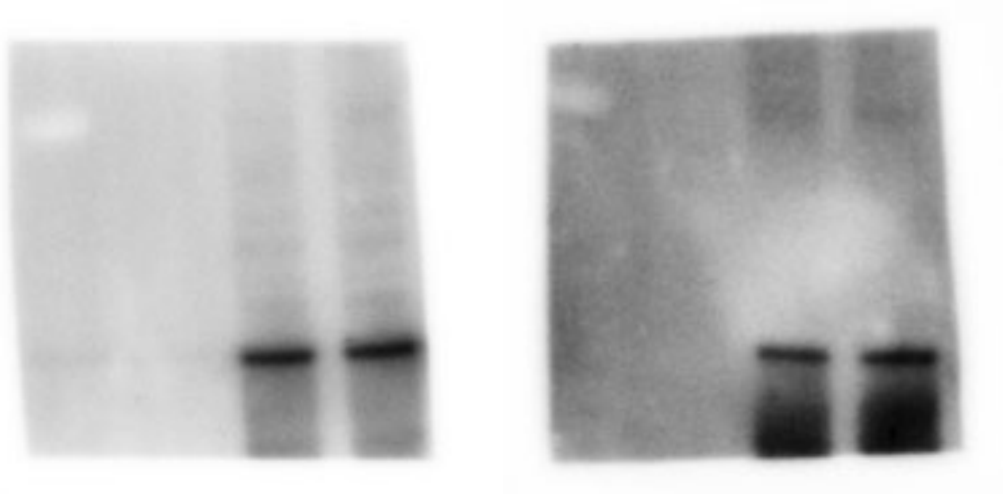

Flag

Figure 4K

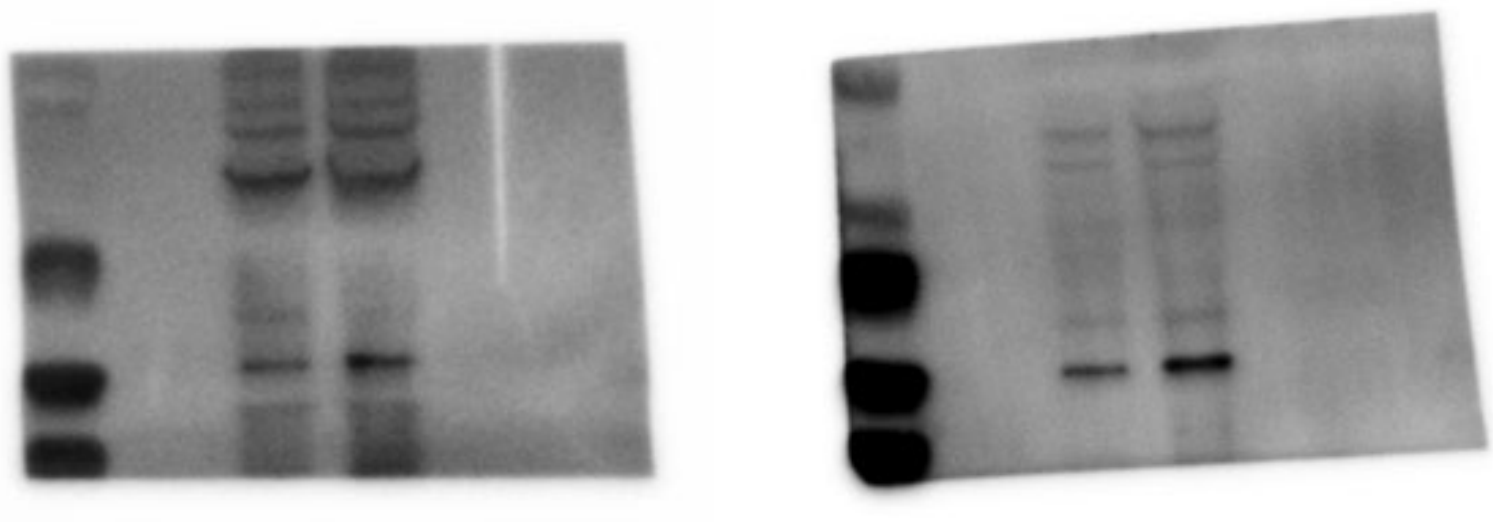

EFHD2-K9la

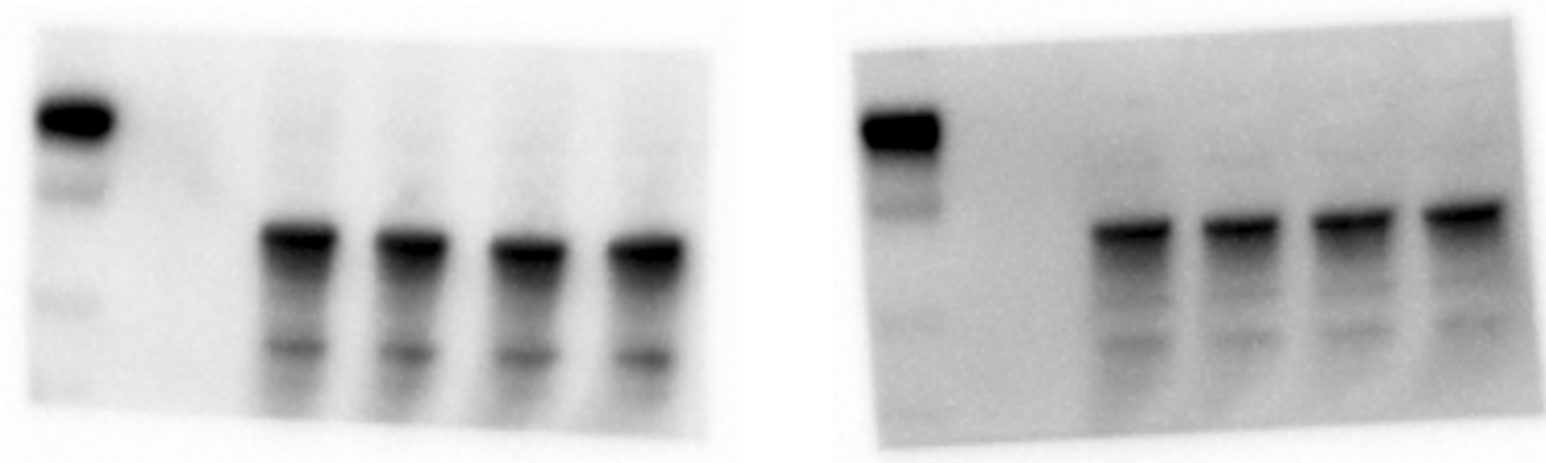

Flag

Figure 4O

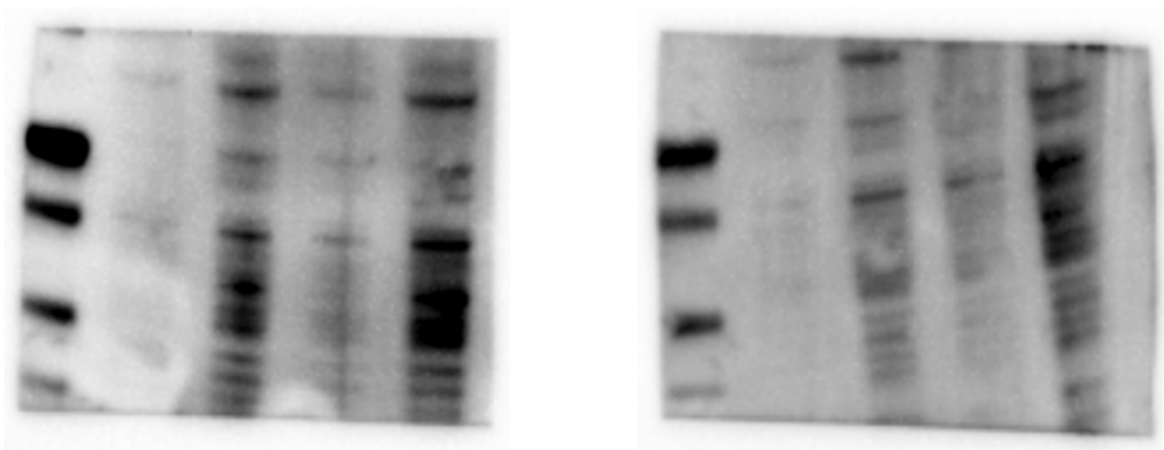

p-RPA2

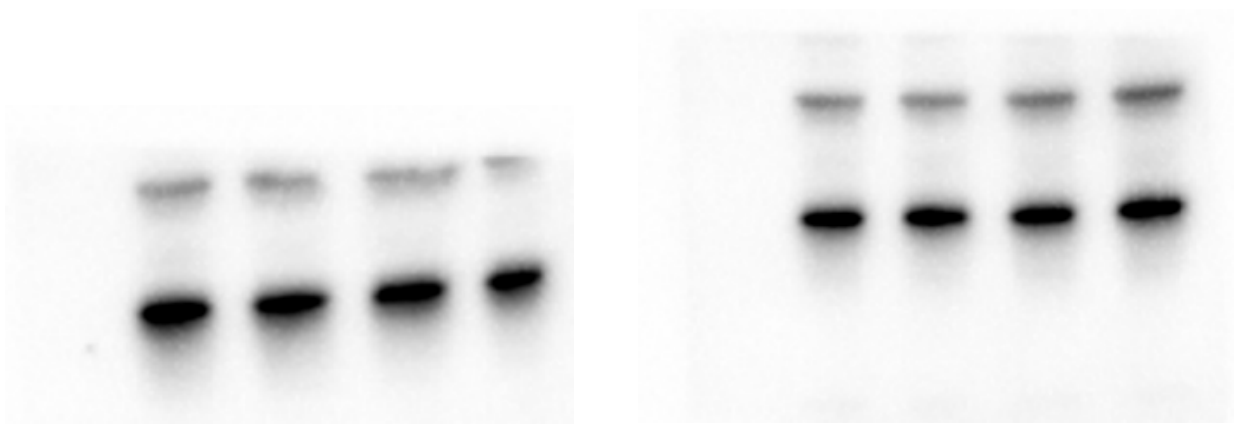

RPA2

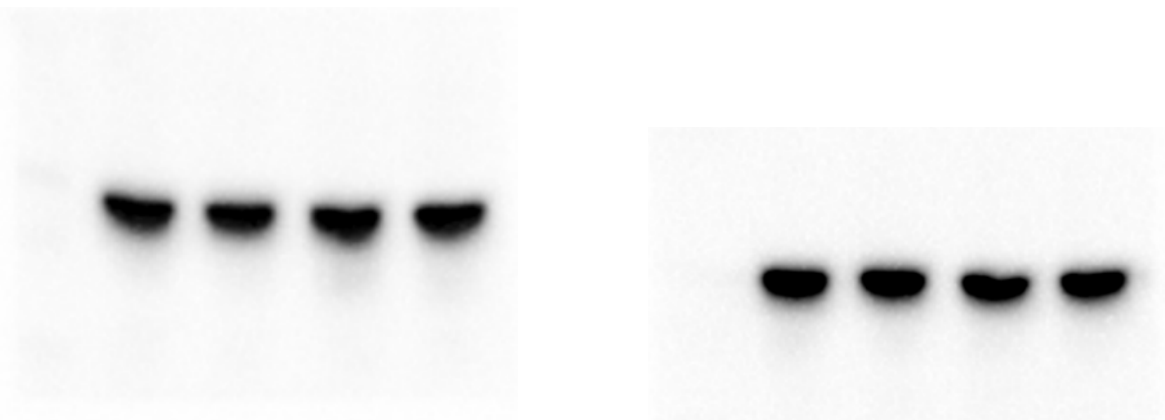

β-actin

Figure 4T

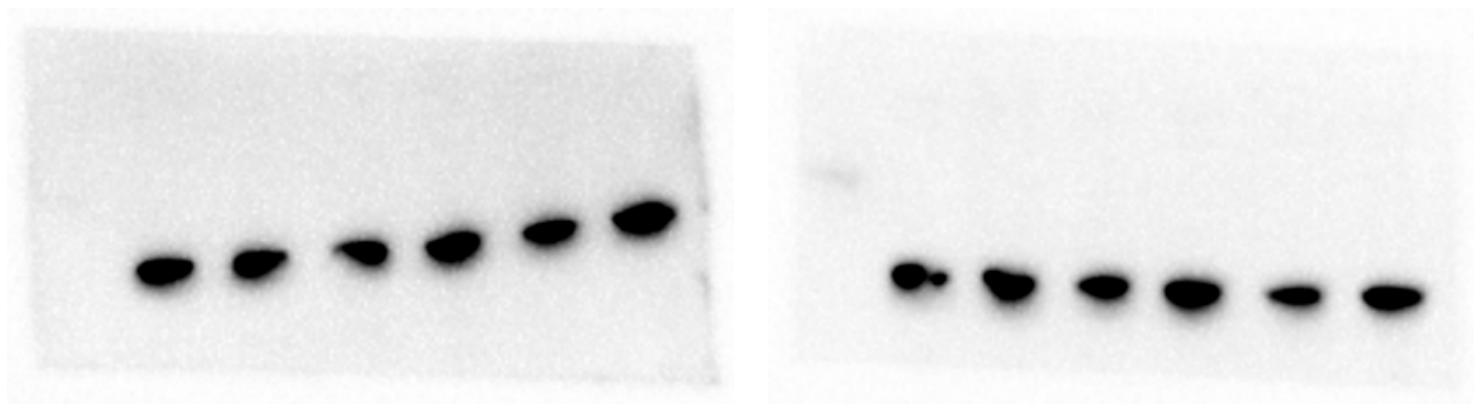

Flag

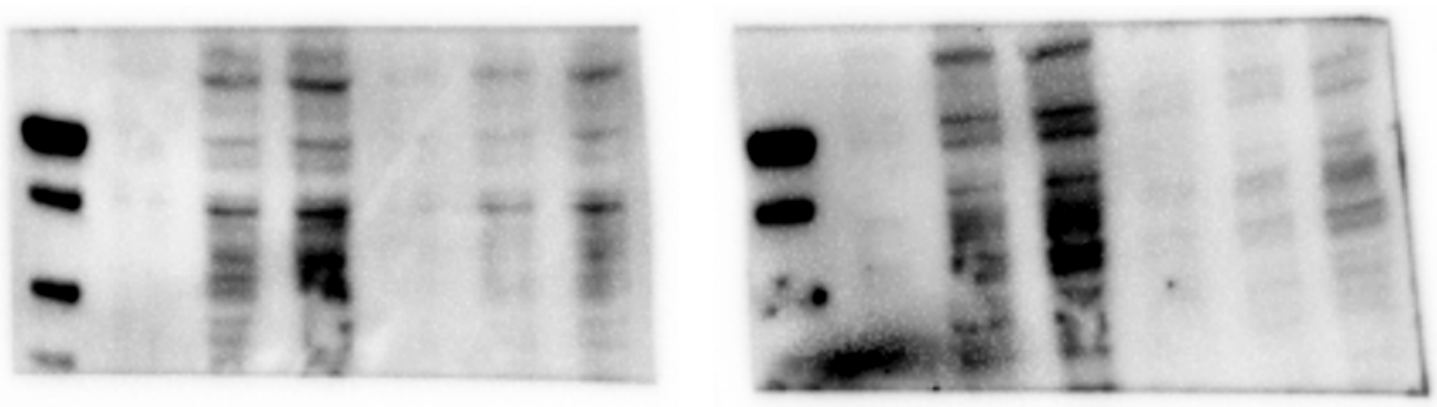

p-RPA2

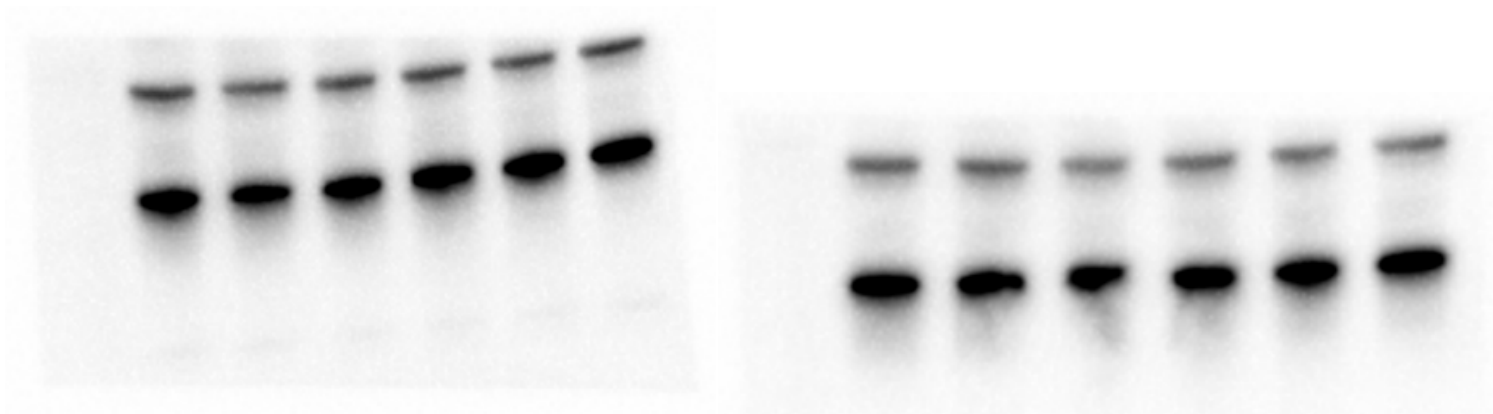

RPA2

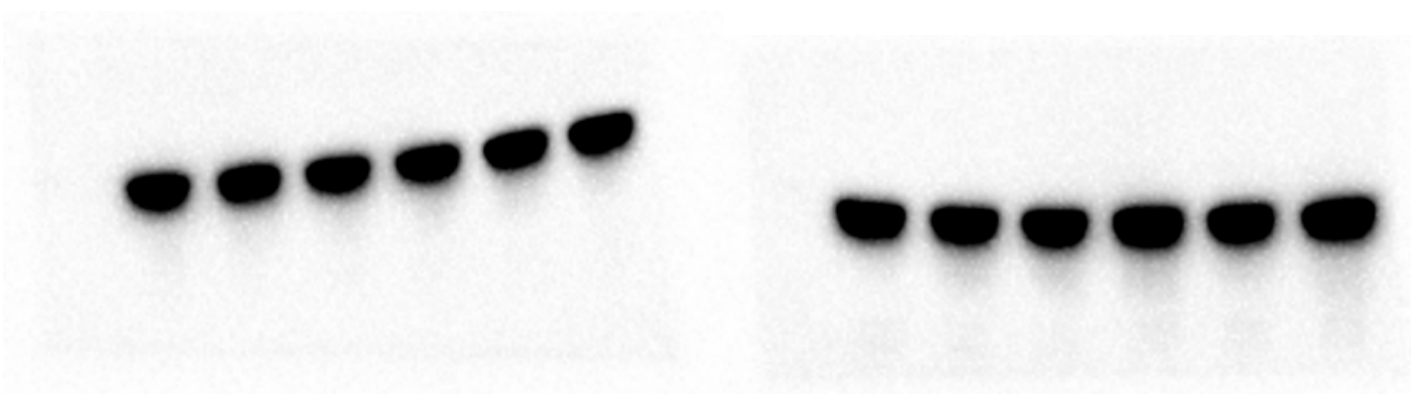

β-actin

Figure 5A

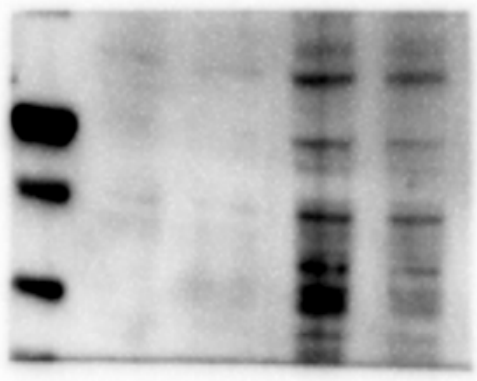

p-RPA2

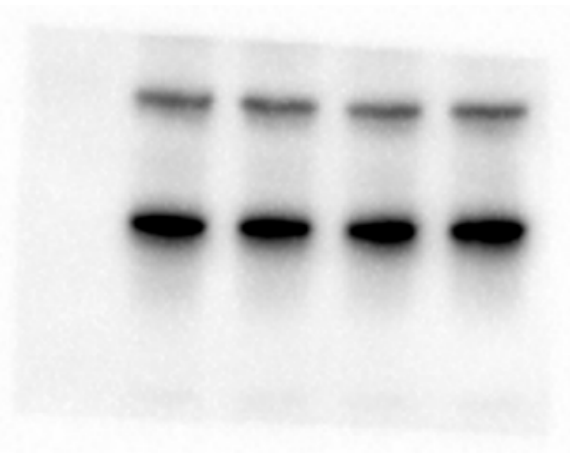

RPA2

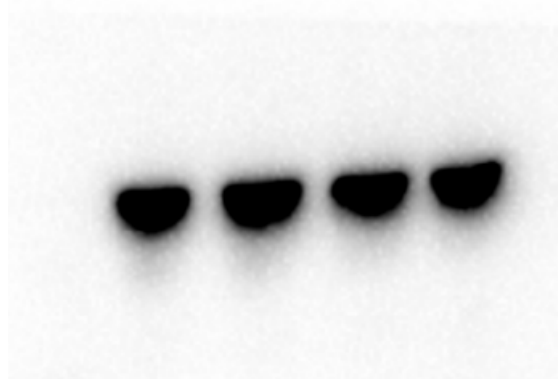

β-actin

Figure 5B

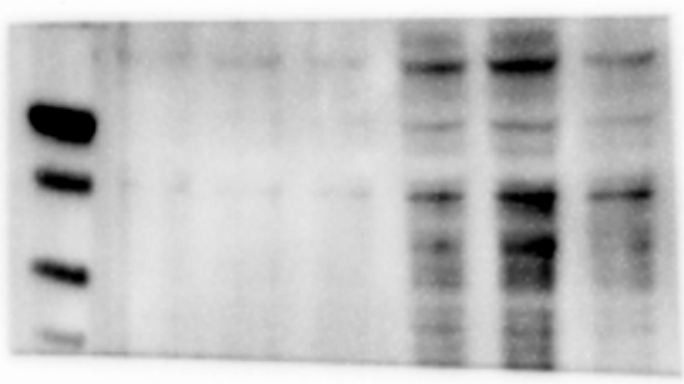

p-RPA2

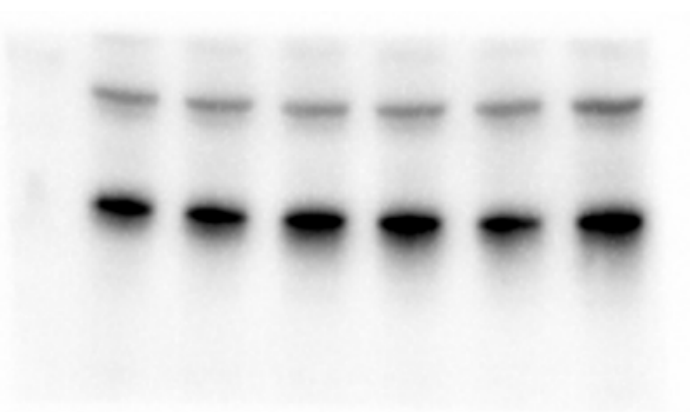

RPA 2

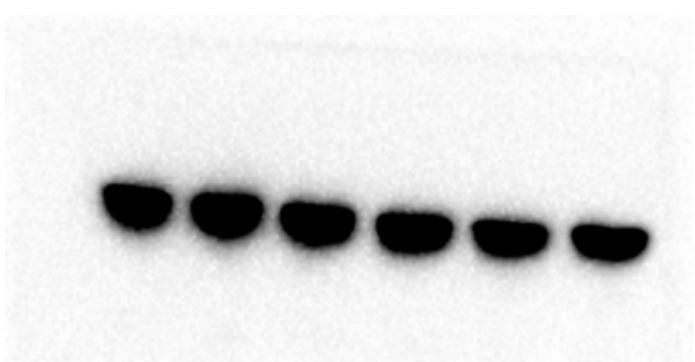

β-actin

Figure 5C

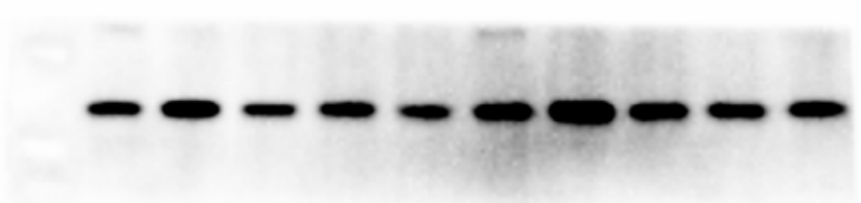

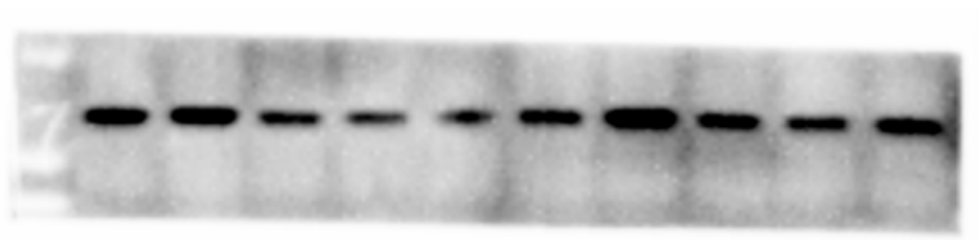

γH2AX

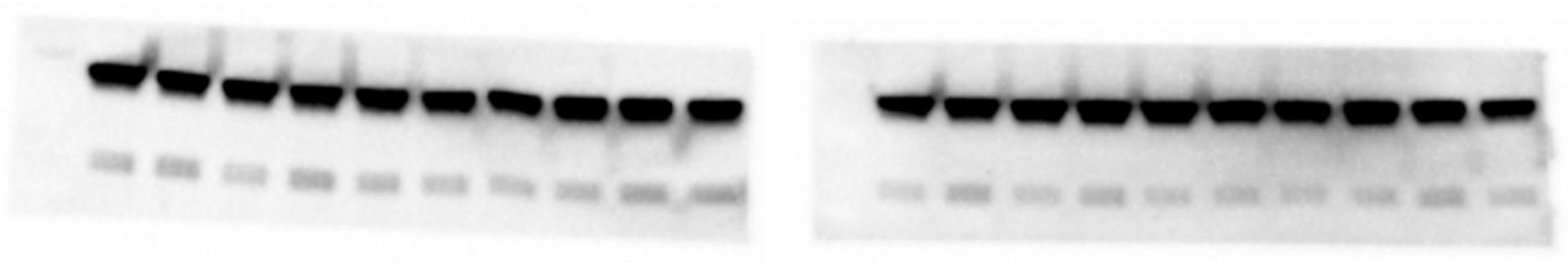

β-actin

Figure 6B

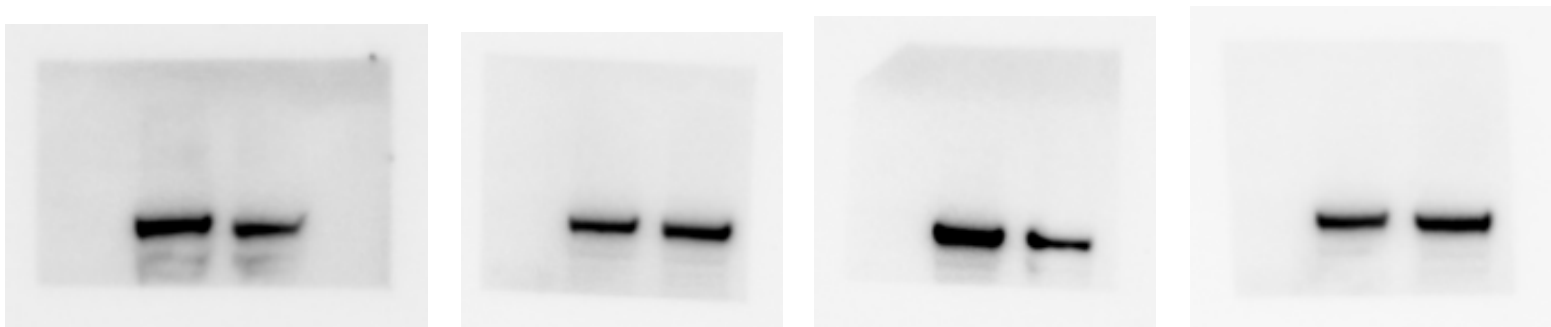

CD163

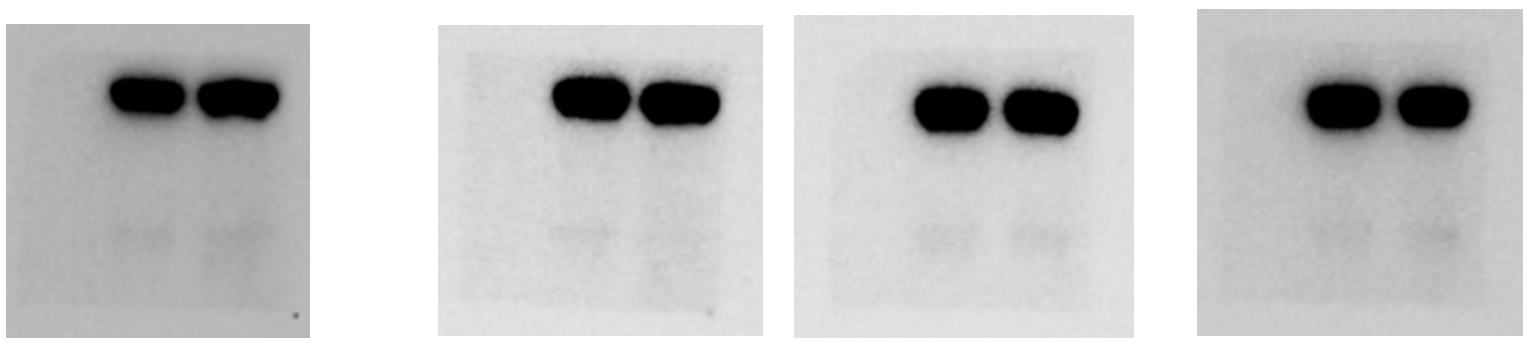

β-actin

Figure 6C

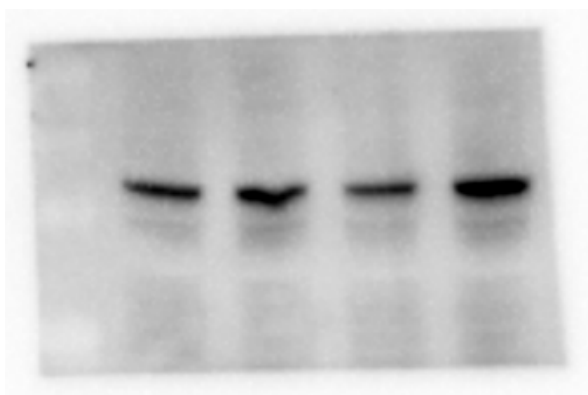

CD163

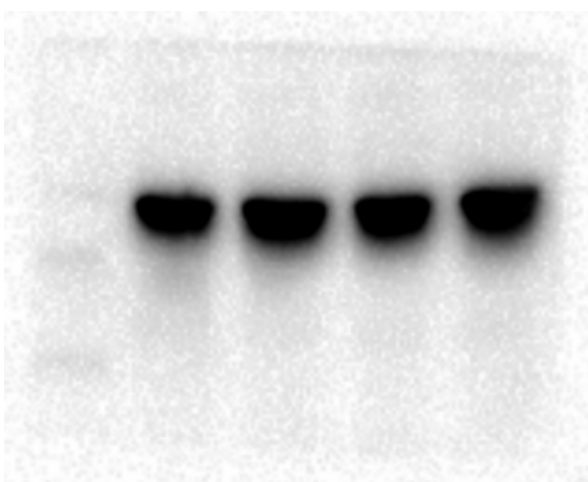

β-actin

Figure 8A

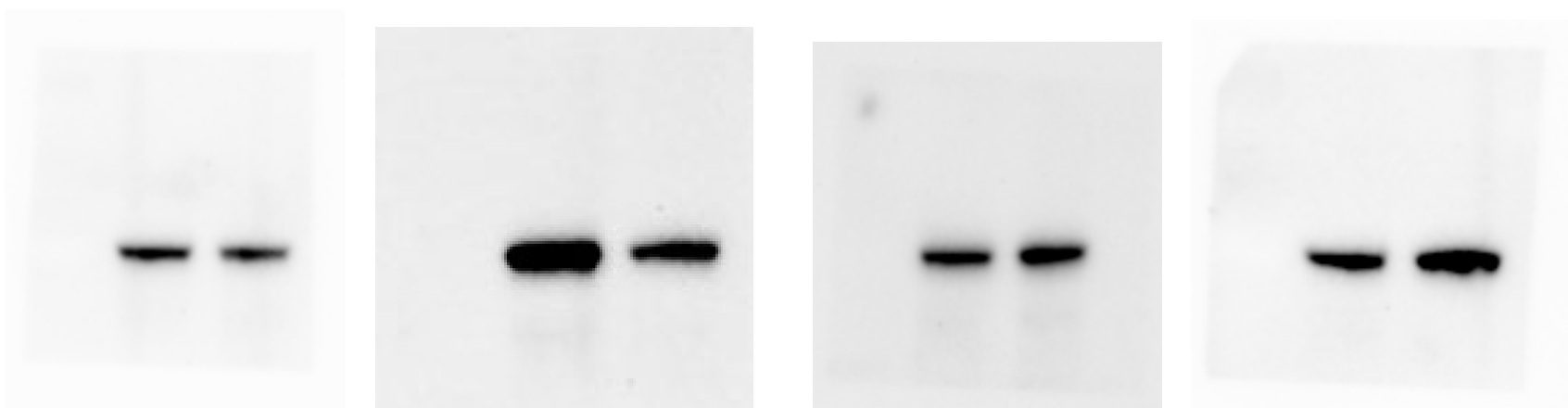

p-NF-κB P65

NF-κB P65

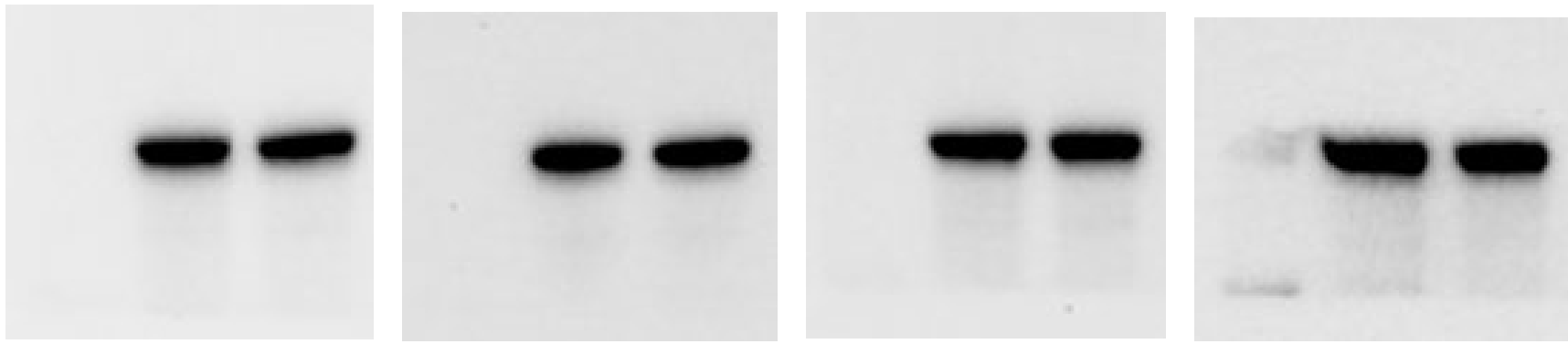

p-IκBα

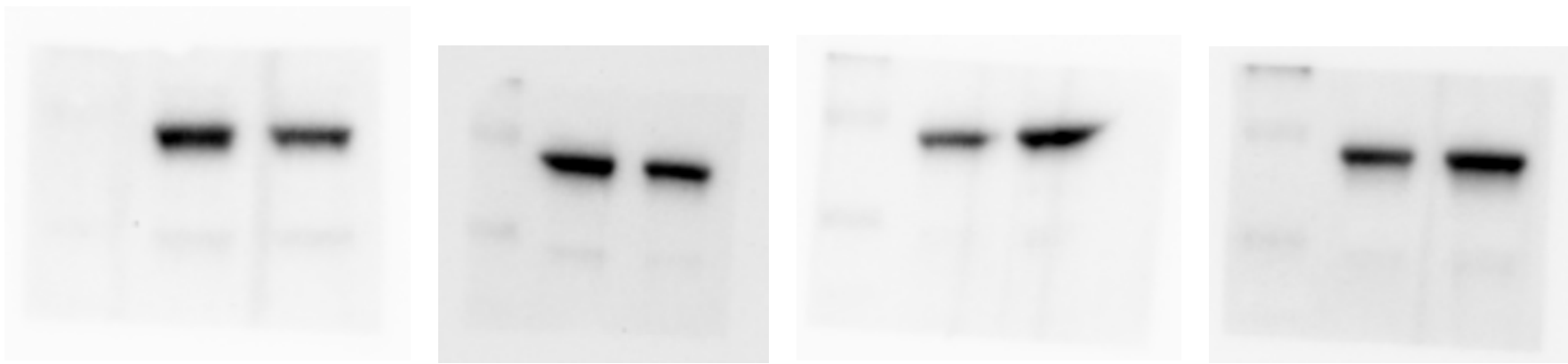

IκBα

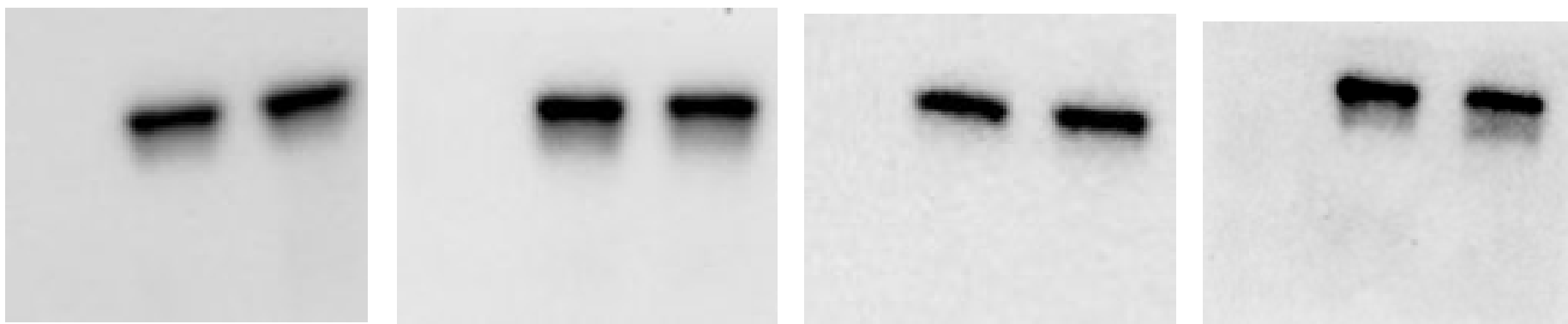

p-IKKα/β

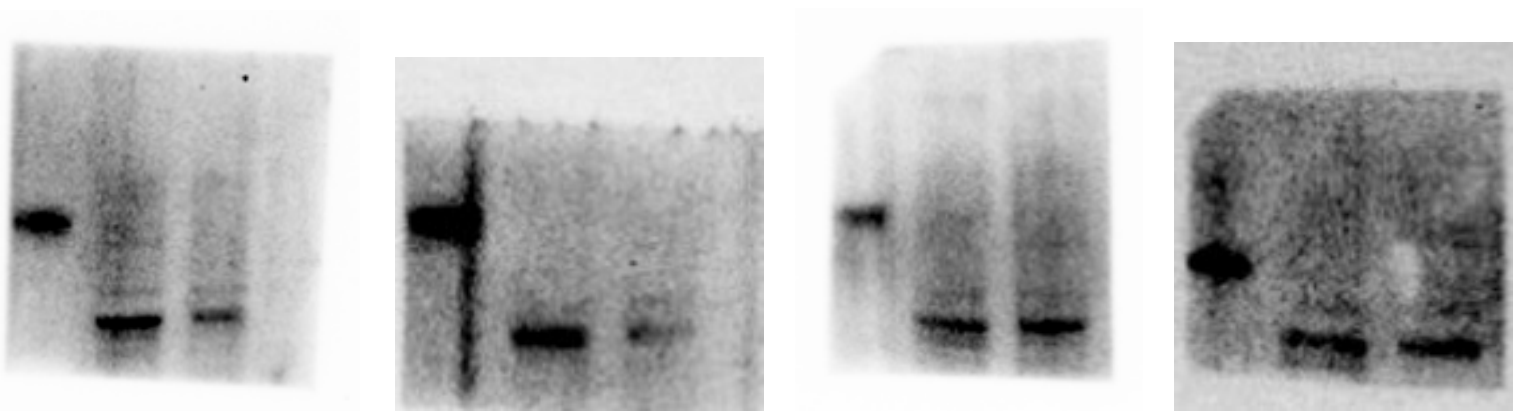

IKKα

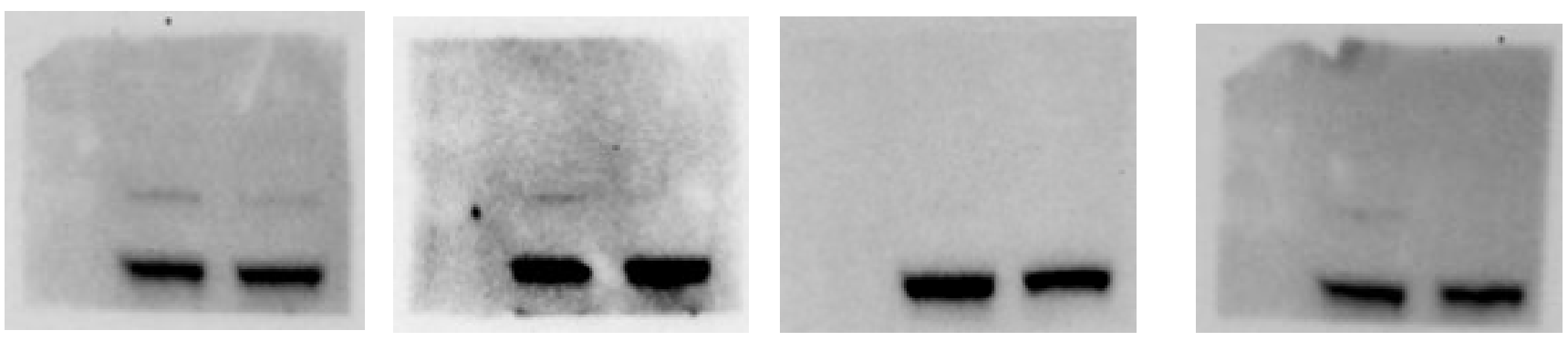

IKKβ

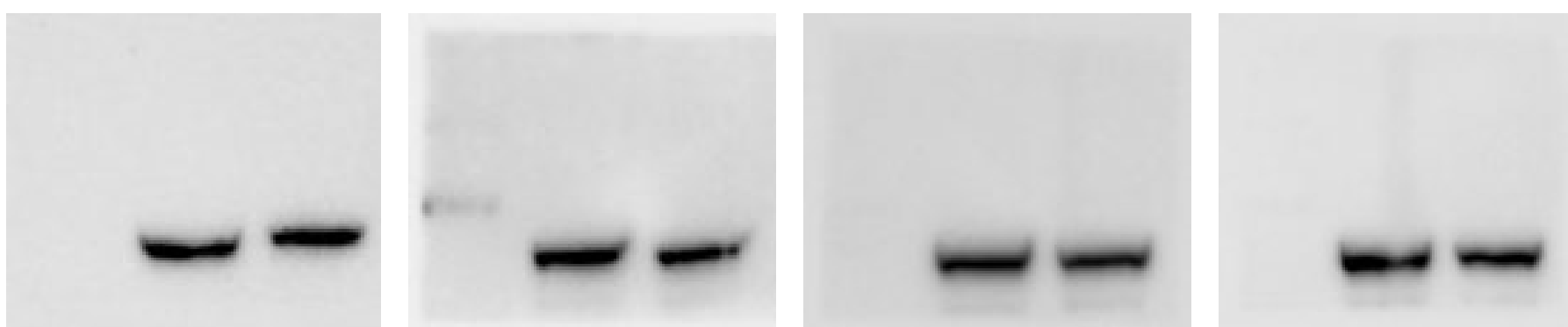

PD-L1

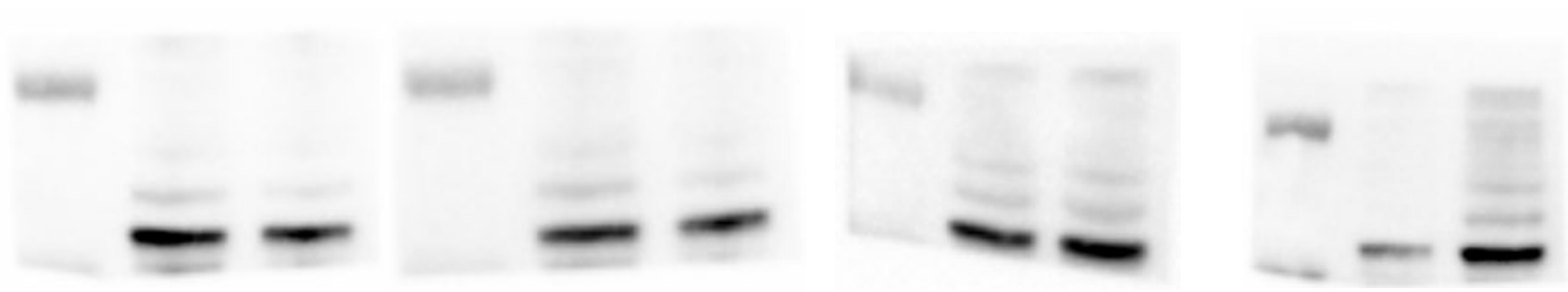

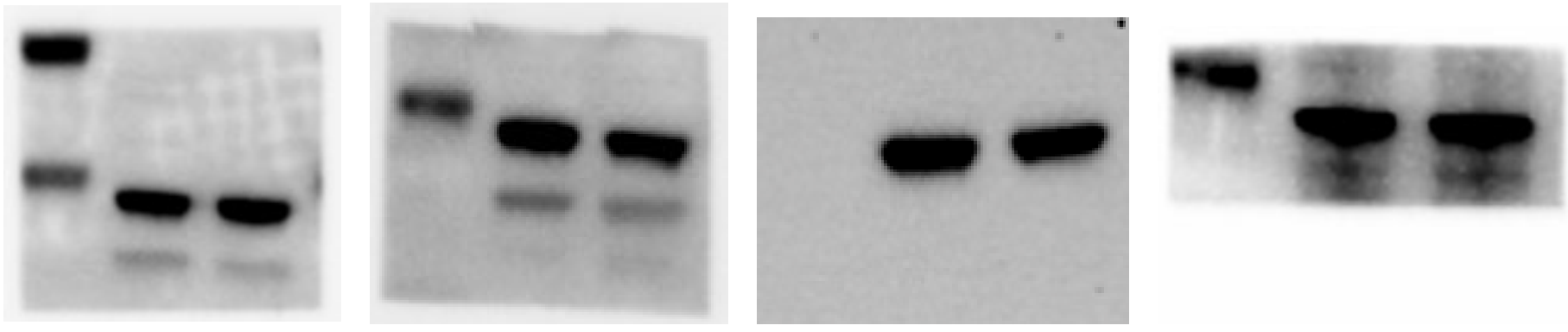

β-actin

Figure 8C

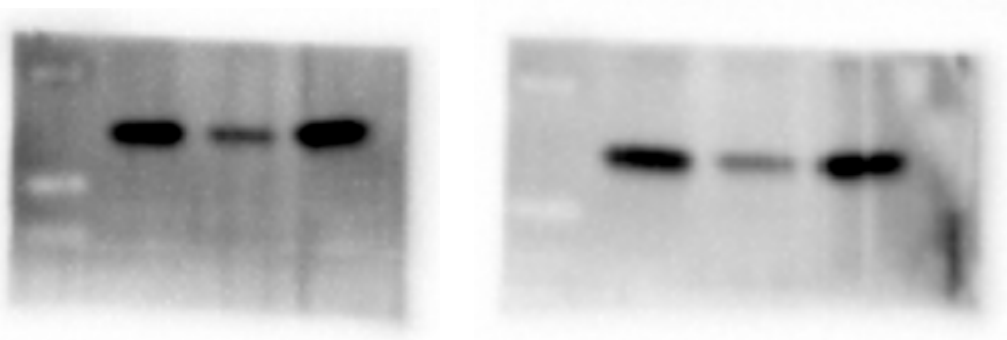

γH2AX

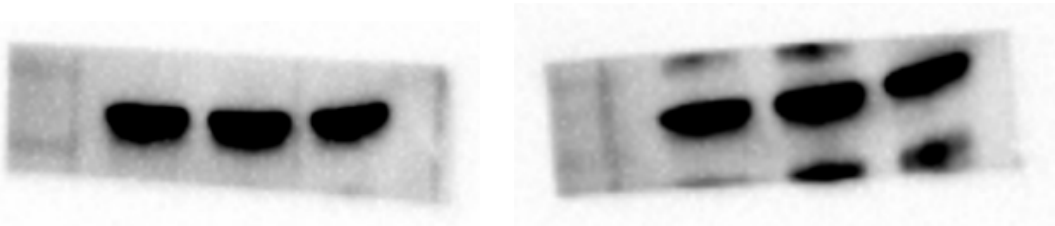

β-actin

Supplementary Figure 6D

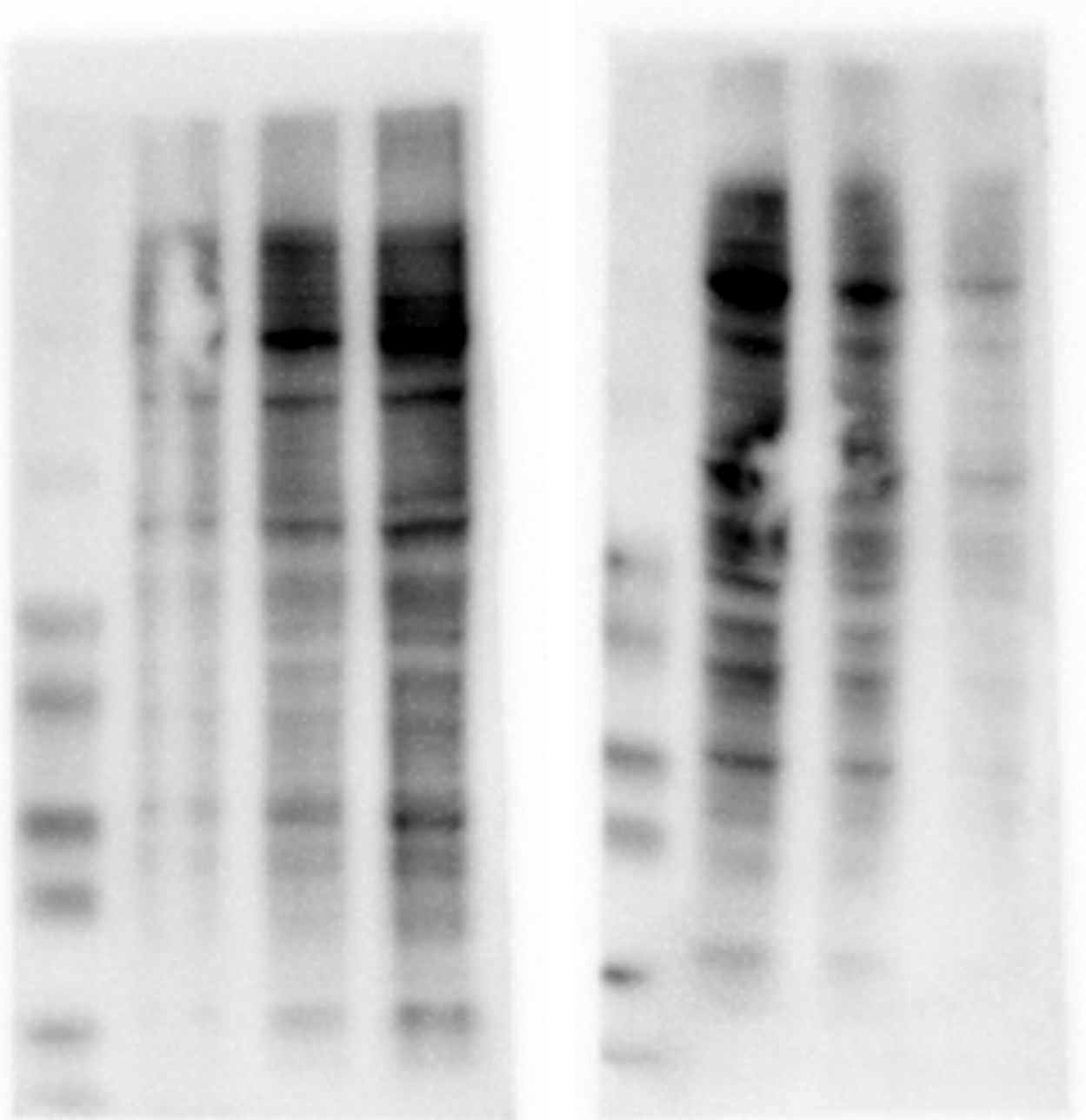

Pan-Lac

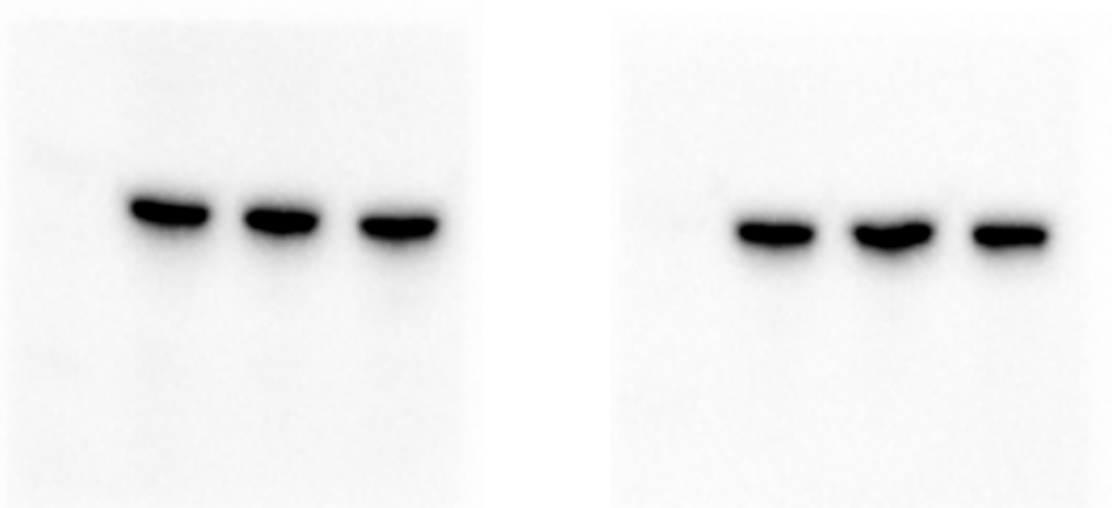

β-actin

Supplementary Figure 10A

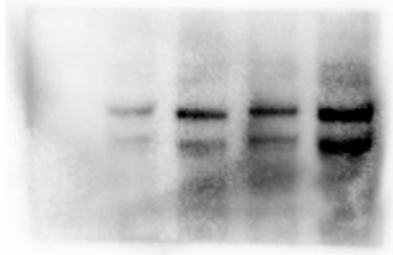

CD163

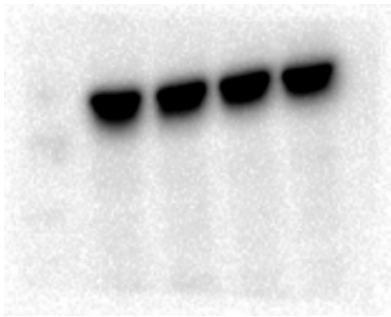

β-actin
