# Supplementary material for: EFHD2 drives lactate-mediated DNA damage repair and immunosuppression via HMGB1 and HIF-1α to confer radioresistance in colorectal cancer
Source: Cell Death Dis. 2026 Jun 23;17(1):777. doi: 10.1038/s41419-026-09031-2 (PMC13542245; doi:10.1038/s41419-026-09031-2)
Supplement: Supplementary file 2 [file 41419_2026_9031_MOESM1_ESM.docx]

**Supplementary Materials for**

**EFHD2 drives lactate-mediated DNA damage repair and immunosuppression via HMGB1 and** **HIF-1α to confer radioresistance in colorectal cancer**

Yilin Yu^1,2#^, Haixia Wu^3#^, Yu Xiao^4#^, Jianjian Qiu^1,2^, Liang Hong^1,2^, Baihua Yang^1,2^, Jianmin Wang^5^, Zhiping Wang^6*^, Lingdong Shao^1,2*^, Benhua Xu^7*^, Junxin Wu^1,2*^

^1^Department of Radiation Oncology, Clinical Oncology School of Fujian Medical University, Fujian Cancer Hospital (Fujian Branch of Fudan University Shanghai Cancer Center), Fuzhou, 350014, China

^2^NHC Key Laboratory of Cancer and Metabolism (Fujian Cancer Hospital), Fuzhou, 350014, China

^3^Clinical Oncology School of Fujian Medical University, Fujian Cancer Hospital (Fujian Branch of Fudan University Shanghai Cancer Center), Fuzhou, 350014, China

^4^Department of Radiation Oncology, The Second Affiliated Hospital of Chengdu Medical College, Nuclear Industry 416 Hospital, Chengdu, 610051, China

^5^Innovation Center for Cancer Research, Clinical Oncology School of Fujian Medical University, Fujian Cancer Hospital (Fujian Branch of Fudan University Shanghai Cancer Center), Fuzhou, 350014, China

^6^Department of Radiotherapy, Cancer Center, the First Affiliated Hospital of Fujian Medical University, Fuzhou, 350005, China; Department of Radiotherapy, National Regional Medical Center, Binhai Campus of the First Affiliated Hospital of Fujian Medical University, Fuzhou 350212, China

^7^Department of Radiation Oncology, Fujian Medical University Union Hospital, Fuzhou, 350001, China

Correspondence:

Junxin Wu	Email: junxinwufj@aliyun.com

Benhua Xu	Email: benhuaxu@yeah.net

Lingdong Shao	Email: shaolingdong4854@163.com

Zhiping Wang	Email: 707770685@qq.com

#Yilin Yu, Haixia Wu, and Yu Xiao contributed equally as first authors to this manuscript

*Junxin Wu, Benhua Xu, Lingdong Shao, and Zhiping Wang contributed equally as corresponding authors to this manuscript

**Caption for supplementary material**

**Graphic abstract**

**Supplementary experimental procedures**

**Supplementary Figure 1**

**Supplementary Figure 2**

**Supplementary Figure 3**

**Supplementary Figure 4**

**Supplementary Figure 5**

**Supplementary Figure 6**

**Supplementary Figure 7**

**Supplementary Figure 8**

**Supplementary Figure 9**

**Supplementary Figure 10**

**Supplementary Figure 11**

**Supplementary Figure 12**

**Supplementary Table 1**

**Supplementary Table 2**

**Supplementary Table 3**

**Supplementary Table 4**

**Supplementary Table 5**

**Graphic abstract**

**Deciphering the lactate-driven hsa-let-7b-5p/EFHD2/HMGB1 network in colorectal cancer radiosensitivity and immunosuppression.** Radioresistant colorectal cancer exhibits hsa-let-7b-5p downregulation, leading to EFHD2 overexpression. Mechanistically, EFHD2 interacts with HMGB1, enhancing lactate metabolism via HIF-1α stabilization. Elevated lactate promotes homologous recombination repair through EFHD2 lysine-9 lactylation, resulting in reduced DNA damage (indicated by fewer γH2AX foci) and enhanced RPA2/RAD51 recruitment, while simultaneously fostering an immunosuppressive tumor microenvironment characterized by M2 macrophage polarization, Treg expansion, NF-κB activation, and PD-L1 upregulation. Restoring hsa-let-7b-5p or targeting EFHD2 lactylation disrupts this axis, enhancing radiosensitivity and overcoming therapy resistance, providing a novel strategy that combines DNA repair inhibition and metabolic-immune modulation for CRC treatment.

**Supplementary experimental procedures**

**Cell culture**

Human colorectal cancer cell lines (HCT116 and SW480) and the human monocytic leukemia cell line THP-1 were purchased from the National Collection of Authenticated Cell Cultures, Shanghai Institute of Biochemistry and Biological Sciences, the Chinese Academy of Sciences. HCT116 and SW480 cells were cultured in Dulbecco's Modified Eagle Medium (HCT116 and SW480) (DMEM; C11995500BT, Gibco, USA), while THP-1 cells were cultured in RPMI-1640 medium (11875093, Gibco, USA). Both media were supplemented with 10% fetal bovine serum (FBS; A0500-3010, Cegrogen, Germany) and 1% penicillin/streptomycin (ABK0005C, ABKBio, China). The cells were cultured in a humidified incubator at 37°C with 5% CO2. All cell lines were authenticated by short tandem repeat (STR) profiling and routinely tested for mycoplasma contamination.

**Irradiation treatment**

Cells were exposed to ionizing radiation (6-10 Gy) using an X-ray irradiator at a dose rate of 6 Gy/min. After irradiation, cells were collected or subjected to subsequent assays at different time points according to the specific experimental design.

**Identification of exosomes**

The extracellular vesicle-like structure of exosomes was observed and identified by the use of transmission electron microscopy (TEM; HT-7700; Hitachi, Japan). Next, a 10μL exosome suspension was applied to carbon-coated copper grids (200 mesh) and allowed to adsorb for 1 min. Then, 10 μL of uranyl acetate solution was applied to carbon-coated copper grids (200 mesh) for 1 min and air-dried. Lastly, the morphology and ultrastructure of the exosomes were then examined using an HT-7700 electron microscope at an accelerating voltage of 100 kilovolts. The concentration and size distribution of exosomes were examined using nanoparticle tracking analysis (NTA) with a NanoFCM device (N30E, China).

**Measurement of differentially expressed miRNAs in SW480 and SW480R exosomes**

First, the extracted total RNA samples were subjected to agarose electrophoresis and Nanodrop (ND-1000) quality control and quantification for library construction. Then, the library quality was determined by Agilent 2100 Bioanalyzer, and the mixed sequencing libraries of different samples were denatured to generate single-stranded DNA with 0.1 M NaOH. Finally, sequencing was performed on an Illumina sequencer according to the manufacturer's instructions.

**Measurement of differentially expressed proteins in SW480 and SW480R exosomes by label-free quantitative proteomics analysis**

The next step was to identify proteins with differential expression using a label-free quantitative proteomic analysis. After combining the exosome samples with RIPA lysis solution, protease inhibitors, and phenylmethylsulfonyl fluoride (PMSF; PMSF-RO, Sigma-Aldrich, USA), the samples were allowed to thaw completely on ice following the mixing process. We used the BCA method to quantify the protein after centrifuging the protein lysate at 14,000 g at 4°C and collecting the supernatant. Then, a series of procedures were performed, including acetone precipitation, protein redissolution, reduction, alkylation, enzymatic hydrolysis, sodium deoxycholate removal, and desalting. Peptides were separated on an EASY-nLC 1200nano-UPLC system and detected using an online mass spectrometer (Q-Exactive). The analysis was performed on a 100 μm ID × 15 cm reversed-phase column (Reprosil-Pur 120 C18-AQ, 1.9 μm, Dr. Maisch). The samples were directly injected into the column by an autosampler and then separated on the column at a flow rate of 300 nL/min with a gradient time of 120 min. The mass spectrometry analysis time was 120 min/sample, in positive ion detection mode, with a parent ion scanning range of 350-1,600 m/z. Raw files were processed using MaxQuant (version 2.0.1.0). The protein database was obtained from the UNIPROT database (uniprot-proteome-human-9606-UP000005640-2023.4.fasta).

**Gene expression, correlation and survival analysis**

Gene expression data and corresponding clinical information for RC were obtained from The Cancer Genome Atlas (TCGA) database as well as multiple Gene Expression Omnibus (GEO) datasets, including GSE35449^1^, GSE38389^2^, GSE40492 (https://www.ncbi.nlm.nih.gov/geo/query/acc.cgi?acc=GSE40492), GSE41655 (https://www.ncbi.nlm.nih.gov/geo/query/acc.cgi?acc=GSE41655), GSE55536^3^, GSE57614^4^, GSE83924^5^, GSE86298^6, 7^, GSE126093^8^, GSE149377^9^, GSE151194^10^, GSE173611^11^, GSE179543^12^, GSE189847^13^, and GSE223325^14^. Kaplan-Meier survival curves were generated using the “survminer” and “survival” packages in R, and statistical significance was evaluated with the log-rank test. Correlations between quantitative variables were assessed using either Pearson’s or Spearman’s correlation analysis, depending on data distribution.

**Differential analysis of miRNAs and mRNAs**

Differential expression of miRNAs between groups was analyzed using the edgeR package, with significantly dysregulated miRNAs defined by a fold change > 1.5 and a *p*-value < 0.05.

**Prediction of miRNA target genes**

Potential target genes of hsa-let-7b-5p were predicted using seven databases: miRTarBase, miRDB, PicTar, TargetScan, miRanda, microT, and PITA. Overlapping targets identified across these databases were determined through Venn diagram analysis.

**Functional enrichment analysis**

Functional and pathway enrichment analyses were performed using the “clusterProfiler” R package based on Gene Ontology (GO) and Kyoto Encyclopedia of Genes and Genomes (KEGG) terms. A *p*-value < 0.05 was considered statistically significant. GO analysis was divided into three categories of gene function: biological processes (BP), cellular components (CC), and molecular functions (MF).

**Immune infiltration assessment**

Immune cell infiltration in RC was analyzed using multiple algorithms in R, including CIBERSORT^15^, EPIC^16^, quanTIseq^17^, MCPcounter^18^, xCell^19^, ESTIMATE^20^, and TIMER^21^. Spearman correlation analysis was performed to assess the relationship between gene expression and immune cell infiltration levels. Gene Set Enrichment Analysis (GSEA) was conducted using the “clusterProfiler” R package to identify significantly enriched functions and pathways. Gene sets were obtained from the MSigDB database, and results with *p*-value < 0.05 were considered statistically significant.

**Cell transfection**

Lentiviral overexpression of hsa-let-7b-5p was obtained from Hippobio (Zhejiang, China). Small interfering RNA (siRNA) targeting HMGB1 (si-HMGB1) was synthesized by Hippobio for HMGB1 knockdown. The experimental cells were transfected using RNATransMate (Sangon) according to the manufacturer’s instructions. Lentiviral vectors for EFHD2 knockdown and overexpression, as well as HMGB1 overexpression, were purchased from Hippobio. Target cells were transduced with the corresponding lentiviruses following the manufacturer’s protocol. Stable cell lines were established by selection with puromycin, and knockdown or overexpression efficiency was confirmed by quantitative real-time polymerase chain reaction and Western blotting. Importantly, following the establishment of these stably transfected radioresistant cell lines, cells used to assess intrinsic properties were cultured without additional irradiation, whereas cells intended for radiation response assays were irradiated immediately prior to the experiment. This approach ensured that experimental conditions were matched appropriately between control and treated groups, maintaining the radioresistant characteristics where required and avoiding confounding effects from ongoing irradiation. Following these treatments, cells were gathered for subsequent functional assays. The sequences are presented in **Supplementary Table 4**.

**Quantitative real-time polymerase chain reaction (****qRT-PCR)**

The total RNA was extracted from cells using an Eastep^TM^ Total RNA Super Extraction Kit (LS1040, PROMEGA, USA). Next, RNA was reverse-transcribed into cDNA using an Eastep RT Master Mix (LS2052, PROMEGA, USA). For miRNA analysis, gene-specific stem-loop RT primers were used. According to the instructions provided with the Eastep QPCR Master Mix (2×) (LS2062, PROMEGA, USA), qRT-PCR was performed using a real-time PCR instrument (ABI 7500, ABI, USA). As reference genes for mRNA and miRNA expression, β-actin and U6 were utilized, respectively. The primers were all obtained from Sunya Biotech (China). The primer sequences are shown in **Supplementary Table 5**. The relative expression levels of the target genes between the experimental and control groups were determined using the 2^-ΔΔCt^ method.

**Western blot**

The radio immunoprecipitation assay (RIPA; PC101, EpiZyme, China) lysates containing 1% protease inhibitor cocktail (GRF101, EpiZyme, China) and phosphatase inhibitor cocktail (GRF102, EpiZyme, China) were used to extract the protein samples from either cells or exosomes. A BCA kit (ZJ101, EpiZyme, China) was used to quantify the protein. The protein samples were separated by 4-20% sodium dodecyl sulfate-polyacrylamide gel electrophoresis (SDS-PAGE; ACE, China), and the resulting separated proteins were subsequently transferred onto a polyvinylidene fluoride (PVDF) membrane (ISEQ00010, Millipore, Germany). Following incubation with a 5% skim milk solution, the PVDF membranes were then incubated with primary antibodies at 4 °C overnight. Following the overnight incubation, the membranes were incubated with horseradish peroxidase (HRP)-conjugated secondary antibodies specific to the primary antibody at room temperature for 1 h. Afterward, the protein bands were visualized using an enhanced chemiluminescence (ECL) (MA0186, Meilunbio, China) kit. Finally, protein bands were imaged using either a Bio-Rad image analysis system (1708195, USA) or an SCG-W3000 PLUS chemiluminescence imaging system (Servicebio, China), and ImageJ was used for analysis. The antibodies used for primary and secondary detection are detailed in **Supplementary Table 1**.

**Comet assay**

Cells in the logarithmic growth phase were seeded and allowed to adhere overnight. Following irradiation, cells were immediately collected for the comet assay using the Comet Assay Kit (ab238544-75T, Abcam, UK). The agarose solution was liquefied in a 90°C water bath, then maintained at 37°C. A base layer of 75 μL molten agarose was added to each well of a comet slide and solidified at 4°C for 15 min. Cells were gently scraped to avoid enzymatic digestion, centrifuged at 700 g for 2 min, washed once with cold PBS, and resuspended in cold PBS at 2 × 10⁵ cells/mL. The cell suspension was mixed with molten agarose (1:10, v/v), and 75 μL of the mixture was layered over the solidified base gel. Slides were incubated at 4°C for 15 min in the dark to solidify. After solidification, slides were immersed in freshly prepared lysis buffer and incubated overnight at 4°C in the dark. Slides were then transferred to pre-chilled alkaline solution for 30 min, followed by two 5-min washes in neutral electrophoresis buffer. Electrophoresis was performed in neutral buffer at 1 V/cm, with current maintained at ~300 mA for 20 min. Slides were rinsed in cold deionized water (three times, 2 min each) and fixed in cold 70% ethanol for 5 min. After air-drying, 100 μL of 1× Vista Green DNA dye (diluted 1:10,000 in TE buffer) was added to each well, and slides were incubated for 15 min at room temperature in the dark. DNA damage was visualized under a fluorescence microscope using the FITC channel and quantified using CASP software.

**Immunofluorescence staining**

Cells were seeded onto confocal culture dishes and incubated at 37°C in a humidified atmosphere with 5% CO₂. After cell adherence, different treatment conditions were applied as required. Cells were then washed twice with PBS and fixed with 4% paraformaldehyde (BL539A, Biosharp, China) for 20 min at room temperature. Following fixation, cells were washed three times with immunofluorescence washing buffer (P0106, Beyotime, China) (5 min each). Permeabilization was performed using a permeabilization buffer (P0097, Beyotime, China) for 15 min, followed by three additional washes. Cells were blocked with immunofluorescence blocking solution (P0102, Beyotime, China) for 30 min at room temperature. Cells were incubated with primary antibodies overnight at 4°C using antibody dilution buffer according to the manufacturer’s instructions. After incubation, cells were washed three times and then incubated with a fluorophore-conjugated secondary antibody at room temperature in the dark for 1 h. Cells were again washed three times and mounted with an antifade mounting medium containing DAPI. Images were captured using a laser scanning confocal microscope (ZEISS, Germany) under dark conditions, and fluorescence intensity was analyzed using ImageJ software. Details of the antibodies employed for primary and secondary detection are presented in **Supplementary Table 1**.

**Colony formation assay**

Cell proliferation was assessed using the colony formation assay. Cells were seeded evenly into six-well plates and the liquid culture medium was replaced every 2-3 days. Following a two-week incubation period, the colonies were fixed with 4% paraformaldehyde (BL539A, Biosharp, China). Subsequently, the colonies were stained with crystal violet (C0121, Beyotime, China).

**Cell viability assay**

Cell viability was evaluated using a cell counting kit-8 (CCK-8; ABK0011C-5ml, ABKBio, China) assay. Cells were treated according to the corresponding protocols and subsequently seeded into 96-well plates. Subsequently, 10 μL of CCK-8 reagent was added to each well, and the plates were incubated at 37°C for 2 h. Following this, a microplate reader was utilized to measure the absorbance of each well at 450 nm.

**Cell apoptosis assay**

Cell apoptosis was evaluated using an Annexin V-APC/7-AAD Apoptosis kit (E-CK-A218, Elabscience, China) according to the manufacturer's instructions. Briefly, cells were induced to undergo apoptosis according to the experimental protocol. Following induction, cells were collected by centrifugation at 300 g for 5 min, washed once with PBS, and gently resuspended for counting. A total of 1-5 × 10^5^ cells were centrifuged at 300 g for 5 min, washed with PBS, and resuspended in 100 μL of 1× Annexin V Binding Buffer. Subsequently, 2.5 μL of Annexin V-APC reagent and 2.5 μL of 7-AAD reagent (100 μg/mL) were added to the cell suspension, followed by gentle vortexing and incubation at room temperature in the dark for 15-20 min. After incubation, 400 μL of 1× Annexin V Binding Buffer was added, mixed gently, and samples were immediately analyzed by flow cytometry. Annexin V-APC and 7-AAD signals were detected using APC and PerCP/Cy5.5 channels, respectively.

**Cell cycle assay**

A cell cycle analysis kit (CA1510-20T, Solarbio, China) was used to track the cell cycle distribution of cells at various phases of the cell cycle. To analyze cell cycle progression, cells were fixed overnight at 4°C using 70% ice-cold ethanol. On the following day, cells were treated with RNase A and propidium iodide (PI) for 30 min. Finally, cell cycle distribution was assessed using a flow cytometer (B49009AD, Beckman, USA) and CytExpert or Flowjo software with 488 nm excitation.

**Transcriptome sequencing**

Total RNA was extracted from tissues or cells and assessed for quality and integrity using an Agilent 2100 Bioanalyzer. mRNA was enriched either by poly(A) selection using Oligo(dT) magnetic beads or by rRNA depletion. The purified mRNA was fragmented with divalent cations and used for library preparation via either conventional or strand-specific protocols. Constructed libraries were first quantified using a Qubit 2.0 and assessed for insert size using an Agilent 2100 Bioanalyzer. qPCR was then used to determine library concentration (≥ 1.5 nM). Qualified libraries were pooled and sequenced on an Illumina platform using the sequencing-by-synthesis method. Raw image data were processed using CASAVA for base calling, generating FASTQ files. Low-quality reads were filtered to obtain clean reads, which were aligned to the reference genome using HISAT2. Read counts per gene were calculated based on genome annotation to enable downstream expression analysis.

**Proteomic sample preparation and mass spectrometry**

Frozen tissue samples (stored at -80°C) were lysed in buffer containing 8 mol/L urea (U5378, Sigma-Aldrich, USA), 1% protease inhibitor (Merck Millipore, USA), 3 μmol/L Trichostatin A (58880-19-6, MedChemExpress, USA), and 50 mmol/L Nicotinamide (72340, Sigma-Aldrich, USA). After sonication and centrifugation (12,000 g, 10 min, 4°C), supernatants were collected, and protein concentrations were determined by a BCA (P0012, Beyotime, China) assay. Equal protein amounts were precipitated with 20% (v/v) trichloroacetic acid (T0699, Sigma-Aldrich, USA) at 4°C for 2 h, centrifuged (4,500 g, 5 min), washed with cold acetone, and resuspended in 200 mmol/L tetraethylammonium bromide (241059, Sigma-Aldrich, USA). Proteins were digested with trypsin (V5280, Promega, USA) (1:50, w/w) overnight, reduced with 5 mmol/L DL-Dithiothreitol (D9779, Sigma-Aldrich, USA) (56°C, 30 min), and alkylated with 11 mmol/L iodoacetamide (I6125, Sigma-Aldrich, USA) (dark, RT, 15 min). Peptides were separated by UHPLC (Vanquish Neo) using a 30-min gradient with 0.1% formic acid in water (mobile phase A) and 80% acetonitrile with 0.1% formic acid (mobile phase B), at a flow rate of 400 nL/min. Separated peptides were analyzed on a timsTOF HT mass spectrometer in dia-PASEF mode with an MS1 range of 300-1,500 m/z and an MS2 range of 380-1,170 m/z (10 m/z windows).

**Molecular docking analysis**

The crystal structures of EFHD2 (PDB ID: 5I2L) and HMGB1 (PDB ID: 6CJK) were retrieved from the Protein Data Bank. Protein-protein docking was performed using HDOCK to investigate the potential interaction between EFHD2 and HMGB1. The resulting docking complexes were visualized and analyzed for interaction interfaces using PyMOL 2.3.0.

**Animal models**

Female NOD-SCID immunodeficient mice and BALB/c mice (3-5 weeks old) were obtained from Wu’s Experimental Animal Center. All animals were housed under specific pathogen-free (SPF) conditions in SPF-grade isolation facilities, with protocols approved by the Institutional Animal Care and Use Committee (Approval No. IACUC FJMU 2024-Y-2147). The maximum allowable tumor volume was 2,000 mm³, and euthanasia was performed when this threshold was reached. No statistical methods were used to predetermine sample size. Each experimental group included 5-6 mice. Sample sizes were selected based on previous literature, prior experimental experience, and commonly accepted standards in the field while minimizing animal use in accordance with the 3R principles (reduction, refinement, and replacement). No pre-established exclusion criteria were applied for animal experiments, and no animals were excluded from the final analysis. Animals were randomly assigned to experimental groups after tumor inoculation. Investigators were not blinded to group allocation during the experiments or outcome assessment. Equal numbers of CT26 cells transfected with NC or shEFHD2 were subcutaneously injected into the distal hindlimb of 3-5 week-old BALB/c mice to establish a subcutaneous tumor model, with no exposure to ionizing radiation throughout, in order to assess the intrinsic tumorigenic potential of the cells. To validate the consistency of this finding in human cells, the same approach was applied using HCT116 cells stably transfected with NC or shEFHD2, which were subcutaneously injected into NOD-SCID mice and similarly observed under non-irradiated conditions. BALB/c mice were inoculated with CT26 cells in the hind limb. On days 7, 9, and 11 post-inoculation, the treatment group received 200 µg of BAY-11-7082 (HY-13453, MedChemExpress, USA) via oral gavage, while controls were given an equal volume of corn oil (HY-Y1888, MedChemExpress, USA). For sodium lactate (NALA; HY-B2227B, MedChemExpress, USA) treatment, mice were intraperitoneally injected with 120 mg/kg once daily. For LDH inhibitor (LDHi; HY-100681, MedChemExpress, USA) treatment, mice were randomized into six groups: vehicle control (PBS), EFHD2 knockdown (PBS), EFHD2 knockdown + LDHi (1,000 mg/kg), irradiation (IR) (PBS), IR + EFHD2 knockdown (PBS), and IR + EFHD2 knockdown + LDHi (1,000 mg/kg). Treatments were administered intraperitoneally three times per week. For immune checkpoint inhibition (ICI) therapy, five groups were established. Starting on day 5 post-tumor inoculation, mice received anti-PD-L1 (HY-P99145, clone: 10F.9G2, MedChemExpress, USA) and anti-CTLA-4 (BE0032, clone: UC10-4F10-11, BioXCell, USA) intraperitoneally every three days at a total dose of 600 µg per antibody. Mice received localized irradiation targeting only the tumor site at 8 Gy per fraction for three consecutive days, deliberately avoiding exposure of the spinal cord and major hematopoietic organs. This strategy was employed to minimize systemic toxicity and prevent hematopoietic failure, which can occur with whole-body or extensive-field irradiation, thereby ensuring that the animals could safely tolerate the regimen and that tumor growth could be accurately monitored until the defined endpoint.

**H&E staining**

Paraffin-embedded tissue sections were sequentially deparaffinized in environmentally friendly dewaxing solution I and II (20 min each), followed by rehydration through 100% ethanol (5 min ×2), 75% ethanol (5 min), and rinsing in tap water. Sections were treated with high-definition pre-staining solution for 1 min and stained with hematoxylin for 3-5 min. After washing in tap water, slides were differentiated, blued, and rinsed. Sections were then dehydrated in 95% ethanol (1 min), counterstained with eosin for 15 s, and dehydrated through a graded series of ethanol (100% ×3, 2 min each), followed by butanol I and II (2 min each), and cleared in xylene I and II (2 min each). Finally, slides were mounted with neutral resin. Images were captured and analyzed under a light microscope.

**Immunohistochemistry (IHC) staining**

Paraffin-embedded tissue sections were deparaffinized in environmentally friendly dewaxing solution I-III (10 min each), followed by rehydration through graded ethanol (100% ×3, 5 min each) and rinsed in distilled water. Antigen retrieval was performed using EDTA buffer (pH 9.0) under microwave heating (medium power for 8 min, pause for 8 min, then low-medium power for 7 min), followed by natural cooling and three PBS washes (5 min each) on a shaker. Endogenous peroxidase activity was blocked with 3% H₂O₂ for 25 min at room temperature in the dark, followed by PBS washes. Non-specific binding was blocked using 3% BSA (or rabbit serum if the primary antibody was goat-derived) for 30 min at room temperature. After discarding the blocking buffer, slides were incubated with appropriately diluted primary antibodies overnight at 4°C in a humidified chamber. Slides were then washed in PBS (3×, 5 min), incubated with HRP-conjugated secondary antibodies for 50 min at room temperature, and washed again. DAB substrate was applied, and signal development was monitored under a light microscope until brown staining appeared. Staining was stopped with tap water. Sections were counterstained with hematoxylin (~3 min), differentiated, blued, rinsed, and dehydrated through an ethanol gradient (75%, 85%, 100% ×2), followed by butanol and xylene (5 min each). Slides were air-dried briefly and mounted with neutral resin. Stained sections were evaluated under a bright-field microscope.

**Determination of intracellular lactate levels and glucose consumption rate**

Intracellular lactate levels were quantified using a lactic acid assay kit (A019-2-1, Jiancheng Corporation, China) in accordance with the manufacturer’s instructions. The glucose consumption rate was measured using a glucose content assay kit (BC2500, Solarbio, China) following the manufacturer's instructions.

**Treatment conditions for protein expression, immunofluorescence, and colony formation assays**

For protein expression analysis by Western blotting, cells were treated with LDHi at concentrations of 10 mM or 20 mM, NALA at 25 mM or 50 mM, and BAY-11-7082 at 5 μM. In the case of immunofluorescence assays, cells were exposed to 10 mM LDHi and 25 mM NALA. For colony formation assays, cells were treated with 10 mM LDHi, 10 mM NALA, and 5 μM BAY-11-7082. Additionally, immune checkpoint blockade antibodies, including anti-PD-L1 and anti-CTLA-4, were applied at a concentration of 10 μg/mL. These treatment regimens were selected to assess the effects of metabolic inhibition and immune checkpoint modulation across different experimental platforms.

**Lactylation proteomics analysis**

Samples were lysed in a buffer (8 mol/L urea (U5378, Sigma-Aldrich, USA), 1% protease inhibitor (Merck Millipore, USA), 3 μmol/L Trichostatin A (58880-19-6, MedChemExpress, USA), and 50 mmol/L nicotinamide (72340, Sigma-Aldrich, USA)), sonicated, and centrifuged (12,000 g, 10 min, 4°C). Protein concentration was measured using a BCA kit (P0012, Beyotime, China). Equal protein amounts were precipitated with 20% trichloroacetic acid (T0699, Sigma-Aldrich, USA) at 4°C for 2 h, washed with cold acetone, and redissolved in 200 mM tetraethylammonium bromide (241059, Sigma-Aldrich, USA). Proteins were digested overnight with trypsin (V5280, Promega, USA) (1:50, w/w), reduced with 5 mM DL-Dithiothreitol (D9779, Sigma-Aldrich, USA) (30 min, 56°C), and alkylated with 11 mM iodoacetamide (I6125, Sigma-Aldrich, USA) (15 min, RT, dark). Peptides were dissolved in IP buffer (100 mM NaCl, 1 mM EDTA, 50 mM Tris-HCl, 0.5% NP-40, pH 8.0) and incubated overnight at 4°C with pan-anti-lactyllysine antibody-conjugated agarose beads on a rotator. The beads were washed four times with IP buffer and twice with distilled water. Lactylated peptides were eluted three times with 0.1% trifluoroacetic acid (TFA), pooled, and vacuum-dried. The eluates were desalted using C18 ZipTips (Millipore, Burlington, MA, USA) and dried for LC-MS/MS analysis. Peptides were separated by UHPLC (Vanquish Neo) using a gradient of 8-99% solvent B (0.1% formic acid in 80% acetonitrile) and analyzed on a timsTOF HT mass spectrometer in dia-PASEF mode (with an MS1 range of 100-1,700 m/z and an MS2 range of 425-1,025 m/z, 25 m/z windows).

**Flow cytometry analysis**

Tumor tissues were collected, washed once with pre-chilled PBS, and dissociated mechanically using a 100 μm cell strainer moistened with cold 5% FBS-PBS. After adding 5% FBS-PBS to a final volume of 10 mL, samples were centrifuged at 500 g for 8 min, and the supernatant was discarded. The pellet was resuspended in a red blood cell lysis buffer and incubated on ice for 5 min. Lysis was stopped by adding 10 mL of 5% FBS-PBS, followed by centrifugation at 500 g for 8 min. Cells were resuspended in 5% FBS-PBS and adjusted to a concentration of 1 × 10⁷ cells/mL. For surface staining, FITC hamster anti-mouse CD3e (553062, BD Biosciences, USA), APC rat anti-mouse CD4 (553051, BD Biosciences, USA), PE-Cy7 rat anti-mouse CD8a (561097, BD Biosciences, USA), and BV421 rat anti-mouse CD25 (562606, BD Biosciences, USA) antibodies were added and incubated for 30 min at 4°C in the dark. For intracellular Foxp3 staining, cells were fixed with 1 mL of 1× fixation buffer and incubated for 3 h at room temperature in the dark. Without washing, 2 mL of 1× permeabilization buffer was added, followed by centrifugation at 300-400 g for 5 min at room temperature. Cells were washed twice with permeabilization buffer and resuspended in 100 μL of the same buffer. Then, PE rat anti-mouse Foxp3 antibody (563101, BD Biosciences, USA) was added and incubated for 30 min at room temperature in the dark. After two washes, cells were resuspended in 500 μL of cell staining buffer and analyzed by flow cytometry.

**Spatial transcriptomics analysis**

Nine clinical specimens from RC patients who underwent curative surgery at our institution were collected. Immunohistochemical staining was first performed to evaluate EFHD2 expression, based on which samples were divided into EFHD2-high and EFHD2-low expression groups. All samples were processed into tissue microarrays and subjected to spatial transcriptomic profiling using the 10x Genomics Visium HD platform. Gene-specific probe pairs (LHS and RHS) designed for ~18,000 human genes hybridized to target RNA sequences in the tissue. The LHS contains a Read 2 sequence for PCR amplification, while the RHS has a poly-A tail for capture by slide-bound oligos. After hybridization and ligation, the RNA strand was degraded and ligated probes were released, extended, and reverse transcribed to generate cDNA. The cDNA was recovered, and sequencing libraries were prepared and quantified using a Qubit 3.0 fluorometer. Libraries passing quality control were sequenced on the Illumina NovaSeq™ 6000 platform (PE150 strategy). Raw sequencing data were processed using Space Ranger (version 3.1), aligned to the human GRCh38 reference genome, and UMI counts were obtained. Spatial barcodes corresponding to tissue-covered areas were retained to generate filtered gene expression matrices. Low-quality spots were excluded based on the following criteria: fewer than 50 detected genes, > 30% mitochondrial gene content, or genes detected in < 1% of all spots. Differential gene expression analysis was conducted between the EFHD2-high and EFHD2-low expression groups, using the thresholds of |log₂ fold change| > 1 and an adjusted *p*-value < 0.05 to define statistical significance. Identified DEGs were categorized as upregulated (log₂FC > 1) or downregulated (log₂FC < -1). GSEA was further conducted based on these DEGs to explore associated functional pathways.

**Single-cell RNA sequencing (****scRNA-seq)**

**scRNA-seq data acquisition and preprocessing**

Twelve publicly available scRNA-seq datasets involving both normal colorectal tissues and colorectal cancer tissues were obtained from the GEO database (GSE161277, GSE163974, GSE188711, GSE205506, GSE217774, GSE222300, GSE225857, GSE231559, GSE232525, GSE277669, GSE277905, and GSE282542)^22, 23, 24, 25, 26, 27, 28, 29, 30, 31, 32^ (https://www.ncbi.nlm.nih.gov/geo/query/acc.cgi?acc=217774 and https://www.ncbi.nlm.nih.gov/geo/query/acc.cgi?acc=282542). These datasets comprised a total of 108 samples and over 600,000 cells. Data preprocessing, quality control, normalization, and feature selection were performed using the Seurat (version 5) package in R^33^. Low-quality cells and potential doublets were excluded.

**Batch effect correction and clustering**

To eliminate batch effects across multiple datasets, the Harmony algorithm was applied following standard normalization. After integration, principal component analysis (PCA) was conducted for dimensionality reduction, followed by Uniform Manifold Approximation and Projection (UMAP) for visualization.

**Cell type annotation**

Marker genes were identified for each cluster using the FindAllMarkers function in Seurat. Cell types were annotated based on canonical markers and classified into major lineages, including T cells, myeloid cells, epithelial cells, endothelial cells, fibroblasts, B cells, plasma cells, and proliferative cells.

**EFHD2 expression-based grouping**

Cells were grouped into EFHD2-positive and EFHD2-negative populations according to EFHD2 expression levels. The relative proportions of different cell types within each group were quantified and compared. Differential distribution patterns were used to infer potential associations between EFHD2 expression and the cellular composition of the tumor microenvironment.

**Myeloid cells subclustering**

Myeloid cells were extracted and re-clustered to identify finer subpopulations. Marker-based annotation was conducted to define distinct subtypes, including Macro_C1QC, M2, Macro-NUPR1, Mono-S100A8, Neutrophil, Macro-SELENOP, cDC2-CD1A, MAST-TPSAB1, Activated-DC, M1, Macro-FN1, and pDC-LILRA4.

**Macrophage polarization scoring and visualization**

To investigate macrophage polarization states within the tumor microenvironment, we performed module scoring of M1 and M2 polarization signatures using the AddModuleScore function in Seurat^33^. A curated list of genes associated with M1 and M2 phenotypes was compiled based on published literature. These gene sets were used to calculate polarization scores for each cell, denoted as M1_Score and M2_Score, respectively. In parallel, cells were stratified into EFHD2-positive and EFHD2-negative groups based on the expression level of EFHD2, with a threshold of zero used to define positivity. This stratification allowed comparative analysis of macrophage polarization profiles across different EFHD2 expression states. For downstream analysis and visualization, each cell was annotated with its corresponding cell type, based on cluster identity and known marker gene expression. Scatter plots were generated to compare M1 and M2 scores across various cell types and EFHD2 expression groups. Furthermore, faceted plots and linear trend lines were used to illustrate polarization tendencies and potential associations with EFHD2 expression.

**Pseudotime trajectory analysis of myeloid subpopulations**

To investigate the potential differentiation trajectories within the myeloid cell compartment, pseudotime analysis was performed using the Monocle package^34^. Myeloid subpopulations identified through re-clustering in Seurat were imported into Monocle. The top variable genes were selected to construct a trajectory graph using the learn_graph function. Cells were ordered in pseudotime with the order_cells function, and the root node was selected based on the presumed progenitor-like cell state. The inferred trajectories were visualized to explore the dynamic transitions and lineage relationships among myeloid subtypes.

**Cell-cell communication analysis**

Cell-cell communication was inferred using the CellChat R package^35^. Interaction networks and signaling pathways were constructed for EFHD2-positive and EFHD2-negative groups independently. Differentially active signaling pathways between groups were identified, and key ligand–receptor pairs contributing to communication between immune cell subsets were characterized.

**T-cell-mediated colony formation assay**

Peripheral blood mononuclear cells (PBMCs) were obtained from a commercial supplier (TPCS, China) and activated *in vitro* with IL-2 (STEMCELL, Canada) and ImmunoCult Human CD3/CD28/CD2 T Cell Activator (STEMCELL, Canada) following the manufacturer’s protocol. Radioresistant cells were plated at 1,500 cells per well in 6-well plates. After overnight attachment, cells were exposed to ionizing radiation at the designated dose. Activated PBMCs were collected by centrifugation at 200 g for 10 min, resuspended in fresh medium, and added to each well to achieve a 5:1 effector-to-target ratio relative to the radioresistant cells. For groups requiring immune-checkpoint blockade, the co-culture system was supplemented with anti-CTLA-4 and/or anti-PD-L1 antibodies at the working concentrations recommended by the manufacturers. After 24 h of co-culture, the medium containing PBMCs and antibodies was removed, and wells were gently rinsed with PBS to eliminate non-adherent immune cells. The remaining radioresistant cells were then cultured in complete medium for 10-14 days to allow colony formation. Colonies were fixed with 4% paraformaldehyde for 15 min, stained with 0.5% crystal violet for 20 min, washed with distilled water, and air-dried. Colonies were counted under a light microscope.

**Supplementary Figures**

**Supplementary Figure 1 Characterization of exosomes and identification of hsa-let-7b-5p as a radiosensitivity-related miRNA.** (A) Transmission electron microscopy of exosomes isolated from SW480 and SW480R cells. (B-C) Nanoparticle tracking analysis showing the size distribution and concentration of exosomes. (D) Volcano plot of differentially expressed exosomal miRNAs between SW480 and SW480R cells. (E) Heatmap showing 74 differentially expressed exosomal miRNAs between SW480 and SW480R cells, including 45 upregulated and 29 downregulated miRNAs. (F-H) Functional enrichment analysis indicating the involvement of these miRNAs in metabolic processes and DNA binding. (I-J) Reduced hsa-let-7b-5p expression in colorectal cancer tissues compared to normal tissues. (K) Pan-cancer analysis showing broadly decreased hsa-let-7b-5p expression. (L) Low hsa-let-7b-5p levels are associated with a poorer prognosis. (M) Overexpression of hsa-let-7b-5p does not affect cell proliferation under basal conditions. (N-O) CCK-8 assays showing that hsa-let-7b-5p overexpression significantly reduces the proliferation of HCT116R and SW480R cells following ionizing radiation.

**Supplementary Figure 2 hsa-let-7b-5p modulates** **the cell cycle, apoptosis, DNA damage, and targets EFHD2.** (A) Overexpression of hsa-let-7b-5p reduced the S-phase accumulation following irradiation. (B) Increased apoptosis was observed in hsa-let-7b-5p-overexpressing cells. (C-G) EFHD2 expression is elevated in radioresistant colorectal cancer tissues and in tumor tissues compared with normal tissues, as validated using external GEO datasets. (H-I) Dual-luciferase reporter assays confirm direct binding of hsa-let-7b-5p to the EFHD2 3′UTR.

**Supplementary Figure 3 EFHD2 regulates radiation-induced cell proliferation, cell cycle** **arrest, and apoptosis.** (A-B) Under non-irradiated conditions, EFHD2 knockdown or overexpression has no significant effect on cell proliferation. (C-D) EFHD2 knockdown decreases, whereas overexpression increases, cell proliferation following irradiation. (E) EFHD2 overexpression increases S-phase arrest in parental cells. (F-G) EFHD2 knockdown enhances, whereas overexpression reduces, radiation-induced apoptosis. (H) Colony formation assays confirm EFHD2-mediated modulation of cell proliferation following irradiation.

**Supplementary Figure 4 EFHD2 regulates the DNA damage response and transcriptomic reprogramming.** (A) The comet assay shows enhanced DNA damage upon EFHD2 knockdown after irradiation. (B) Immunofluorescence analysis indicates increased γH2AX foci in EFHD2 knockdown resistant cells. (C-E) Transcriptomic profiling of EFHD2-knockdown cells and NC cells. (F-G) EFHD2 knockdown affects chromatin/replication complexes and DNA-binding/enzyme activity. (H-M) Proteomic analysis supports the involvement of EFHD2 in the radiotherapy response, metabolism, and immune regulation.

**Supplementary Figure 5 EFHD2 regulates HIF-1α and lactate metabolism.** (A-H) Correlation analysis between HMGB1 and lactate metabolism-related genes. (I-O) Correlation analysis between HIF-1α and lactate metabolism-related genes.

**Supplementary Figure 6 Functional effects of lactate metabolism and EFHD2 lactylation in DNA damage and repair.** (A) Immunofluorescence analysis showing the regulation of γH2AX levels by NALA and LDHi. (B-C) Lactate production and glucose consumption assays. (D) Protein pan-lactylation changes upon NALA or LDHi treatment. (E) Lactyl-proteomic profiling identifying differentially modified sites. (F-I) Enrichment analysis of lactylated proteins. (J) The top 30 differentially lactylated sites in control versus EFHD2-knockdown cells. (K) The comet assay demonstrating enhanced DNA damage in EFHD2-K9 mutant cells. (L-M) Regulation of RAD51 and RPA2 levels by NALA and LDHi. (N-O) Immunohistochemistry analysis of human rectal cancer tissues and mouse xenografts showing that EFHD2 expression positively correlates with RAD51 and p-RPA2 levels, and inversely with γH2AX levels.

**Supplementary Figure 7 Single-cell RNA-seq analysis of EFHD2 expression and its association with M2 macrophage polarization.** (A) UMAP clustering of 108 single-cell RNA-seq samples integrated using the Harmony algorithm, identifying nine cell clusters. (B) Marker gene-based annotation of clusters, including T cells, myeloid cells, endothelial cells, fibroblasts, B cells, plasma cells, epithelial cells, and proliferative cells. (C) Distribution of cell clusters across all samples. (D-E) EFHD2 expression is enriched in myeloid, T, and epithelial cells, with higher levels in colorectal cancer tissues compared to normal tissues. (F) Classification of cells into EFHD2-positive and EFHD2-negative groups. (G) Cell-type distribution showing increased proportions of myeloid, proliferative, and epithelial cells and decreased proportions of B, plasma, and T cells in the EFHD2-positive group. (H) A higher proportion of EFHD2-positive cells is observed in tumor tissues relative to normal tissues. (I-J) Subclustering of myeloid cells into 12 subsets based on marker expression. (K) Distribution of myeloid subsets by EFHD2 expression. (L) The EFHD2-positive group exhibits higher proportions of M2, Macro-NUPR1, and Mono-S100A8 cells, and lower proportions of Activated-DC, Macro_C1QC, Macro-SELENOP, MAST-TPSAB1, and neutrophils. (M-Q) Single-cell level analyses showing that M2 polarization scores positively correlate with EFHD2 expression.

**Supplementary Figure 8 Pseudotime and cell-cell communication analyses reveal EFHD2-associated immunosuppressive phenotypes.** (A-C) Pseudotime trajectory analysis showing a developmental progression from immune-activating to immunosuppressive phenotypes, with EFHD2-positive cells enriched at the terminal, immunosuppressive states. (D-P) Cell-cell communication analysis demonstrating enhanced interactions in the EFHD2-positive group, particularly among M2 macrophages and other cell types, with increased signaling through immunosuppressive and extracellular matrix remodeling pathways including TGF-β, IL-10, IL-6, Tenascin, and SPP1.

**Supplementary Figure 9 Correlation of EFHD2 with M2 macrophage polarization.** (A-R) IHC analysis and quantification of EFHD2, HMGB1, CD68, HLA-DR, and CD163 in the tumor parenchyma and stroma. (S-T) GEO dataset validation of the correlation between EFHD2 and M2 markers.

**Supplementary Figure 10 EFHD2 shapes an immunosuppressive tumor microenvironment.** (A) Western blot analysis showing that NALA treatment promotes CD163 expression, whereas LDHi treatment suppresses CD163 expression. (B-I) High expression of EFHD2 showed increased Treg infiltration and decreased infiltration of B cells, dendritic cells, CD4⁺ and CD8⁺ T cells, along with lower immune scores compared to EFHD2-low tumors.

**Supplementary Figure 11 Spatial transcriptomic and histological characterization of EFHD2 in rectal cancer.** (A) Spatial transcriptomics profiling of nine rectal cancer samples. (B) Quality control of the spatial transcriptomic data. (C-I) The spatial distribution of EFHD2 and M2 macrophage-associated markers expression (MSR1, PPARG, STAT6, TGFB1, CCL18, CCL22, CCL24). (J-K) GSEA showing the enrichment of DNA replication (chemoresistance) and NF-κB signaling pathways in EFHD2-high tumors. (L-M) Immunohistochemistry analysis of human rectal cancer tissues and mouse tumors demonstrating that EFHD2-high samples exhibit reduced CD4⁺ and CD8⁺ T cell infiltration and increased FOXP3⁺ cell infiltration and PD-L1 expression. (N-O) Immunofluorescence analysis confirming that EFHD2 knockdown reduces, whereas overexpression promotes, NF-κB p65 nuclear translocation.

**Supplementary Figure 12 EFHD2 expression correlates with radioresistance, lactate metabolism, and immune modulation in clinical rectal cancer samples.** (A-D) The expression levels of PKM and HK1 are higher in radioresistant tumors compared with radiosensitive tumors. (E) Correlation analysis showing that EFHD2 expression is positively associated with LDHA, PKM, and HK1 expression, and negatively associated with CD8A and CD4 expression. (F) Analysis of immune-related genes reveals their decreased expression in EFHD2-high tumors. (G) Gene enrichment analysis of differentially expressed genes between EFHD2-high and EFHD2-low groups highlights enrichment in pathways related to double-strand break repair, DNA recombination, homologous recombination repair, and DNA replication.

**Supplementary Tables**

**Supplementary Table 1 Primary and secondary antibodies used in this study.**

| Antibody | Dilutions for WB | Dilutions for IHC | Dilutions for IF | Company |
| --- | --- | --- | --- | --- |
| β-actin | 1:10000 | / | / | ABclonal (AC050) |
| γH2AX | 1:7500 | / | 1:250 | Abcam (ab81299) |
| γH2AX | / | 1:100 | / | Servicebio (GB111841) |
| EFHD2 | 1:6000 | / | / | Proteintech (83264-5-RR) |
| EFHD2 | / | 1:100 | / | Thermo (PA5-61846) |
| Bax | 1:1000 | / | / | CST (14796) |
| Bcl-2 | 1:1000 | / | / | CST (3498) |
| Caspase-3 | 1:1000 | / | / | CST (14220) |
| cleaved Caspase-3 | 1:1000 | / | / | CST (9664) |
| ATM | 1:1000 | / | / | HUABIO (ET1606-20) |
| p-ATM | 1:1000 | / | / | HUABIO (ET1705-50) |
| HMGB1 | 1:1000 | 1:250 | / | CST (6893) |
| HMGB1 | 1:10000 | / | / | Proteintech (10829-1-AP) |
| lKKα | 1:1000 | / | / | CST (11930) |
| IKKβ | 1:1000 | / | / | CST (8943) |
| Phospho-IKKa/β | 1:1000 | / | / | CST (2697) |
| Phospho-NF-kB p65 | 1:1000 | / | / | CST (3033) |
| IkBa | 1:1000 | / | / | CST (4814) |
| Phospho-IkBa | 1:1000 | / | / | CST (2859) |
| NF-KB p65 | 1:1000 | / | 1:600 | CST (8242) |
| PD-L1 | 1:6000 | / | 1:3000 | Proteintech (66248-1-Ig) |
| PD-L1 | / | 1:200 | / | Servicebio (GB155736) |
| CD68 | / | 1:400 | / | ZSGB-BIO (ZM-0060) |
| CD163 | / | 1:200 | / | ZSGB-BIO (ZM-0428) |
| HLA-DR | / | 1:200 | / | HUABIO (ET1610-66) |
| PANCK | / | 1:2000 | / | HUABIO (HA601138) |
| HIF-1α | 1:5000 | / | / | HUABIO (HA721997) |
| CD163 | 1:1500 | / | / | Proteintech (16646-1-AP) |
| RAD51 | / | 1:250 | 1:100 | HUABIO (ET1705-96) |
| RAD51 | / | 1:500 | / | Servicebio (GB11573) |
| Anti-L-Lactyl Lysine | 1:750 | / | / | PTM Biolabs (PTM-1401RM) |
| Anti-lactyl-EFHD2 (K9) Rabbit pAb | 1:500 | / | / | PTM Biolabs (PTM- CO1208) |
| RPA2 | 1:3000 | / | 1:250 | Proteintech (10412-1-Ap) |
| p-RPA2 | 1:6000 | 1:200 | / | BETHYL (A300-245A) |
| CD4 | / | 1:100 | / | Servicebio (GB13588) |
| CD4 | / | 1:500 | / | Servicebio (GB15064) |
| CD8 | / | 1:1000 | / | Servicebio (GB12068) |
| CD8 | / | 1:500 | / | Servicebio (GB15068) |
| FOXP3 | / | 1:200 |  | Servicebio (GB112325) |
| Foxp3 | / | 1:1000 | / | Servicebio (GB115746) |
| HRP Goat Anti-Rabbit IgG (H+L) | 1:5000 | / | / | ABclonal (AS014) |
| HRP Goat Anti-Mouse IgG (H+L) | 1:5000 | / | / | ABclonal (AS003) |
| Cy3 Goat Anti-Rabbit IgG (H+L) | / | / | 1:500 | ABclonal (AS007) |
| ABflo® 647-conjugated Goat Anti-Mouse (H+L) | / | / | 1:500 | ABclonal (AS059) |
| HRP-conjugated goat anti-rabbit IgG | / | 1:200 | / | Servicebio (GB23303) |
| HRP-conjugated goat anti-mouse IgG | / | 1:200 | / | Servicebio (GB23301) |

**Supplementary Table 2 EFHD2 plasmids with WT and lactylation site mutations.**

| Name | Sequence |
| --- | --- |
| EFHD2-WT | Atggccacggacgagctggccaccaagctgagccggcggctgcagatggagggcgagggcggcggcgagaccccggagcagcccgggctgaacggggcagcggcggcggcggcgggggcacccgacgaggcggccgaggcgctgggcagcgcggactgcgagctgagcgccaagctgctgcggcgcgcagacctcaaccagggcatcggcgagccccagtcgcccagccgccgcgtcttcaacccctacaccgagttcaaggagttctccaggaagcagatcaaggacatggagaagatgttcaagcagtatgatgccgggcgggacggcttcatcgacctgatggagctaaaactcatgatggagaaacttggggcccctcagacccacctgggcctgaaaaacatgatcaaggaggtggatgaggactttgacagcaagctgagcttccgggagttcctcctgatcttccgcaaggcggcggccggggagcttcaggaggacagcgggctgtgcgtgctggcccgcctctctgagatcgacgtctccagtgagggtgtcaagggggccaagagcttctttgaggccaaggtccaggccatcaacgtgtccagccgcttcgaggaggagatcaaggcagagcaggaggaaaggaagaagcaggcggaggagatgaagcagcggaaagcggccttcaaggagctgcagtccacctttaagtag  MATDELATKLSRRLQMEGEGGGETPEQPGLNGAAAAAAGAPDEAAEALGSADCELSAKLLRRADLNQGIGEPQSPSRRVFNPYTEFKEFSRKQIKDMEKMFKQYDAGRDGFIDLMELKLMMEKLGAPQTHLGLKNMIKEVDEDFDSKLSFREFLLIFRKAAAGELQEDSGLCVLARLSEIDVSSEGVKGAKSFFEAKVQAINVSSRFEEEIKAEQEERKKQAEEMKQRKAAFKELQSTFK* |
| EFHD2-K9E | Atggccacggacgagctggccaccgagctgagccggcggctgcagatggagggcgagggcggcggcgagaccccggagcagcccgggctgaacggggcagcggcggcggcggcgggggcacccgacgaggcggccgaggcgctgggcagcgcggactgcgagctgagcgccaagctgctgcggcgcgcagacctcaaccagggcatcggcgagccccagtcgcccagccgccgcgtcttcaacccctacaccgagttcaaggagttctccaggaagcagatcaaggacatggagaagatgttcaagcagtatgatgccgggcgggacggcttcatcgacctgatggagctaaaactcatgatggagaaacttggggcccctcagacccacctgggcctgaaaaacatgatcaaggaggtggatgaggactttgacagcaagctgagcttccgggagttcctcctgatcttccgcaaggcggcggccggggagcttcaggaggacagcgggctgtgcgtgctggcccgcctctctgagatcgacgtctccagtgagggtgtcaagggggccaagagcttctttgaggccaaggtccaggccatcaacgtgtccagccgcttcgaggaggagatcaaggcagagcaggaggaaaggaagaagcaggcggaggagatgaagcagcggaaagcggccttcaaggagctgcagtccacctttaagtag  MATDELATELSRRLQMEGEGGGETPEQPGLNGAAAAAAGAPDEAAEALGSADCELSAKLLRRADLNQGIGEPQSPSRRVFNPYTEFKEFSRKQIKDMEKMFKQYDAGRDGFIDLMELKLMMEKLGAPQTHLGLKNMIKEVDEDFDSKLSFREFLLIFRKAAAGELQEDSGLCVLARLSEIDVSSEGVKGAKSFFEAKVQAINVSSRFEEEIKAEQEERKKQAEEMKQRKAAFKELQSTFK* |
| EFHD2-K188E | Atggccacggacgagctggccaccaagctgagccggcggctgcagatggagggcgagggcggcggcgagaccccggagcagcccgggctgaacggggcagcggcggcggcggcgggggcacccgacgaggcggccgaggcgctgggcagcgcggactgcgagctgagcgccaagctgctgcggcgcgcagacctcaaccagggcatcggcgagccccagtcgcccagccgccgcgtcttcaacccctacaccgagttcaaggagttctccaggaagcagatcaaggacatggagaagatgttcaagcagtatgatgccgggcgggacggcttcatcgacctgatggagctaaaactcatgatggagaaacttggggcccctcagacccacctgggcctgaaaaacatgatcaaggaggtggatgaggactttgacagcaagctgagcttccgggagttcctcctgatcttccgcaaggcggcggccggggagcttcaggaggacagcgggctgtgcgtgctggcccgcctctctgagatcgacgtctccagtgagggtgtcgagggggccaagagcttctttgaggccaaggtccaggccatcaacgtgtccagccgcttcgaggaggagatcaaggcagagcaggaggaaaggaagaagcaggcggaggagatgaagcagcggaaagcggccttcaaggagctgcagtccacctttaagtag  MATDELATKLSRRLQMEGEGGGETPEQPGLNGAAAAAAGAPDEAAEALGSADCELSAKLLRRADLNQGIGEPQSPSRRVFNPYTEFKEFSRKQIKDMEKMFKQYDAGRDGFIDLMELKLMMEKLGAPQTHLGLKNMIKEVDEDFDSKLSFREFLLIFRKAAAGELQEDSGLCVLARLSEIDVSSEGVEGAKSFFEAKVQAINVSSRFEEEIKAEQEERKKQAEEMKQRKAAFKELQSTFK* |
| EFHD2-K233E | Atggccacggacgagctggccaccaagctgagccggcggctgcagatggagggcgagggcggcggcgagaccccggagcagcccgggctgaacggggcagcggcggcggcggcgggggcacccgacgaggcggccgaggcgctgggcagcgcggactgcgagctgagcgccaagctgctgcggcgcgcagacctcaaccagggcatcggcgagccccagtcgcccagccgccgcgtcttcaacccctacaccgagttcaaggagttctccaggaagcagatcaaggacatggagaagatgttcaagcagtatgatgccgggcgggacggcttcatcgacctgatggagctaaaactcatgatggagaaacttggggcccctcagacccacctgggcctgaaaaacatgatcaaggaggtggatgaggactttgacagcaagctgagcttccgggagttcctcctgatcttccgcaaggcggcggccggggagcttcaggaggacagcgggctgtgcgtgctggcccgcctctctgagatcgacgtctccagtgagggtgtcaagggggccaagagcttctttgaggccaaggtccaggccatcaacgtgtccagccgcttcgaggaggagatcaaggcagagcaggaggaaaggaagaagcaggcggaggagatgaagcagcggaaagcggccttcgaggagctgcagtccacctttaagtag  MATDELATKLSRRLQMEGEGGGETPEQPGLNGAAAAAAGAPDEAAEALGSADCELSAKLLRRADLNQGIGEPQSPSRRVFNPYTEFKEFSRKQIKDMEKMFKQYDAGRDGFIDLMELKLMMEKLGAPQTHLGLKNMIKEVDEDFDSKLSFREFLLIFRKAAAGELQEDSGLCVLARLSEIDVSSEGVKGAKSFFEAKVQAINVSSRFEEEIKAEQEERKKQAEEMKQRKAAFEELQSTFK* |

**Supplementary Table 3 Sequence alignment showing that high conservation of the EFHD2 K9 site across species.**

| Species | Sequence |
| --- | --- |
| Larus maculipennis | LSRRYDAGKDGFIDLMELKL |
| Falco cachinnans | LSHRYDAGKDGFIDLMELKL |
| Plains-wanderer | LPHRYDAGKDGFIDLMELKL |
| Arctic skua | LSRRYDAGKDGFIDLMELKL |
| Parra jacana | LSHRYDAGKDGFIDLMELKL |
| Lepisosteus spatula | MATDELASKLTRRLQIEEGA |
| Cricetulus roborovskii | MATDELASKLSRRLQMEGEG |
| Philippine fairy-bluebird | LSHRYDAGKDGFIDLME |
| American pygmy kingfisher | LSHRYDAGKDGFIDLMELKL |
| Razorbill | LSHRYDAGKDGFIDLMELKL |
| Senegal bichir | MASDELANKLTRRLKIEEGA |
| Abyssinian ground-hornbill | LSHRYDAGKDGFIDLMELKL |
| Tringa interpres | LSRRYDAGKDGFIDLMELKL |
| Falco haliaetus | LSRRYDAGKDGFIDLMELKL |
| Atlantic salmon | MATDELSSKLSRRLQIEDGA |
| American beaver | MATDELATKLSRRLQMEGEG |
| Pongo pygmaeus abelii | MATDELATKLSRRLQMEGEG |
| Chimpanzee | MATDELATKLSRRLQMEGEG |
| Canis familiaris | MATDELASKLSRRLQLEDEG |
| Psetta maxima | MATDELSSKLSRRLQIEEGA |
| Clown anemonefish | MATDELTSKLSRRLQIEDGT |
| Chromis aureus | MATDELSSKLSRRLQIEEGA |
| Denticle herring | MASDELSSKLNRRLKIEEGG |
| Salmo tshawytscha | MATDELSSKLSRRLQIEDGA |
| Anthias testudineus | MATDELSSKLSRRLQIEEGT |
| Lesser Egyptian jerboa | MATDELASKLSRRLQMEGEG |
| Red-bellied piranha | MATDELSMKLNRRLKIEEGG |
| Northern pike | MATDELSSKLSRRLQIEEGV |
| Felis silvestris catus | MATDELASKLSRRLQMEGEG |
| Morone labrax | MATDELSSKLSRRLQIEEGA |
| Chromis callipterus | MATDELSSKLSRRLQIEEGS |
| Gymnotus electricus | MATNELSMKLNRRLQIEEGA |
| Spalax galili | MATDELASKLNRRLQMEGEG |
| Simia sabaea | MATDELATKLSRRLQMEGEG |
| Cercocebus torquatus atys | MATDELATKLSRRLQMEGEG |
| Japanese killifish | MATDELSSKLSRRLQIEDGA |
| Northern mallard | MELKLMMEKLGAPQTHIGLK |
| Common wombat | MATDELATKLNRRLQIEDGG |
| orbiculate cardinalfish | MATDELSNKLNRRLQIEDGT |
| Sciurus degus | MATDELATKLSRRLQMEGEG |
| Antarctic dragonfish | MATDELSSKLSRRLQIEEGA |
| Emys bellii | MATDELASKLGRRLQIEEEG |
| Goodes thornscrub tortoise | MATDELASKLSRRLQIEEEG |
| Eurasian red squirrel | MATDELATKLSRRLQMEGEG |
| Gelada baboon | MATDELATKLSRRLQMEGEG |
| Felis pardus | MATDELASKLSRRLQMEGEG |
| Sea lamprey | MATDELAGKLQRRLKREEED |
| Naked mole rat | MATDELATKLSRRLQMEGEG |
| Spermophilus tridecemlineatus | MATDELATKLSRRLQMEGEG |
| Astyanax fasciatus mexicanus | MATDELSQKLSRRLQIEDGS |
| Olive baboon | MATDELATKLSRRLQMEGEG |
| Cebus capucinus imitator | MATDELATKLSRRLQMEGEG |
| Propithecus verreauxi coquereli | MATDELATKLSRRLQMEGEG |
| Beluga whale | MATDELASKLSRRLQMEGEG |
| Live sharksucker | MATDELSSKLSRRLQIEEGA |
| Felis canadensis | MATDELASKLSRRLQMEGEG |
| Indian glassy fish | MAADELSSKLNRRLQIEEGA |
| Caecilia seraphini | MASDELANKLNRRLKIEEGE |
| Orycteropus afer afer | MATDELATKLNRRLQLEDEG |
| Chinchilla villidera | MATDELATKLSRRLQMEGEG |
| Equus africanus asinus | MATDELASKLSRRLQMEGEG |
| Mustela vison | MATDELASKLNRRLQMEDEG |
| Vaquita | MATDELASKLSRRLQMEDEG |
| Hatteria punctata | MATDELASKLNRRLQIEEDG |
| Cyrtodactylus macularius | MATEELASKLSRRLQVGERN |
| Garnett's greater bushbaby | MATDELATKLNRRLQMEGEG |
| Chinese alligator | MELKLMMEKLGAPQTHLGLK |
| Silurus punctatus | MASNELSAKLHRRLKIEEGE |
| Pig-tailed macaque | MATDELATKLSRRLQMEGEG |
| Yangtze river dolphin | MATDELASKLSRRLQMEGEG |
| Hybrid cattle | MATDELASKLSRRLQMEGEG |
| Duckbill platypus | MATDELATKLNRRLQMEGDG |
| Cheetah | MATDELASKLSRRLQMEGEG |
| Estuarine crocodile | MELKLMMEKLGAPQTHLGLK |
| Alpine marmot | MELKLMMEKLGAPQTHLGLK |
| Ugandan red Colobus | MATDELATKLSRRLQMEGEG |
| Osteoglossum formosum | MASDELASKLSRRLNIEDGV |
| Medium ground-finch | MELKLMMEKLGAPQTHLGLK |
| Cape golden mole | MASDELATKLSRRLQLEDEG |
| Bactrian camel | MELKLMMEKLGAPQTHLGLK |
| Pygathrix bieti | MATDELATKLSRRLQMEGEG |
| PPacific walrus | MATDELASKLNRRLQMEDEG |
| PFlorida manatee | MATDELATKLSRRLQLEDEG |
| Physeter catodon | MATDELASKLSRRLQMEGEG |
| Prairie deer mouse | MELKLMMEKLGAPQTHLGLK |
| Odocoileus virginianus texanus | MATDELASKLSRRLQMEGEG |
| California sealion | MATDELASKLNRRLQMEDEG |
| Delphinus truncatus | MATDELANKLSRRLQMEGEG |
| Atlantic herring | MATDELSSKLNRRLKIEEGG |
| Ricefield mouse | MATDELASKLSRRLQMEGEG |
| Felis concolor | MELKLMMEKLGAPQTHLGLK |
| Siamese fighting fish | MATDELSSKLSRRLQIEEGG |
| Microcaecilia unicolor | MASDELANKLNRRLRIEEGE |
| Sarcophilus laniarius | MATDELATKLNRRLQIEDGG |
| Great Himalayan leaf-nosed bat | MATDELASKLSRRLQMEEEG |
| Bos grunniens | MATDELASKLSRRLQMEGEG |
| Calamoichthys calabaricus | MASDELANKLTRRLKIEEGA |
| Moschus sibiricus | MATDELASKLSRRLQMEGEG |
| Indian cobra | MELKLMMEKLGAPQTHLGLK |
| Tupinambis merianae | MATDELTSKLNRRLLVEEGG |
| Salmo namaycush | MATDELSSKLSRRLQIEDGA |
| Python molurus bivittatus | MELKLMMEKLGAPQTHLGLK |
| Hylobates leucogenys | MATDELATKLSRRLQMEGEG |
| African elephant | MATDELATKLNRRLQLEDEG |
| Coelacanth | MATDELANKLNRRLKIEDGS |
| Tilapia nilotica | MATDELSSKLSRRLQIEEGA |
| Mustela furo | MATDELASKLNRRLQIEDEG |
| Ord's kangaroo rat | MELKLMMEKLGAPQTHLGLK |
| Pygathrix roxellana | MATDELATKLSRRLQMEGEG |
| Bonobo | MATDELATKLSRRLQMEGEG |
| Monachus schauinslandi | MATDELASKLNRRLQMEDEG |
| Yangtze finless porpoise | MATDELASKLSRRLQMEDEG |
| Balaenoptera davidsoni | MELKLMMEKLGAPQTHLGLK |
| Northern fur seal | MATDELASKLNRRLQMEDEG |
| Lacerta muralis | MATDELADKLSRRLQVEEHN |
| Eastern brown snake | MATEELAGKLSRRLRVEGGP |
| Gilthead sea bream | MATDELTSKLTRRLQIEEGA |
| Brown trout | MATDELSSKLSRRLQIEDGA |
| Thamnophis sirtalis | MATEELAGKLSRRLRVEGAP |
| Cebus apella | MATDELATKLSRRLQMEGEG |
| Camelus bactrianus ferus | MATDELASKLSRRLQMEGEG |
| Testudo abingdonii | MELKLMMEKLGASQTHLGLK |
| Chacoan peccary | MATDELASKLSRRLQMEGEG |
| Lepadogaster willdenowi | MATDELSSKLQRRLQIEDGD |
| Lemur murinus | MATDELASKLSRRLQMEGEG |
| Hapalemur simus | MATDELASKLSRRLQMEGEG |
| Spermophilus parryii | MATDELATKLSRRLQMEGEG |
| Thalarctos maritimus | MATDELASKLNRRLQMEDEG |
| Salmo gairdneri | MATDELSSKLSRRLQIEDGA |
| Elaphe guttata | MATEELAGKLSRRLRVEGAP |
| Rock dove | MELKLMMEKLGAPQTHLGLK |
| Tanaka's snailfish | MATDELSSKLSRRLQIEDGA |
| Tmainland tiger snake | MATEELAGKLSRRLRVEGGP |
| Macropteronotus magur | MASDELSAKLNRRMKIEEGE |
| Merluccius cadenati | MASDELSSKLNRRLQIEDGT |
| Western European hedgehog | MATDELASKLSRRLQMGGEG |
| Golden hamster | MATDELASKLSRRLQMEDEG |
| Greater horseshoe bat | MATDELASKLSRRLQMEGDG |
| Anas fuligula | MELKLMMEKLGAPQTHIGLK |
| Ceratodon monodon | MATDELASKLSRRLQMEGEG |
| Aphritis gobio | MATDELSSKLSRRLQIEEGA |
| Testudo serpentina | MATEELASKLSRRLQIEEEG |
| Cricetulus barabensis griseus | MATDELASKLSRRLQMEGEG |
| Acipenser oxyrinchus oxyrinchus | MASDELASKLTRRLKIEEGA |
| Zebu | MATDELASKLSRRLQMEGEG |
| Rhesus macaque | MATDELATKLSRRLQMEGEG |
| White-tufted-ear marmoset | MATDELATKLSRRLQMEGEG |
| Brachydanio rerio | MATDELSSKLNRRLKIEDGS |
| Pundamilia nyererei | MATDELSSKLSRRLQIEEGS |
| bicolor damselfish | MATDELSSKLNRRLQIEDGS |
| Holocentrus calcarifer | MATDELSSKLSRRLQIEEGA |
| pale spear-nosed bat | MATDELASKLSRRLQMEGEG |

**Supplementary Table 4 Oligonucleotide sequences used in this study.**

| Oligonucleotide name | Sequence (5' to 3') |
| --- | --- |
| shRNA-NC | TTCTCCGAACGTGTCACGT |
| hsa-let-7b-5p-overexpressing lentivirus | TGGGGAAACTGAGGCCCAGAGAGCCAGGGACTTCCCAAGACCAGCCAGCAGCAGCTGCCCCTTCCTGGGGTGCCATCTCCCCTGTCCCTCCTGCCCTGCGCCTGCCCAGCCCTCCTGCTCTGGTGACTGAGGACCGCCAGGCAGGGGCTGGTGCTGGGCGGGGGGCGGCGGGCCCTCCCGCAGTGCAAGGCCGGGCCTGGCGGGGTGAGGTAGTAGGTTGTGTGGTTTCAGGGCAGTGATGTTGCCCCTCGGAAGATAACTATACAACCTACTGCCTTCCCTGAGGAGCCCAGTGACACGACCCCATGGGAGGGCCGCCCCCTACCTCAGTGACACGACCCCACGGGAGGGCTGCCCCCCACCTCAGTGACCTGCAGGGGGCCTGAGCCGAAGCTGGGTGGGCATCTGGGAGCTAGATTCAATAAAGCTGTTCTGACCATGAACTTGGAACTGGCCCCTTTCATTCTGGAAGCCCAGGGACTG |
| shEFHD2#1 | GAGAAGATGTTCAAGCAGTAT |
| shEFHD2#2 | GCCTTAGAATCTGCAGAAATT |
| shEFHD2#3 | GATCAAGGACATGGAGAAGAT |
| EFHD2-overexpressing lentivirus | ATGGCCACGGACGAGCTGGCCACCAAGCTGAGCCGGCGGCTGCAGATGGAGGGCGAGGGCGGCGGCGAGACCCCGGAGCAGCCCGGGCTGAACGGGGCAGCGGCGGCGGCGGCGGGGGCACCCGACGAGGCGGCCGAGGCGCTGGGCAGCGCGGACTGCGAGCTGAGCGCCAAGCTGCTGCGGCGCGCAGACCTCAACCAGGGCATCGGCGAGCCCCAGTCGCCCAGCCGCCGCGTCTTCAACCCCTACACCGAGTTCAAGGAGTTCTCCAGGAAGCAGA  TCAAGGACATGGAGAAGATGTTCAAGCAGTATGATGCCGGGCGGGACGGCTTCATCGACCTGATGGAGCTAAAACTCATGATGGAGAAACTTGGGGCCCCTCAGACCCACCTGGGCCTGAAAAACATGATCAAGGAGGTGGATGAGGACTTTGACAGCAAGCTGAGCTTCCGGGAGTTCCTCCTGATCTTCCGCAAGGCGGCGGCCGGGGAGCTTCAGGAGGACAGCGGGCTGTGCGTGCTGGCCCGCCTCTCTGAGATCGACGTCTCCAGTGAGGGTGT  CAAGGGGGCCAAGAGCTTCTTTGAGGCCAAGGTCCAGGCCATCAACGTGTCCAGCCGCTTCGAGGAGGAGATCAAGGCAGAGCAGGAGGAAAGGAAGAAGCAGGCGGAGGAGATGAAGCAGCGGAAAGCGGCCTTCAAGGAGCTGCAGTCCACCTTTAAGTAG |
| HMGB1-overexpressing lentivirus | ATGGGCAAAGGAGATCCTAAGAAGCCGAGAGGCAAAATGTCATCATATGCATTTTTTGTGCAAACTTGTCGGGAGGAGCATAAGAAGAAGCACCCAGATGCTTCAGTCAACTTCTCAGAGTTTTCTAAGAAGTGCTCAGAGAGGTGGAAGACCATGTCTGCTAAAGAGAAAGGAAAATTTGAAGATATGGCAAAAGCGGACAAGGCCCGTTATGAAAGAGAAATGAAAACCTATATCCCTCCCAAAGGGGAGACAAAAAAGAAGTTCAAGGATCCCAATG  CACCCAAGAGGCCTCCTTCGGCCTTCTTCCTCTTCTGCTCTGAGTATCGCCCAAAAATCAAAGGAGAACATCCTGGCCTGTCCATTGGTGATGTTGCGAAGAAACTGGGAGAGATGTGGAATAACACTGCTGCAGATGACAAGCAGCCTTATGAAAAGAAGGCTGCGAAGCTGAAGGAAAAATACGAAAAGGATATTGCTGCATATCGAGCTAAAGGAAAGCCTGATGCAGCAAAAAAGGGAGTTGTCAAGGCTGAAAAAAGCAAGAAAAAGAAGGAAGA  GGAGGAAGATGAGGAAGATGAAGAGGATGAGGAGGAGGAGGAAGATGAAGAAGATGAAGATGAAGAAGAAGATGATGATGATGAATAA |
| siRNA-NC | UUCUCCGAACGUGUCACGUdTdT |
| siHMGB1#1 | CCCAGAUGCUUCAGUCAACUUdTdT |
| siHMGB1#2 | CAAGGCCCGUUAUGAAAGAdTdT |
| siHMGB1#3 | CCUGUCCAUUGGUGAUGUUdTdT |

**Supplementary Table 5** **qRT-PCR primer sequences.**

| Primer | 5' to 3' |
| --- | --- |
| U6-F (human) | CTCGCTTCGGCAGCACA |
| U6-R (human) | AACGCTTCACGAATTTGCGT |
| hsa-let-7b-5p-F | GCCGCGTGAGGTAGTAGGTTGT |
| hsa-let-7b-5p-R | AGTGCAGGGTCCGAGGTATT |
| hsa-let-7b-5p-RT | GTCGTATCCAGTGCAGGGTCCGAGGTATTCGCACTGGATACGACAACCAC |
| β-actin-F (human) | GAGAAAATCTGGCACCACACC |
| β-actin-R (human) | GGATAGCACAGCCTGGATAGCAA |
| EFHD2-F (human) | CGGCTTCATCGACCTGATGG |
| EFHD2-R (human) | TGCGGAAGATCAGGAGGAAC |

**References**

1.	Beyer M, Mallmann MR, Xue J, Staratschek-Jox A, Vorholt D, Krebs W*, et al.* High-resolution transcriptome of human macrophages. *PLoS One* 2012, **7**(9)**:** e45466.

2.	Gaedcke J, Grade M, Camps J, Sokilde R, Kaczkowski B, Schetter AJ*, et al.* The rectal cancer microRNAome--microRNA expression in rectal cancer and matched normal mucosa. *Clin Cancer Res* 2012, **18**(18)**:** 4919-4930.

3.	Zhang H, Xue C, Shah R, Bermingham K, Hinkle CC, Li W*, et al.* Functional analysis and transcriptomic profiling of iPSC-derived macrophages and their application in modeling Mendelian disease. *Circ Res* 2015, **117**(1)**:** 17-28.

4.	Derlindati E, Dei Cas A, Montanini B, Spigoni V, Curella V, Aldigeri R*, et al.* Transcriptomic analysis of human polarized macrophages: more than one role of alternative activation? *PLoS One* 2015, **10**(3)**:** e0119751.

5.	Nagy ZB, Wichmann B, Kalmar A, Galamb O, Bartak BK, Spisak S*, et al.* Colorectal adenoma and carcinoma specific miRNA profiles in biopsy and their expression in plasma specimens. *Clin Epigenetics* 2017, **9:** 22.

6.	Moganti K, Li F, Schmuttermaier C, Riemann S, Kluter H, Gratchev A*, et al.* Hyperglycemia induces mixed M1/M2 cytokine profile in primary human monocyte-derived macrophages. *Immunobiology* 2017, **222**(10)**:** 952-959.

7.	Mossel DM, Moganti K, Riabov V, Weiss C, Kopf S, Cordero J*, et al.* Epigenetic Regulation of S100A9 and S100A12 Expression in Monocyte-Macrophage System in Hyperglycemic Conditions. *Front Immunol* 2020, **11:** 1071.

8.	Chen Z, Ren R, Wan D, Wang Y, Xue X, Jiang M*, et al.* Hsa_circ_101555 functions as a competing endogenous RNA of miR-597-5p to promote colorectal cancer progression. *Oncogene* 2019, **38**(32)**:** 6017-6034.

9.	Gutbier S, Wanke F, Dahm N, Rummelin A, Zimmermann S, Christensen K*, et al.* Large-Scale Production of Human iPSC-Derived Macrophages for Drug Screening. *Int J Mol Sci* 2020, **21**(13).

10.	Bezie S, Freuchet A, Serazin C, Salama A, Vimond N, Anegon I*, et al.* IL-34 Actions on FOXP3(+) Tregs and CD14(+) Monocytes Control Human Graft Rejection. *Front Immunol* 2020, **11:** 1496.

11.	Trauelsen M, Hiron TK, Lin D, Petersen JE, Breton B, Husted AS*, et al.* Extracellular succinate hyperpolarizes M2 macrophages through SUCNR1/GPR91-mediated Gq signaling. *Cell Rep* 2021, **35**(11)**:** 109246.

12.	Silva-Bermudez LS, Sevastyanova TN, Schmuttermaier C, De La Torre C, Schumacher L, Kluter H*, et al.* Titanium Nanoparticles Enhance Production and Suppress Stabilin-1-Mediated Clearance of GDF-15 in Human Primary Macrophages. *Front Immunol* 2021, **12:** 760577.

13.	Sedivy-Haley K, Blimkie T, Falsafi R, Lee AH, Hancock REW. A transcriptomic analysis of the effects of macrophage polarization and endotoxin tolerance on the response to Salmonella. *PLoS One* 2022, **17**(10)**:** e0276010.

14.	Hanlon MM, Smith CM, Canavan M, Neto NGB, Song Q, Lewis MJ*, et al.* Loss of synovial tissue macrophage homeostasis precedes rheumatoid arthritis clinical onset. *Sci Adv* 2024, **10**(39)**:** eadj1252.

15.	Newman AM, Liu CL, Green MR, Gentles AJ, Feng W, Xu Y*, et al.* Robust enumeration of cell subsets from tissue expression profiles. *Nat Methods* 2015, **12**(5)**:** 453-457.

16.	Racle J, de Jonge K, Baumgaertner P, Speiser DE, Gfeller D. Simultaneous enumeration of cancer and immune cell types from bulk tumor gene expression data. *Elife* 2017, **6**.

17.	Finotello F, Mayer C, Plattner C, Laschober G, Rieder D, Hackl H*, et al.* Molecular and pharmacological modulators of the tumor immune contexture revealed by deconvolution of RNA-seq data. *Genome Med* 2019, **11**(1)**:** 34.

18.	Becht E, Giraldo NA, Lacroix L, Buttard B, Elarouci N, Petitprez F*, et al.* Estimating the population abundance of tissue-infiltrating immune and stromal cell populations using gene expression. *Genome Biol* 2016, **17**(1)**:** 218.

19.	Aran D, Hu Z, Butte AJ. xCell: digitally portraying the tissue cellular heterogeneity landscape. *Genome Biol* 2017, **18**(1)**:** 220.

20.	Yoshihara K, Shahmoradgoli M, Martinez E, Vegesna R, Kim H, Torres-Garcia W*, et al.* Inferring tumour purity and stromal and immune cell admixture from expression data. *Nat Commun* 2013, **4:** 2612.

21.	Li B, Severson E, Pignon JC, Zhao H, Li T, Novak J*, et al.* Comprehensive analyses of tumor immunity: implications for cancer immunotherapy. *Genome Biol* 2016, **17**(1)**:** 174.

22.	Zheng X, Song J, Yu C, Zhou Z, Liu X, Yu J*, et al.* Single-cell transcriptomic profiling unravels the adenoma-initiation role of protein tyrosine kinases during colorectal tumorigenesis. *Signal Transduct Target Ther* 2022, **7**(1)**:** 60.

23.	Wang H, Gong P, Chen T, Gao S, Wu Z, Wang X*, et al.* Colorectal Cancer Stem Cell States Uncovered by Simultaneous Single-Cell Analysis of Transcriptome and Telomeres. *Adv Sci (Weinh)* 2021, **8**(8)**:** 2004320.

24.	Guo W, Zhang C, Wang X, Dou D, Chen D, Li J. Resolving the difference between left-sided and right-sided colorectal cancer by single-cell sequencing. *JCI Insight* 2022, **7**(1).

25.	Li J, Wu C, Hu H, Qin G, Wu X, Bai F*, et al.* Remodeling of the immune and stromal cell compartment by PD-1 blockade in mismatch repair-deficient colorectal cancer. *Cancer Cell* 2023, **41**(6)**:** 1152-1169 e1157.

26.	Zhou Y, Zhang X, Gao Y, Peng Y, Liu P, Chen Y*, et al.* Neuromedin U receptor 1 deletion leads to impaired immunotherapy response and high malignancy in colorectal cancer. *iScience* 2024, **27**(7)**:** 110318.

27.	Guo L, Wang Y, Yang W, Wang C, Guo T, Yang J*, et al.* Molecular Profiling Provides Clinical Insights Into Targeted and Immunotherapies as Well as Colorectal Cancer Prognosis. *Gastroenterology* 2023, **165**(2)**:** 414-428 e417.

28.	Wang F, Long J, Li L, Wu ZX, Da TT, Wang XQ*, et al.* Single-cell and spatial transcriptome analysis reveals the cellular heterogeneity of liver metastatic colorectal cancer. *Sci Adv* 2023, **9**(24)**:** eadf5464.

29.	Hsu WH, LaBella KA, Lin Y, Xu P, Lee R, Hsieh CE*, et al.* Oncogenic KRAS Drives Lipofibrogenesis to Promote Angiogenesis and Colon Cancer Progression. *Cancer Discov* 2023, **13**(12)**:** 2652-2673.

30.	Yang M, Yang C, Ma D, Li Z, Zhao W, Yang D. Single-cell analysis reveals cellular reprogramming in advanced colon cancer following FOLFOX-bevacizumab treatment. *Front Oncol* 2023, **13:** 1219642.

31.	Zhou X, Lin J, Shao Y, Zheng H, Yang Y, Li S*, et al.* Targeting PLCG2 Suppresses Tumor Progression, Orchestrates the Tumor Immune Microenvironment and Potentiates Immune Checkpoint Blockade Therapy for Colorectal Cancer. *Int J Biol Sci* 2024, **20**(14)**:** 5548-5575.

32.	Zhang J, Mizuuchi Y, Ohuchida K, Hisano K, Shimada Y, Katayama N*, et al.* Exploring the tumor microenvironment of colorectal cancer patients post renal transplantation by single-cell analysis. *Cancer Sci* 2025, **116**(2)**:** 500-512.

33.	Hao Y, Stuart T, Kowalski MH, Choudhary S, Hoffman P, Hartman A*, et al.* Dictionary learning for integrative, multimodal and scalable single-cell analysis. *Nat Biotechnol* 2024, **42**(2)**:** 293-304.

34.	Qiu X, Mao Q, Tang Y, Wang L, Chawla R, Pliner HA*, et al.* Reversed graph embedding resolves complex single-cell trajectories. *Nat Methods* 2017, **14**(10)**:** 979-982.

35.	Jin S, Plikus MV, Nie Q. CellChat for systematic analysis of cell-cell communication from single-cell transcriptomics. *Nat Protoc* 2025, **20**(1)**:** 180-219.
